# Towards unmanned proteomics data generation: a fully automated sample-to-data system for proteomic experiments

**DOI:** 10.1038/s41421-025-00844-7

**Published:** 2025-10-29

**Authors:** Dongxue Wang, Wendong Chen, Linhai Xie, Ying Xu, Chuanxi Huang, Yuanyuan Liu, Xi Wang, Xiaowei Huang, Keren Zhang, Mengting Pan, Shaozhen Wang, Jing Yang, Liujun Tang, Ruijun Tian, Fuchu He

**Affiliations:** 1 State Key Laboratory of Medical Proteomics, Beijing Proteome Research Center, National Center for Protein Sciences (Beijing), Research Unit of Proteomics-driven Cancer Precision Medicine (Chinese Academy of Medical Sciences), Beijing Institute of Lifeomics, Beijing, China; 2 International Academy of Phronesis Medicine (Guangdong), Guangzhou, Guangdong China; 3 State Key Laboratory of Medical Proteomics and Shenzhen Key Laboratory of Functional Proteomics, Department of Chemistry and Research Center for Chemical Biology and Omics Analysis, School of Science and Guangming Advanced Research Institute, Southern University of Science and Technology, Shenzhen, Guangdong China; 4 Thermo Fisher Scientific (China) Co., Ltd, Shanghai, China; 5 Guangzhou National Laboratory, Guangzhou International Bio Island, Guangzhou, Guangdong China

**Keywords:** Proteomic analysis, Proteomics

Dear Editor,

Data-driven biology requires comprehensive and high-quality data at all molecular levels. Liquid chromatography-mass spectrometry (LC-MS)-based proteomics has emerged as the leading method for comprehensively identifying and quantifying proteins in biological samples. Many studies have highlighted the value of having large-scale MS-based proteomic datasets (i.e., from hundreds to more than one thousand measurements) to promote our understanding of human biology and various complex diseases. These efforts have therefore inspired the international community to launch the π-HuB (The Proteomic Navigator of the Human Body) project^1^, aiming to transform biology and medicine in the way that the Human Genome Project did^2^.

Similar to other data-driven big-science projects, π-HuB will start by manufacturing massive data. Although a single LC-MS/MS run can profile up to 10,000 proteins^3,4^, which is comparable to the depth achieved by advanced genome sequencing, a key challenge is to increase throughput and data robustness in proteomic measurements without compromising the depth of identification and the quantitative precision^5,6^. In recent years, we have observed numerous technical advancements in high-throughput proteomics, particularly in automated sample preparation and LC-MS/MS technologies. For example, automated sample processing with liquid-handling systems enables the preparation of hundreds of low-input samples each day^7–9^. Moreover, rapid chromatographic separations and MS/MS data acquisitions have been achieved by implementing new instruments and/or settings, allowing the analytical throughput of > 100 samples per day (SPD) on a single LC-MS system^10,11^.

Despite significant advancements, the demand for the π-HuB to generate data from millions of samples underscores an urgent need to improve the efficiency and reliability of proteomics data production. First, there is a necessity for a fully automated sample-to-data system that operates without human intervention, eliminating errors or variabilities caused by personnel or batch differences. Second, achieving ultra-high-throughput data generation is crucial to reduce the turnaround time from sample preparation to data processing. Lastly, increasing throughput in proteomic measurements should not compromise the depth of profiling or quantitative precision. To address all these needs simultaneously, we present the pilot π-HuB data factory, which features a fully automated sample-to-data system, referred to as the π-Station, designed to facilitate high-throughput proteomics in a nearly unmanned manner (Supplementary Video S1).

Previous automation efforts in LC-MS/MS-based proteomics have primarily concentrated on sample preparation with manual assistance.^12^ The entire process, from sample to data, still requires significant hands-on experience from skilled scientists (Fig. 1a). Therefore, we designed and set up the π-Station that can seamlessly integrate fully automated sample preparation with LC-MS/MS instrumentation and computing servers, enabling the direct generation of protein quantification data matrices from biospecimen samples without manual intervention (Fig. 1b). π-Station is a linear, expandable and connectable platform, consisting of 14 customized devices from seven different vendors, and seamlessly connected to LC-MS/MS systems via 2 robotic arms (Fig. 1c). All hardwares are configured in and managed by the Momentum Workflow Scheduling Software, enabling modular method editing of both sample preparation and LC-MS/MS data acquisition (Supplementary Fig. S1a). Meanwhile, data storage, processing, quality control (QC), and monitoring modules can be achieved by controlling external proteomic software tools and social media applications via in-house UI tools developed in Python.

**Fig. 1:**
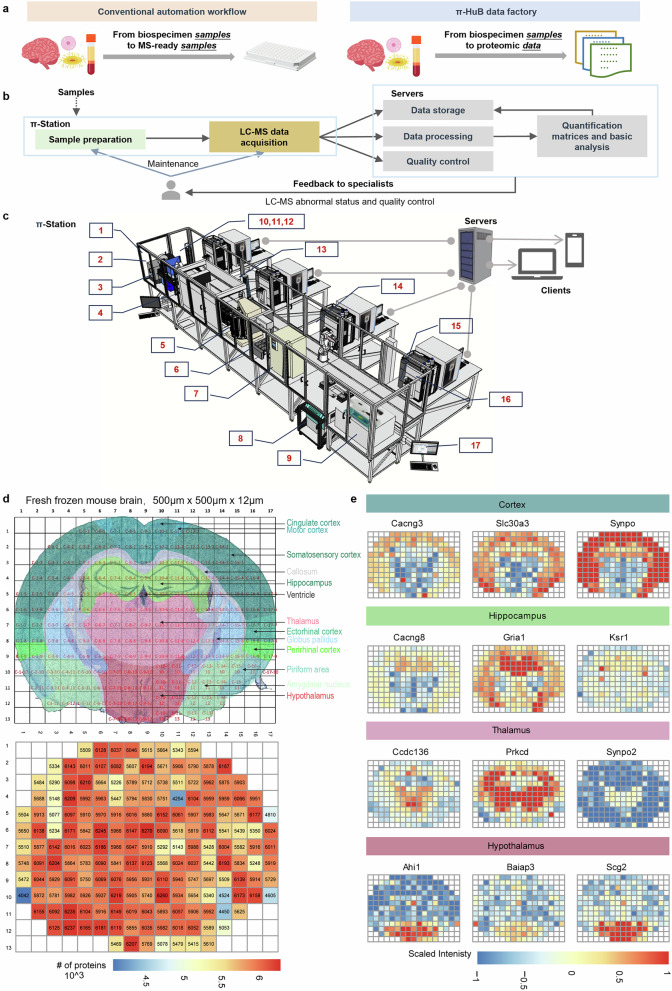
Overview of the π-HuB data factory and spatial proteomics results. **a** Comparison of the conventional automated proteomic workflow and our sample-to-data fully automated workflow. **b** Flowchart of the π-HuB data factory. Samples are loaded into the π-Station, where they are automatically prepared into peptides and subjected to LC-MS/MS analysis. The data is automatically stored, processed, and undergoes QC. If any abnormalities are detected and once QC information is extracted, the system will notify specialists via a social App or email to facilitate prompt maintenance and prevent sample loss. **c** Overview of the prototype of π-HuB data factory. It mainly consists of π-Station, LC-MS/MS instrumentation, and computing servers. π-Station is the fully automated sample preparation module, which is coupled to the LC-MS/MS systems. This prototype includes the following hardware and software: (1) LE220Rsc focused-ultrasonicator (Covaris); (2) ALPS 3000 automated microplate heat sealer (Thermo Fisher Scientific); (3) XPeel automated plate seal remover (Brooks); (4) Biomek i7 automated liquid handler with the Amplius positive pressure extractor (Beckman Colter); (5) Cytomat 10 hotel (Thermo Fisher Scientific); (6) AssayMAP Bravo Protein Sample Prep Platform (Agilent); (7) Cytomat 2C425 automated incubator (Thermo Fisher Scientific); (8) Rotanta 460 automated centrifuge (Hettich); (9) CombiDancer vortex vacuum concentrator (Hettich); (10) Multiskan SkyHigh microplate spectrophotometer (Thermo Fisher Scientific); (11) Automated Thermal Cycler (Thermo Fisher Scientific); (12) Incubator Shaker DWP (Inheco); (13) Spinnaker microplate robot (Thermo Fisher Scientific); (14) F7 Industrial Robot (Thermo Fisher Scientific); (15) Vanquish UHPLC Loader (Thermo Fisher Scientific); (16) LC-MS system, Vanquish Neo UHPLC system coupled to Orbitrap Exploris 480 mass spectrometer (Thermo Fisher Scientific); (17) Momentum workflow scheduling software (Thermo Fisher Scientific). **d** Results of the spatial proteomic analysis. The upper panel illustrates the scheme of the coronal section of the mouse brain, and the lower panel indicates the number of proteins identified in each micro-specimen. The color scale ranges from 4 (blue) to 6.5 (red). **e** Maps of region marker proteins, which correlated well with the literature. The color scale is from –1.0 (blue) to 1.0 (red).

At π-Station, a typical proteomic workflow includes protein extraction, digestion, desalting, solvent evaporation, resuspension of dried MS-ready peptide, and initiation of LC-MS/MS analysis. The devices utilized to perform these steps are described in Supplementary Fig. S1b. With the Momentum software, a workflow can be executed both individually and efficiently in multiple iterations. Here, an iteration refers to one complete cycle of the automated workflow. Taking the process of plasma or cell line analysis as example, a single iteration takes 7.5 h to process 96 samples into MS-ready peptides, while it only takes 1.5 h for the second and the following iterations, resulting in finishing 384 samples in 4 iterations within 12 h (Supplementary Fig. S4). Supplementing labware and reagents allows for infinite iterations of the whole process. Once the MS-ready peptides are made, the Momentum software will instruct robotic arms to send them to the sample loaders to initiate LC-MS/MS data acquisition on multiple instruments. In the pilot π-HuB data factory, the analytical capacity can be further enhanced by paralleling data acquisition on 15 LC-MS/MS instruments, significantly increasing turnaround rates for digitizing biospecimen samples into proteomic data. Our platform enables the highest throughput of 360 SPD using 60-min standard gradients, and thus those samples automatically prepared from 4-iteration settings can be measured within nearly a single day.

The ultra-high-throughput sample-to-data workflow enabled by the π-Station poses new challenges for continuously monitoring system performance that requires extensive manual inspection by experienced specialists. To address this bottleneck, in addition to implementing an effective QC strategy (Supplementary Fig. S3), we developed a computational framework called π-ProteomicInfo for automating data storage, processing LC-MS/MS data, and monitoring the performance and status of LC-MS/MS instruments (Supplementary Fig. S2a). The process starts with the monitor module, a standalone program installed on computers that control LC-MS/MS systems. This module monitors instrument status (e.g., warnings and errors) and automatically transfers raw data files once each run is completed. After the data transfer, the analyzer module is triggered to generate qualitative and quantitative profiles for each run and all experimental runs, and the QC module initiates extraction of QC metrics for data quality assessments (see Supplementary information for details). If QC data is found to be unqualified, the controller module will immediately stop data acquisition by sending commands to the instrument. This action helps prevent the irretrievable loss of precious clinical biopsies typically with very limited amounts. Meanwhile, the specialists operating the corresponding instruments will receive notifications via text messages about the QC results and instrument status, allowing them to perform maintenance as soon as possible (Supplementary Fig. S2b–d). This framework thus enables automatic feedback control of our platform’s performance and significantly reduces the data processing time.

To evaluate the π-Station, we conducted data-independent acquisition (DIA)‌ analyses of HEK293T cells with protein inputs ranging from 500 ng to 20 μg (Supplementary Fig. S5). For comparison, we applied two LC-MS/MS settings: a high-throughput method on a Thermo Exploris 480 with 60 min runs and a sensitive method on a Bruker timsTOF Pro with ~90 min runs (detailed configurations in Supplementary information). We quantified 3175 ± 160 (mean ± SD, the same as below) proteins with the high-throughput LC-MS/MS method and 8144 ± 429 with the sensitive LC-MS/MS method from 500 ng cell lysates. The depth and stability of analysis further improved as the protein input increased. We quantified over 7500 proteins with median coefficients of variation (CVs) < 8% from 5 μg samples using both high-throughput and sensitive 1-h LC-MS/MS analysis. To evaluate the long-term stability of this platform, we analyzed ten different cell line samples across 63 days in a total of 18 iterations using HEK293T cell lysates for QC. In each iteration, 6 QC samples were randomly placed on the 96-well plate. The variation in protein and precursor identification of QC samples across each iteration, intra-day iterations, and throughout the two-month period remained below 3% and 6%, respectively, with the maximum median CV of protein abundance remaining under 8% (Supplementary Fig. S6). The robustness of our platform was further demonstrated by analyzing ten widely-used cell lines. On average, we quantified 7770–8400 proteins across 22–720 biological replicates for each cell line, with a maximum median CV of 10.35%. These data depicted the biological features and differences across multiple cell lines (Supplementary Fig. S7). Together, these benchmarking results demonstrate the throughput and robustness of this pilot data factory for π-HuB, which is primarily facilitated by the fully automated sample-to-data system and parallel data acquisition.

Next, we sought to demonstrate the utility of our platform for human plasma profiling. We implemented a 48 SPD DIA method to analyze naïve plasma samples from a clinical cohort of 398 patients diagnosed with liver cancer. The samples were processed over five iterations and analyzed within two weeks, with π-ProteomicInfo overseeing these processes. In each iteration, six healthy plasma samples were included for QC. The analysis of the 28 QC samples showed the high reproducibility of our platform: 5797 ± 73 precursors, 238 ± 12 proteins, median CV of 4.36% inter-iterations. As a result, we quantified an average of 497 proteins in this liver cancer cohort, and there were no obvious batch effects between different iterations (Supplementary Fig. S8). Notably, in this case study, all MS data were collected in standard DIA settings. When coupled with more advanced DIA methods and/or MS instruments^10,13^, it could be foreseen that both the depth and throughput of our platform for plasma proteome profiling would further increase.

Encouraged by these results, we integrated SISPTOT (Simple and Integrated Spin-Tip-Based Proteomics Technology), a miniaturized kit for low-input sample preparation^14^, to further evaluate our capability in spatial proteomic analysis (Supplementary Fig. S9). The workflow allows for the processing of two iterations of sample preparation, totaling 192 laser capture microdissection (LCM) samples every 5.5 h, and can operate in infinite mode. For QC, 6 aliquots of 250 ng of mouse brain lysates were used as QC samples, prepared and analyzed alongside tissue slices in each iteration. As a result, the analysis achieved low intra- and inter-iteration CV for protein abundance values (median CV at 13.9%, *n* = 14) and reproducible quantification of precursors (65,806 ± 3737) and proteins (5740 ± 117) (Supplementary Fig. S10). Moreover, we generated a spatially resolved mouse brain proteome atlas of a 12-μm coronal section at the resolution of 500 μm × 500 μm. Proteomic profiling by 60 min DIA measurement resulted in the identification and quantification of a total of 7300 protein groups in 184 samples with an average of 5814 ( ± 378 SD) protein groups per sample at a false discovery rate of < 1% (Fig. 1d). Notably, our analysis correctly recapitulated the expected distributions of known protein markers in various anatomical regions (Fig. 1e). For example, Cacng3, Slc30a3, and Synpo in the cortex, Ahi1, Baiap3, and Scg2 in the hypothalamus, Ccdc136, Prkcd, and Synpo2 in the thalamus, Ahi1, Baiap3, and Scg2 in the hypothalamus, agreed well with their previously-reported distributions. Together, these results demonstrate that our platform achieves reproducible, large-scale, and high-resolution spatial proteomic profiling, as illustrated by the mouse brain atlas.

In summary, we developed the π-Station, a fully automated proteomic analysis platform equipped with feedback control systems. This platform enables ultra-high-throughput generation of high-quality proteomic data from microscale samples with minimal human intervention, greatly increasing productivity and efficiency while reducing errors and costs. It is operating as a core component of the π-HuB data factory, a key pillar of the π-HuB project. Up to date, it has enabled the generation of > 40,000 high-quality proteomic datasets, supporting dozens of studies funded by the pilot initiatives of π-HuB. Considering that a large monetary investment is required, this platform, at least in its current format, might be cost-prohibitive for individual academic laboratories or core facilities built at most research institutions. Nonetheless, the informatics pipeline for feedback control of systems performance can be easily adopted by any proteomics laboratory right away, allowing prompt maintenance of the LC-MS/MS system and implementing a stop-loss strategy immediately. We have applied it at the proteomic core facility at the National Center for Protein Science in Beijing. Furthermore, the flexible nature of this platform allows for future adaptations to various π-HuB pursuits (e.g., post-translational modifications, protein–protein interactions, and perturbation experiments, and so forth), although it is currently primarily applied to global proteome profiling of biofluids and tissues. Our future endeavors using this platform will mainly focus on producing multi-layer proteomics data with those samples from the π-HuB project. Meanwhile, we are now developing a more compact and cost-effective platform to facilitate the establishment of unmanned proteomics laboratories worldwide for π-HuB and beyond.

## Supplementary information

Supplemental information

Supplementary Data S1

Supplementary Video S1

## Data Availability

The proteomic data have been deposited in the ProteomeXchange Consortium (http://proteomecentral.proteomexchange.org) via the iProX partner repository^15^ with the dataset identifier PXD064080. Source data (for Fig. 1 and Supplementary Figs. S5–S10) are provided as Supplementary Data S1.
