# Supplementary material for: Towards unmanned proteomics data generation: a fully automated sample-to-data system for proteomic experiments
Source: Cell Discov. 2025 Oct 29;11:86. doi: 10.1038/s41421-025-00844-7 (PMC12572148; doi:10.1038/s41421-025-00844-7)
Supplement: Supplementary file 1 — Supplemental information [file 41421_2025_844_MOESM1_ESM.pdf]

Supplementary information

Towards Unmanned Proteomics Data Generation: A Fully Automated Sample-to-Data System for Proteomic Experiments

Dongxue Wang[1,2,], Wendong Chen[2], Linhai Xie[1,2], Ying Xu[2], Chuanxi Huang[2], Yuanyuan Liu[2], Xi Wang[3], Xiaowei Huang[2], Keren Zhang[3], Mengting Pan[2], Shaozhen Wang[5], Jing Yang[1,2,4], Liujun Tang[1,2]*, Ruijun Tian[3]*, Fuchu He[1,2]*

Methods

Mouse tissue collection

All mice used in this study were bred and housed under institutionally approved conditions in the Animal Experiments Center at Southern University of Science and Technology. All experimental procedures were conducted following approval from the Institutional Animal Care and Use Committee at Southern University of Science and Technology in China. Mouse brains were collected from 6- to 8-week-old C57BL/6 mice.

Plasma sample collection

We collected a total of 398 plasma samples from patients and healthy plasma from donors. Whole blood was collected into EDTA-treated tubes. Plasma was obtained by centrifugation at 2,000 x g for 15 minutes within 4 hours of collection. Plasma samples were stored at -80°C until processing. All experiments were conducted with the approval of the Research Ethics Committee and according to the Declaration of Helsinki.

Cell culture

In the methods development, evaluation, and quality control segments, we used ten different types of cell lines. HEK 293T, HeLa, HepG2, Huh7, MCF7, and MDA-MB-231 cells were cultured in Dulbecco's Modified Eagle Medium (DMEM; Gibco), supplemented with 10% fetal bovine serum (FBS) and 1% penicillin-streptomycin (Gibco). H1975, SNU475, and PLC cells were cultivated in RPMI-1640 medium (Gibco) supplemented with 10% FBS. Additionally, A549 cells were grown in an F-12K nutrient mixture (Gibco), supplemented with 10% FBS and 1% penicillin/streptomycin. All cells were cultured at 37 °C in a 5% CO_2 incubator with

95% air humidity. The cells were harvested when they reached approximately 80% confluence. After harvesting, the cells were washed three times with phosphate-buffered saline (PBS) and then stored at -80 °C for future processing.

Sample preparation of LCM samples

A fresh frozen mouse brain was embedded in Tissue OCT-Freeze Medium (Sakura Finetek USA, Inc.) and sliced into coronal sections with a thickness of 12 µm by using a Leica CM 1900 cryostat (Leica) at −20 °C. The sections were flat mounted onto membrane-coated glass slides (2.0 µm, PEN-membrane, Leica) and fixed by ice-frozen methanol for 10min. Then, the fixed sections were subjected to hematoxylin-eosin staining (Servicebio) and dehydration via an ethanol series. After being scanned using a Leica DM 2500 microscope at 5× magnification, the coronal sections were dissected into spots with a length of 500µm and a width of 500µm using an LMD7 Laser Microdissection Microscope (Leica). Dissected tissue sections were collected into 0.2 mL microtubes (Axygen) were lysed in 50µL of lysis buffer (1% DDM, 10mM HEPES, 150mM NaCl, 600mM guanidine HCl, and 1% Roche protease inhibitor mixture, at pH 7.4) via ultrasonication by a Q800R3 minsonicator (Qsonica) for 15 min (20s on, 20s off, 85% amplitude) at 4 °C. Then the samples were heated in a metal bath at 95 °C for 60 min. After centrifugation, the supernatant was transferred to a new 96-well plate for automated sample preparation.

The sample preparation process using autoSISPROT was effectively carried out on the Π-Station with disposable SISPROT-based cartridges using reagents from the SISPROT kit. This workflow consists of 6 essential steps: (1) Acidification: Begin by acidifying the samples with formic acid utilizing the Biomek i7. Add 5µL of Buffer Acidize to the sample and mix thoroughly by pipetting up and down 20 times. (2) Preparation of SISPROT-based cartridges: Activate the SISPROT-based cartridges with 60µL of Buffer Activate, followed by equilibration using 60µL of Buffer Wash on the Bravo system. (3) Sample Loading and Reduction: Load the samples onto the cartridges,

followed by washing with 30µL of Buffer Wash and 30µL of Buffer Activate. Next, reduce samples with 30µL of Buffer Induce in the dark for 15 minutes. Cartridges were transferred into a 3D-printed dark box for dark incubation. (4) On-Tip Digestion: Adjust the pH by adding 30µL of Dissolve A to the cartridges, then introduce 12µL of the Digest Mix. Transfer the cartridges in the 3D printed box and then incubate for 80 min at 37°C for 80 min in Inheco. (5) Peptide Desalting and Elution: Following incubation, return the cartridges to the Bravo. Add 60µL of Buffer Transfer, perform two washes with 60µL of Buffer Wash, and then use 60µL of the Elute buffer to achieve efficient peptide transfer, washing, and elution. (6) Drying Peptides: Transfer the elution plate to the CombiDancer and evaporate the eluted peptide solutions in the 96-well plate at 55°C for 42 minutes.

For a detailed overview of this workflow, please refer to the Supplementary Information. A single iteration of the workflow takes 4.25 hours, while two iterations take 5.5 hours. Additionally, each pair of iterations can be grouped together as a block and can be conducted indefinitely by replenishing reagents and labware.

Sample preparation of plasma samples

This 96-well format workflow for plasma analysis was developed according to the previously published plasma proteome profiling protocol [16]. It consists of sample lysis, reduced, alkylated, trypsin digestion, peptides purification (desalting), and preparing peptide solutions for LC-MS/MS analysis. In detail:

(1) Upload Samples, reagents, and labware (e.g., containers and tips) to Cytomat hotels under the inventory management of Momentum Software.

(2) Transfer samples, reagents, and labware to the liquid-handling station: After initiating the process, transfer the peeled sample and buffer plates and other labware to the Biomek i7 by F7 and Spinnaker robot

(3) Denature, lyse, reduce and alkylate samples: transfer 4 μL plasma to a new plate, make 1:25 dilution by adding lysis buffer (1% SDC, 40 mM CAA, 10 mM TCEP, 100 mM Tris-HCl, at pH = 8.8), mix thoroughly by pipetting 3 times (30 μL/time) up and down, transfer the plate from Biomek i7 to ATC and heat the plate at 95°C for 5 min at ATC, and then transfer the plate to Covaris for 5 min sonication with 10 s on and 20 s off.

(4) Trypsin digestion: Centrifuge the plate at 2000 rpm for 2 min in Rotanta, transfer Xpeel peeled plate to Biomek i7, transfer 20 μL of the diluted plasma into a new plate, add 80 μL of 100 mM Tris-HCl (pH 8.8), add 2 μg of trypsin, mix thoroughly by pipetting 3 times up and down, and transfer the digestion plate to Inheco for 2h digestion at 37 °C.

(5) Peptide desalting: Centrifuge the plate at 2000 rpm for 1 min in Rotanta, transfer the peeled digestion plate to Bravo, add 15 μL of 10% Formic acid to stop the digestion, seal the plate by ALPS3000, and centrifuge at 6500 rpm for 7min, load the supernatant to pre-equilibrated Agilent C18 cartridges and purify peptides via Bravo peptide desalting program with 1% formic acid water and 80% Acetonitrile in 1% formic acid water.

(6) Prepare peptide solutions for LC-MS/MS analysis: concentrate the eluted peptides in CombiDancer at 55 °C for 42 min, re-suspend the peptide mixture in 30 μL of 0.1% formic acid water, measure the peptide concentration by Pierce Quantitative Colorimetric Peptide Assay kit in Multiskan SkyHigh, adjust peptides to a concentration of 500 ng/μL, transfer the peptide plate either to Vanquish loader for anlysis of Cytomat 2 Hotel for waiting analysis.

For a comprehensive overview of this workflow, please refer to the Supplementary Information. The duration for a single complete iteration of the workflow is 7.5 hours. Each additional iteration requires an incremental time of 1.5 hours. Moreover, this workflow can be sustained indefinitely through the replenishment of necessary reagents

and labware.

Sample preparation of cell line samples

The cell line sample analysis pipeline was developed based on the in-solution digestion method with sodium deoxycholate (SDC). Similar to the plasma pipeline, peptide desalting was performed on the Bravo platform, while other liquid-handling steps were executed using the Biomek i7. This pipeline can flexibly process samples with 500 ng to 200 μg of proteins by simply adjusting several volumes. In this manuscript, we analyzed proteins ranging from 500 ng to 20 μg to demonstrate sensitivity, using 20 μg of protein as the standard input for all cell line analyses. Cells were incubated in SDC lysis buffer (1% SDC, 100 mM Tris-HCl, pH 8.5) at 95 °C for 10 min and sonicated using a Qsonica Q800R3 sonicator for 10 min (85% power, 10 s on, 10 s off) to shear DNA and RNA. Protein concentrations were measured using a BCA assay. Then, TCEP (final concentration of 10 mM) and CAA (final concentration of 40 mM) were introduced, and the mixture was incubated for 10 minutes at 65 °C for reduction and alkylation. The automated pipeline includes the following steps:

(1) Upload Samples, reagents, and labware (e.g., containers and tips) to Cytomat hotels under the inventory management of Momentum Software.

(2) Transfer samples, reagents, and labware to the liquid-handling station: After initiating the process, transfer the peeled sample and buffer plates and other labware to Biomek i7 by F7 and Spinnaker robot

(3) Preparation for digestion: Adjust the volume of lysates to 50μL by adding 100 mM Tris-HCl (pH 8.8), mix thoroughly by pipetting 3 times up and down. For a protein input of 20 μg, add 30 μL of 100 mM Tris-HCl (pH 8.8) to samples that have a protein concentration of 1 μg/μL.

(4) Trypsin digestion: add trypsin to samples at an enzyme-to-protein ratio of 20:1, mix

thoroughly by pipetting 3 times up and down, and transfer the digestion plate to Inheco for 2h digestion at 37 °C.

(5) Peptide desalting: Centrifuge the plate at 2000 rpm for 2 min in Rotanta, transfer the peeled digestion plate to Bravo, add 50 µL of 100 mM Tris-HCl (pH 8.8) and 15 µL of 10% Formic acid to stop the digestion, seal the plate by ALPS3000, and centrifuge at 6500 rpm for 7min, load the supernatant to pre-equilibrated Agilent C18 cartridges and purify peptides via Bravo peptide desalting program with 1% formic acid water and 80% Acetonitrile in 1% formic acid water.

(6) Prepare peptide solutions for LC-MS/MS analysis: concentrate the eluted peptides in CombiDancer at 55 °C for 42 min, re-suspend the peptide mixture in 0.1% formic acid in water by Biomek i7 (for samples with 500ng, 1µg and 2µg proteins, add 8 µL; for samples with over 5µg proteins, add 15 µL), measure the peptide concentration by Pierce Quantitative Colorimetric Peptide Assay kit in Multiskan SkyHigh (optional), adjust peptides to a concentration of 500 ng/µL(optional), transfer the peptide plate either to Vanquish loader for anlysis of Cytomat 2 Hotel for waiting analysis.

For a comprehensive overview of this workflow, please refer to the Supplementary Information. The duration for a complete iteration of the workflow is 7.5 hours, and each additional iteration requires an extra 1.5 hours. Moreover, this workflow can be sustained indefinitely by replenishing the necessary reagents and labware. It is also capable of processing lysates from various types of samples, including tissues.

LC-MS analysis of LCM samples

Samples were measured with a timsTOF pro mass spectrometer (Bruker Daltonics) coupled to an UltiMate 3000 RSLCnano liquid chromatography system (Thermo Fisher Scientific). Dissolved peptides were loaded to a 20 cm analytical column (100 µm i.d., packed with 1.9 µm C18 particles) and separated at a flow rate of 300 nl/min using a

60-min gradient from 5% to 32% buffer B (acetonitrile with 0.1% formic acid). The mass spectrometer was operated in data-dependent acquisition (DIA) mode with 8 dia-PASEF scans separated into 4 ion mobility windows per scan covering an m/z range from 400 to 1040 by 20 Th windows and an ion mobility range from 0.75 to 1.3 V s cm−2. The capillary voltage was set to 1,750 V. The accumulation and ramp time were specified at 166 ms. The collision energy was decreased from 59 Vs at $1/K_0 = 1.6$ V cm^{-2} to 20 eV at $1/K_0 = 0.6$ Vs cm^{-2}.

LC-MS analysis of plasma samples

Samples were analyzed using an Orbitrap Exploris 480 mass spectrometer (Thermo Fisher Scientific) coupled with a Vanquish Neo UHPLC system (Thermo Fisher Scientific). Vanquish UHPLC Loader and Xcalibur sequence were configured in Momentum Workflow Scheduling Software to enable automated LC-MS analysis. Samples were analyzed by 48SPD DIA methods. Peptides were transferred to a 15 cm Kinetex C18 column (300 µm i.d., packed with 2.6 µm C18 particles, Phenomenex) and separated at a flow rate of 9 µL/min using a 26-min gradient from 6% to 40% buffer B (80% acetonitrile with 0.1% formic acid). The mass spectrometer was operated in data-dependent acquisition (DIA) mode. Full-MS scans were acquired at 60,000 resolution from 350 to 1000 m/z with a normalized automatic gain control (AGC) target of 300% and a maximum injection time of 45 ms. 19 variable windows with 1 Da overlap were used. The resolution was set to 30,000 at 200 m/z. The normalized AGC target was set at 1900%, and the maximum injection time was at 54 ms. Normalized HCD energy was set to 28%.

LC-MS analysis of cell line samples

Different input experiments and all ten cell line samples were analyzed using the high-throughput workflow. The automated LC-MS/MS data acquisition was initiated by the Momentum Workflow Scheduling Software with a pre-defined Xcalibur sequence file.

Samples were analyzed using a 24 SPD DIA method on Orbitrap Exploris 480 mass spectrometers (Thermo Fisher Scientific) coupled with a Vanquish Neo UHPLC system (Thermo Fisher Scientific). Peptide separations were carried out on a 15 cm Kinetex C18 column (300 µm i.d., packed with 2.6 µm C18 particles, Phenomenex) with a 52-min gradient from 6% to 40% buffer B (80% acetonitrile with 0.1% formic acid) at a flow rate of 9 µL/min. For the full MS experiment, one scan acquired over m/z 400–1003 at a resolution of 120,000 with a normalized AGC target value at 300% and an auto maximum injection time. For the MS/MS experiment, a total of 90 scans (30 scans/cycle) were acquired at a resolution at 30,000 with a normalized AGC target value at 1900% and with auto maximum injection time. Precursor ions were fragmented by HCD with normalized collision energy at 26%.

The different input experiments were also analyzed using the sensitive workflow. Measurements were performed on a timsTOF Pro mass spectrometer (Bruker Daltonics) coupled to an UltiMate 3000 RSLCnano system (Thermo Fisher Scientific). Peptides were separated on a 20-cm analytical C18 column (100 µm i.d., 1.9 µm particles) at 300 nL/min using a 60-min gradient from 6% to 40% of buffer B (acetonitrile with 0.1% formic acid). The mass spectrometer was operated in DIA mode using a dia-PASEF method consisting of 16 ramps, each subdivided into 4 isolation windows, resulting in a total of 64 DIA windows per cycle. The acquisition covered an m/z range of 300–1500 with 20 Th windows and an ion mobility range of 0.75–1.4 Vs cm−2. The capillary voltage was set to 1,750 V, with accumulation and ramp times of 100 ms. Collision energy decreased from 59 Vs at 1/K0 = 1.6 V cm−2 to 20 eV at 1/K0 = 0.6 Vs cm−2.

Development and setup of π-ProteomicInfo

We developed an automated system for data storage, processing, quality control, and LC-MS status monitoring. The system consists of a monitor, storage, processing, analysis, and feedback module. We refer to this system as π-ProteomicInfo. For raw data storage, we used a commercial intelligent content management platform called

Anyshare (AISHU Technology Corp). This platform monitors the raw data file on the LC-MS computers and automatically transfers the data to storage servers as soon as it is generated. Anyshare servers are also storage servers for our processed data and files. The data processing, quality control, analysis, and feedback tools were developed in-house using Python and integrated into three user interfaces: SearchClient, SearchServer, and SendInfo. The scripts can be accessed through GitHub (https://github.com/PHOENIXcenter/pi-ProteomicInfo).

SearchClient must be installed on the LC-MS computers. It monitors the LC-MS data generation and sends raw data to computers and servers with proteomic software. It also tracks the instruments' status and sends the information to the computer installed SendInfo.exe. SearchServer must be installed on computers that run proteomic software such as MaxQuant[17], Proteome Discoverer, or Spectronaut. Once raw data is received in a predefined location, SearchServer will trigger protein identification and quantification in these software applications. It will also extract quality control (QC) information, which includes the number of MS1 and MS2 spectra, peptides, and proteins, as well as details such as cycle time, peak capacity, full width at half maximum, retention time, and data points. This information will be summarized in a text file named "stat_summary.txt," and the text file will be sent to the computer with the installed SendInfo.exe. SendInfo can be installed on a data processing computer or a separate one. It sends messages about QC and LC-MS status to specialists on WeChat or by email to keep them informed.

For processing data from a large cohort, we prefer using the Linux version of DIA-NN. The automation of protein quantification is accomplished through SearchClient and the command line. SearchClient monitors the generation of DIA raw data and sends this data to the server where DIA-NN is installed. Once an individual run is received, DIA-NN begins processing the data using pre-defined parameters and generates a .quant file. The raw files generated by Thermo Scientific mass spectrometers are first converted to .mzML format automatically before being processed by DIA-NN. After processing

individual runs, when the number of .quant files reaches the total sample number of the cohort, DIA-NN aggregates the information from all the .quant files, performs cross-run steps, and calculates the final quantities. Using SearchClient and SearchServer can also achieve large numbers of DIA data analyses; it is an optional method. However, as Spectronaut is time-consuming and prone to crashing, we usually don't use it for large-scale analysis without request.

Data processing by π-ProteomicInfo

We use Proteome Discoverer (v2.5) for daily DDA QC of Thermo Scientific LC-MS instruments, MaxQuant (v2.0.3) for Bruker LC-MS instruments, and Spectronaut (v15.7) for daily DIA QC. We implemented all these proteomic software in π-ProteomicInfo. To set everything up, we need to specify a few key locations. First, provide the file location of data acquisition (Acquisition dir) and the temporary file location for the file transfer (Data storage) on SearchClient. Next, on SearchServer, select the type and version of software, the file location for the received directory (Received.dir), and the parameter file. The automated data processing is ready and processes new data once it is generated.

All the results shown in this study are generated by DIA-NN (v 1.8.1)[18], as it is much faster. For DIA-NN, we only need to choose the file location of data acquisition (Acquisition dir) and the temporary file location for the file transfer (Data storage) on SearchClient. Once the data is received by the server with DIA-NN, the process is initiated by the command line. The analyses were then conducted in a library-free mode with some modifications: methionine oxidation and protein N-terminal acetylation were set as the variable modifications, the maximum number of variable modifications was limited to five, the MBR feature was activated, and cross-run normalization was disabled.

Data analysis

Data analysis was conducted using R software (version 4.0.3) with the output from DIA-NN in the report titled "pg_matrix.tsv." In counting the number of peptide or protein identifications for each sample, only those peptides or proteins with valid intensities greater than zero were included. For quantitative analysis, the data underwent log10 transformation and were normalized through median centering. Proteins that exhibited more than 50% missing values in each group were filtered out. The remaining missing values were addressed using the K-nearest neighbors (KNN) imputation method. Principal Component Analysis (PCA) was carried out on proteins quantified across all experiments involving different cell lines, utilizing the “prcomp” function in R. Gene set enrichment analysis was performed using hallmark gene sets from the Molecular Signature Database (MSigDB) [19] with the clusterProfiler package (version 4.10.1)[20]. Figures were created with the ggplot2 and pheatmap packages. We also integrated fundamental data analysis in SearchServer. It enables automated extraction of QC information and generation of plots from quantitative files outputted by MaxQuant, Proteome Discover, and Spectronaut. We mainly use this function of AutoSearch to evaluate the QC data and a single run of cohort samples.

Supplementary figures

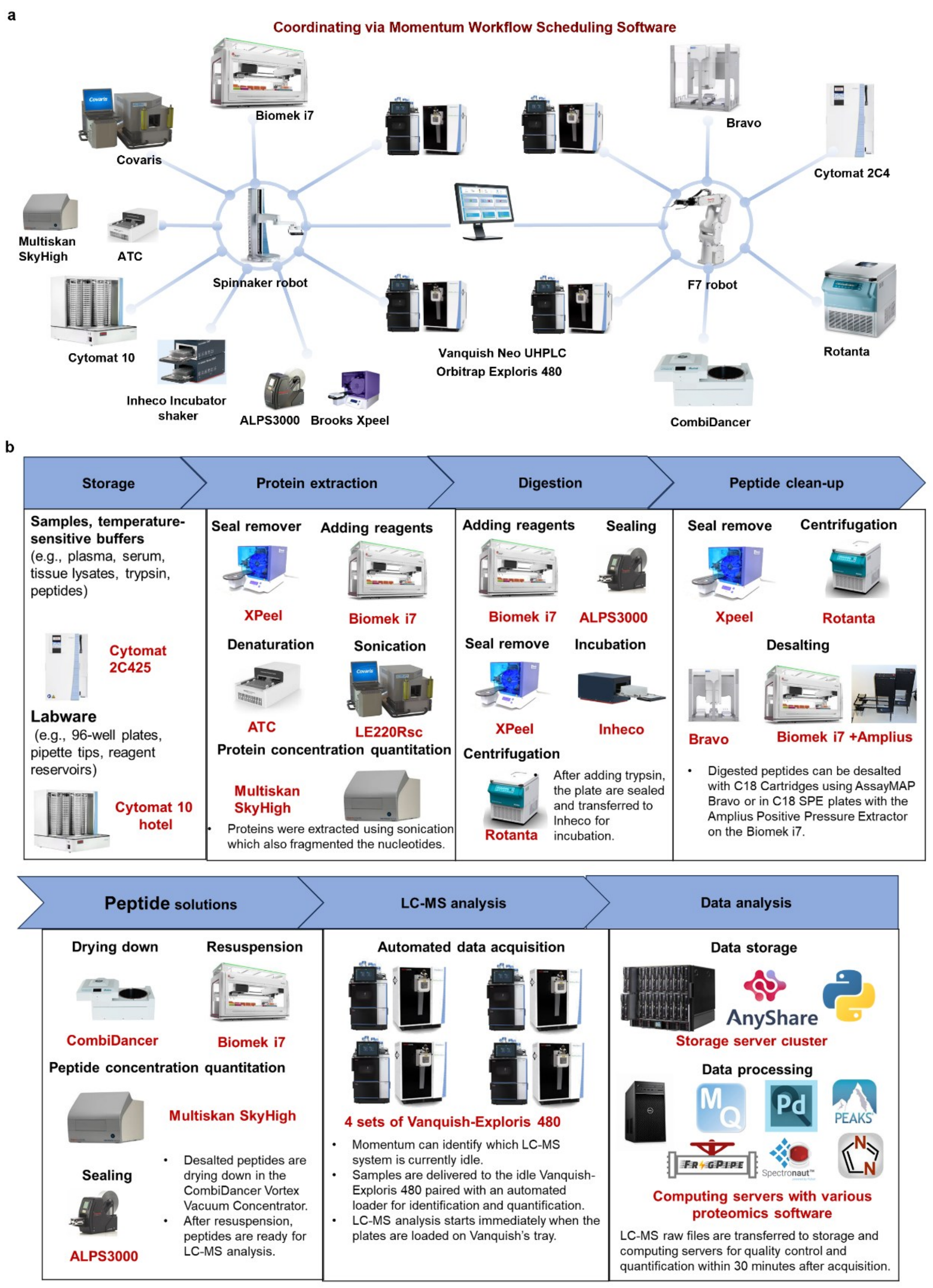

Supplementary Fig. S1 Devices and software of π-HuB data factory. a, Customized devices that form the linear, expandable, and connectable π-Station. All these devices are interconnected through the Spinnaker or F7 robot, managed by Momentum software.

b, Diagram of a typical proteomic analysis workflow, highlighting the devices and software involved at each step of the process.

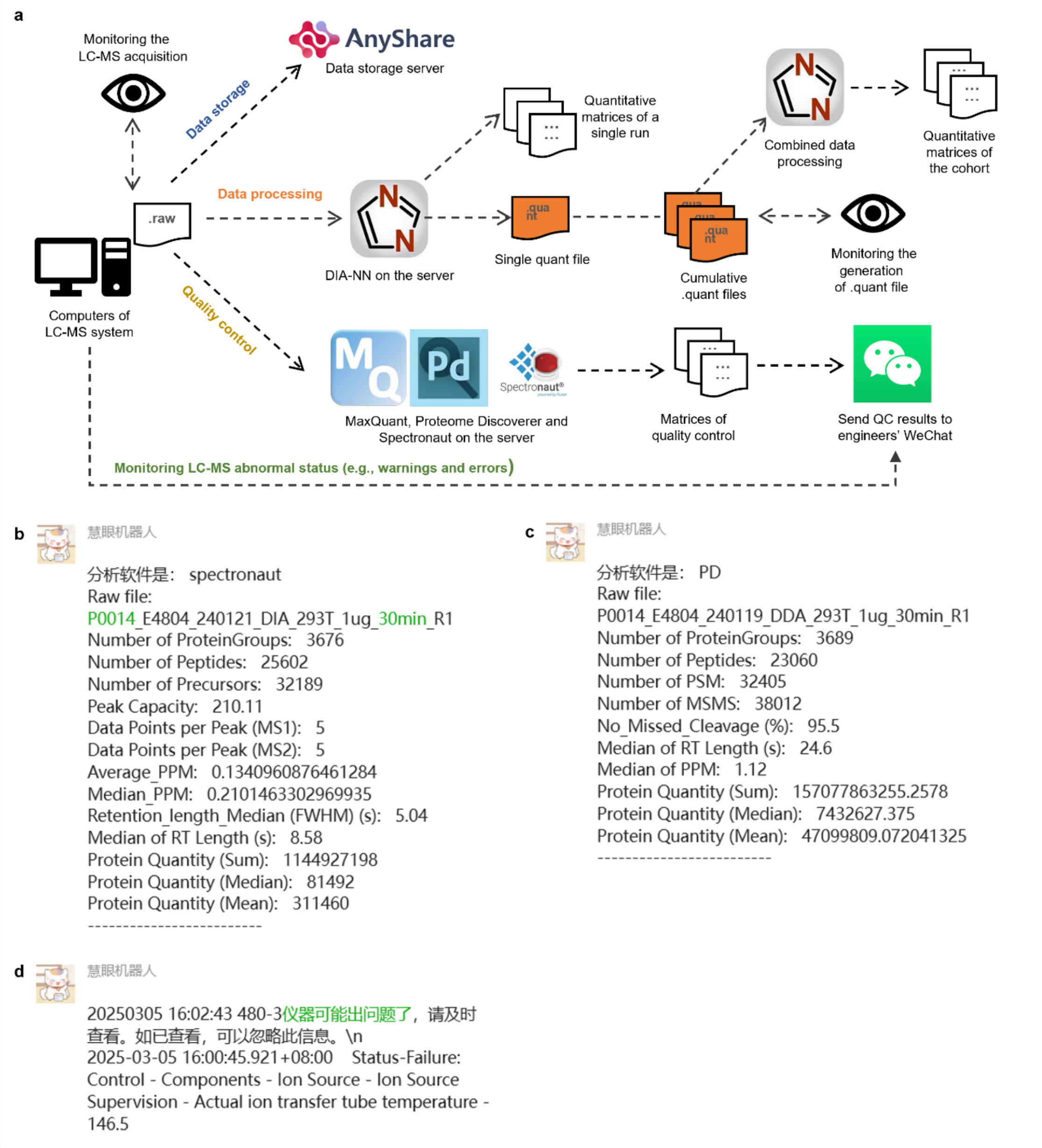

Supplementary Fig. S2 Illustration of the π-ProteomicInfo system. a, The workflows involved in data storage, data processing, quality control, monitoring, and feedback within π-ProteomicInfo. **b,** An example of DIA quality control that π-ProteomicInfo extracted and sent to a specialist's social media application. **c,** An example of DDA quality control that π-ProteomicInfo extracted and sent to the specialist's social media application. **d,** An example of an abnormal status detected in an LC-MS/MS that π-ProteomicInfo detected and sent to a specialist's social media application to prompt timely checks and maintenance.

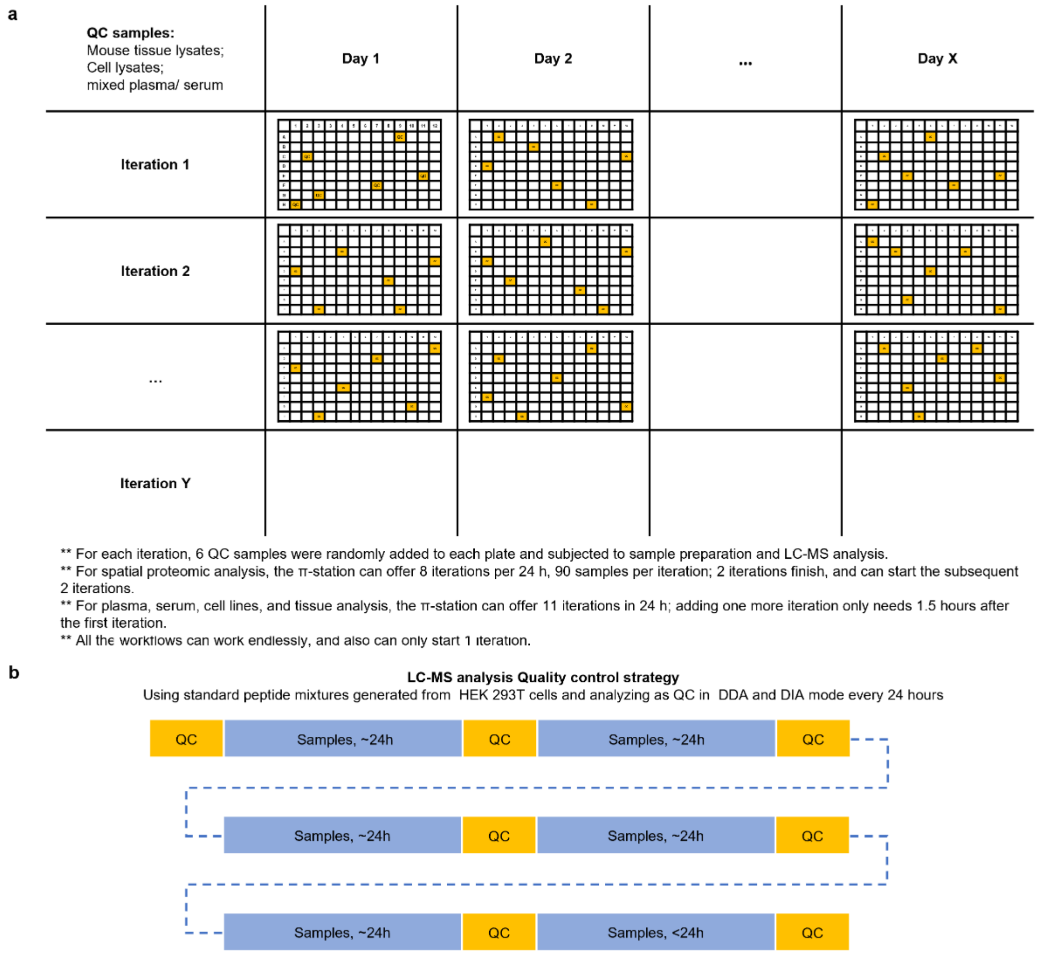

Supplementary Fig. S3 Illustration of the quality control strategies at the π-Hub data factory. a, Quality control strategy for sample preparation at π-Station. **b,** Quality control strategy for LC-MS/MS data acquisition.

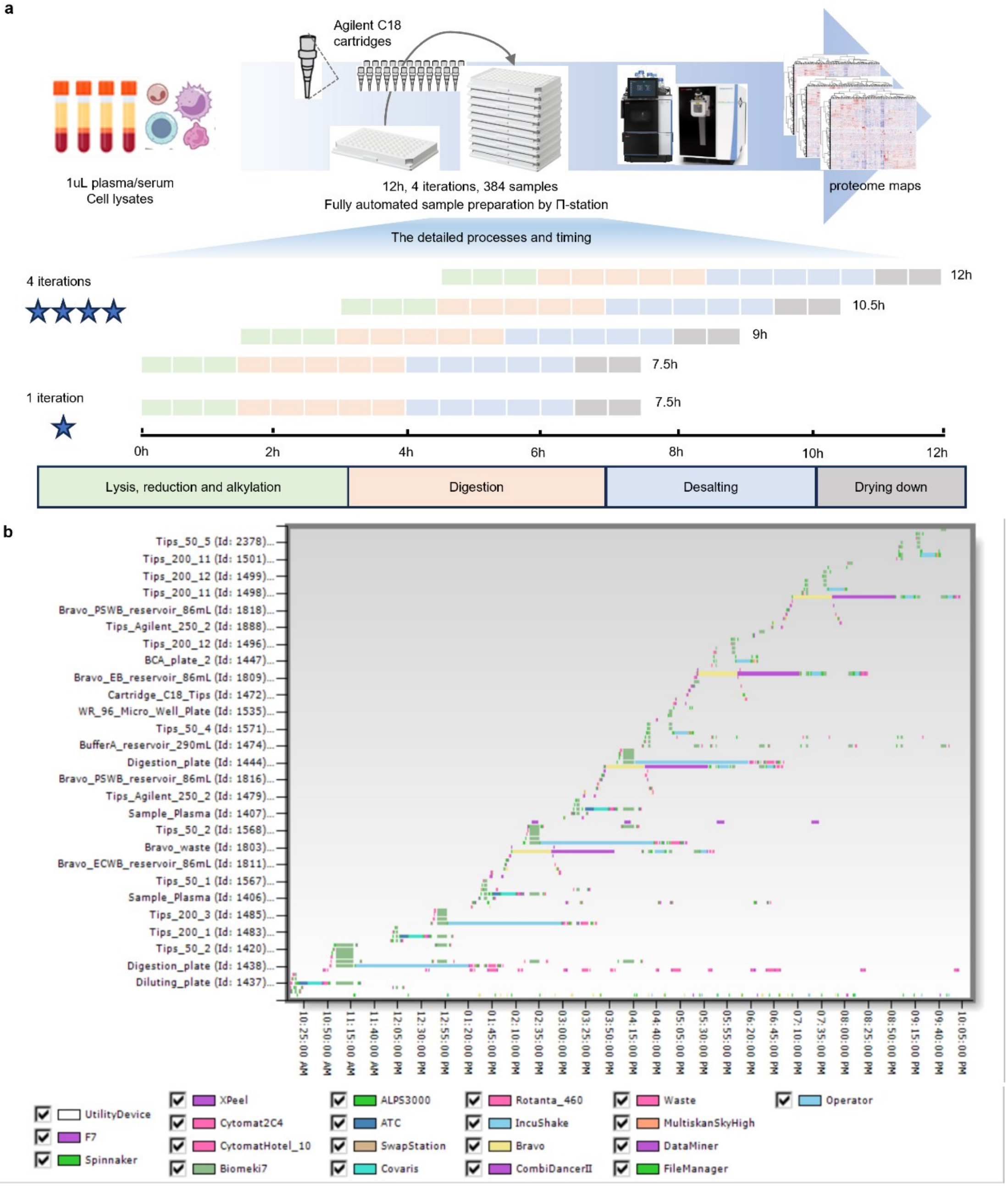

Supplementary Fig. S4 Workflows for the plasma and cell line proteome analysis at the π-Hub data factory. a, Schematic of the workflows for analyzing plasma and cell line proteome. **b,** Gantt chart of plasma proteome workflow. Since the workflows for these two sample types are very similar, we have not provided a separate chart for the cell line samples. Detailed descriptions of both workflows can be found in the Methods section.

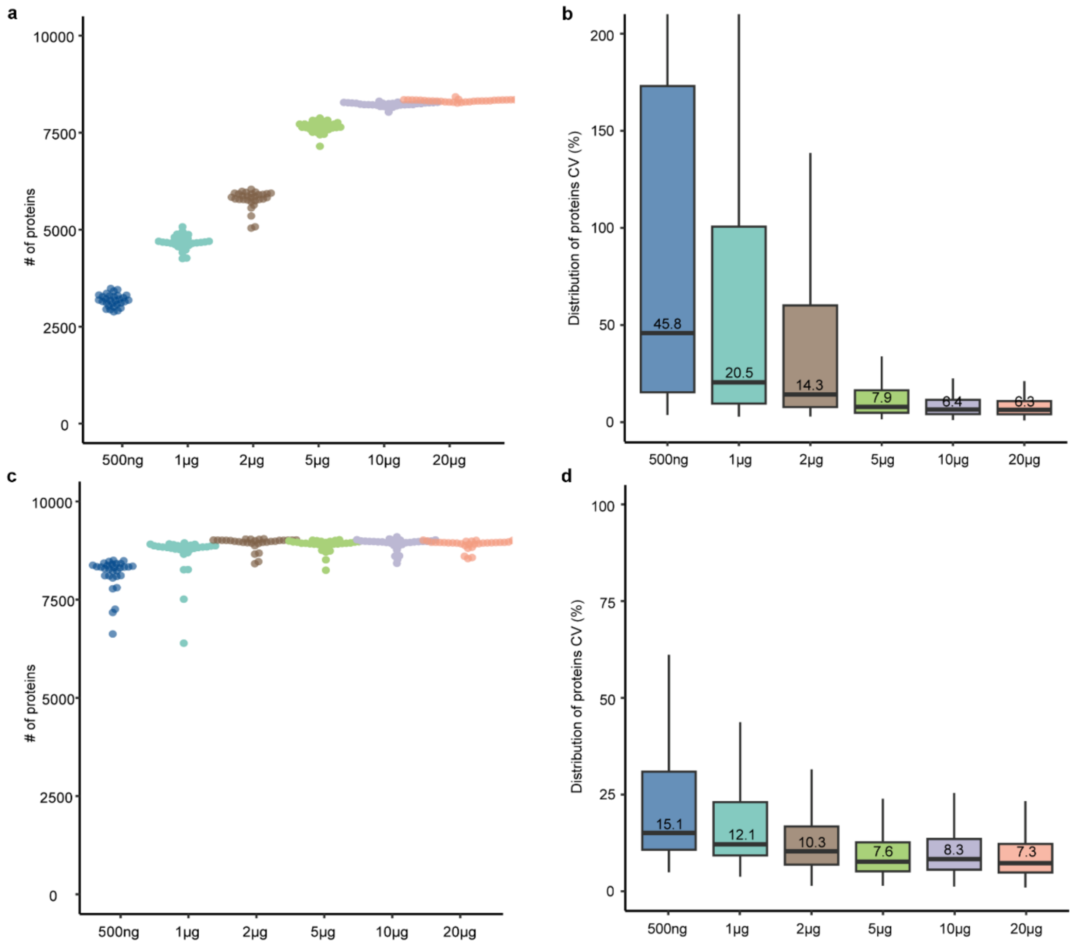

Supplementary Fig. S5 Performance of the π-Hub data factory using different amounts of 293T protein as inputs. a, The number of proteins quantified from 500 ng to 20 µg starting amount of protein by the high-throughput LC-MS/MS method. **b,** The distribution of CVs of proteins detected by the high-throughput LC-MS/MS method. **c,** The number of proteins quantified from 500 ng to 20 µg starting amount of protein by the sensitive LC-MS/MS method. **d,** The distribution of CVs of proteins detected by the sensitive LC-MS/MS method.

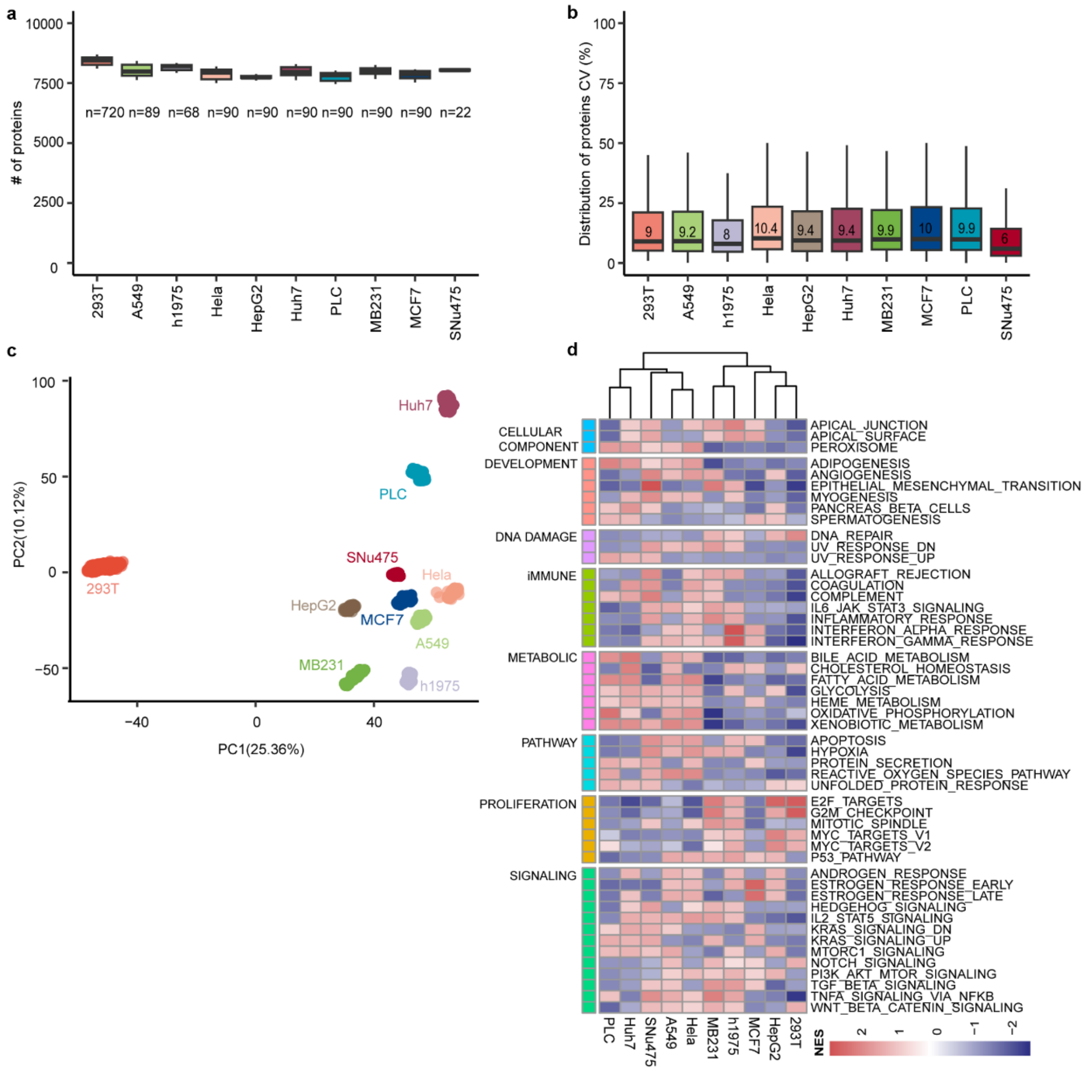

Supplementary Fig. S6 Performance of the π-Hub data factory for analyzing different types of cells. a, The number of proteins quantified in each cell line. The number of replicates ranges from 22 to 720. **b,** The distribution of CVs of proteins detected in each cell line. **c,** The principal component analysis of these 10 cell lines. **d,** Single-sample gene set enrichment analysis identifying the biological features of these cell lines.

a

Date	Iterations of sample preparation	# of QC	# of proteins (average)	# of precursors (average)	Std of protein numbers	Std of precursor numbers	median CV of protein intensities
Day 1	Iteration_01	6	8379	151803	96	5022	6.49
Day 2	Iteration_02	6	8374	151802	79	3789	6.37
Day 4	Iteration_03	6	8293	148726	181	6562	6.66
Day 5	Iteration_04	6	8365	151918	176	6836	6.36
Day 14	Iteration_05	6	8406	153263	159	6064	7
Day 15	Iteration_06	6	8417	153709	124	5917	6.87
Day 16	Iteration_07	6	8391	152390	154	5970	7.18
Day 21	Iteration_08	6	8334	150850	44	1726	5.29
	Iteration_09	6	8251	147600	44	1469	4.72
	Iteration_10	6	8395	153874	62	1579	4.32
	Iteration_11	6	8507	158146	18	1614	4.94
Day 23	Iteration_12	6	8422	154210	151	5900	6.74
Day 25	Iteration_13	6	8430	153410	185	7758	6.95
Day 27	Iteration_14	6	8410	153332	161	8433	6.53
Day 32	Iteration_15	6	8374	152330	147	6022	7.05
Day 33	Iteration_16	6	8493	155311	67	2914	4.51
Day 48	Iteration_17	6	8348	152008	41	1960	7.59
Day 63	Iteration_18	6	8453	156242	34	1471	6.07
Total		108	8391	152829	126	5214	7.62

b

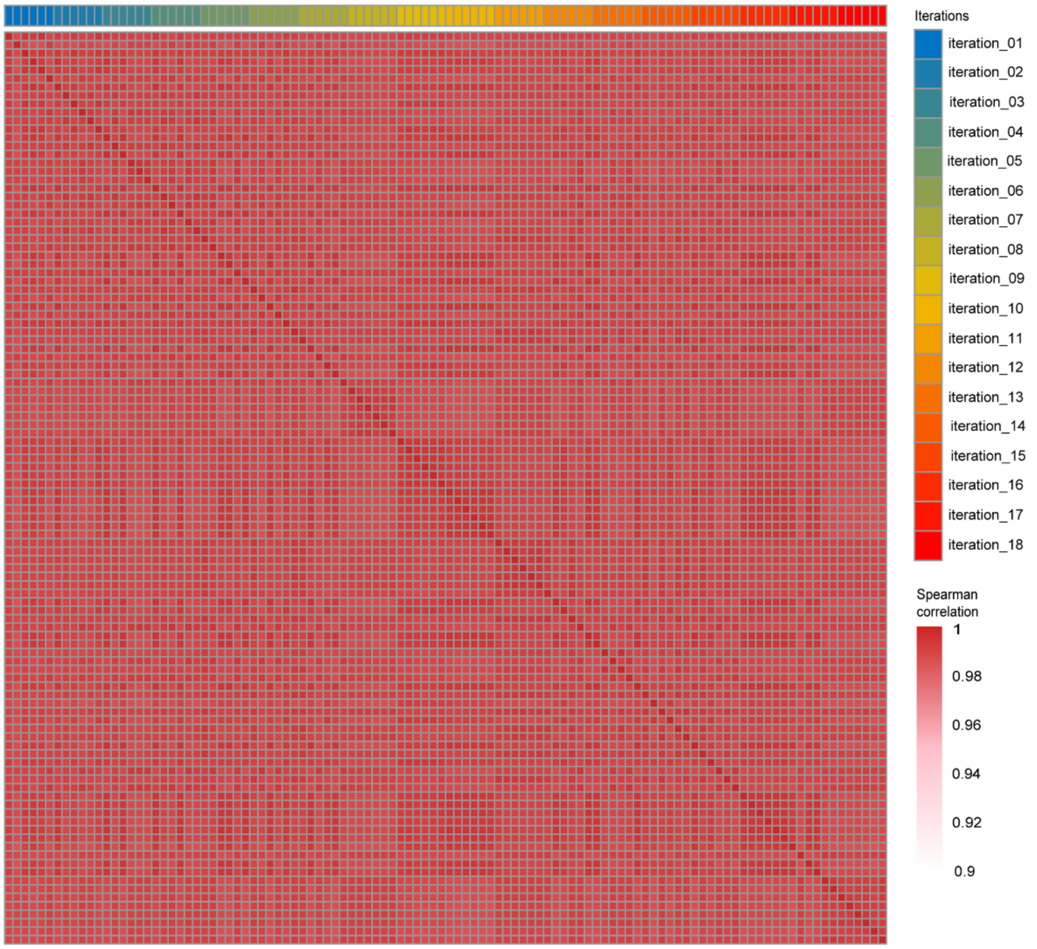

Supplementary Fig. S7 Long-term performance of the π-Hub data factory. a, Overview of the quality control data across 18 iterations over a 2-month period. It includes a summary of the number of proteins and precursors, as well as median CV values for individual days and across days. The variability observed is influenced by

both sample preparation and LC-MS/MS data acquisition. In each iteration, 6 QC samples were included and processed alongside the other 90 samples. The sample preparation and data acquisition both occurred over the span of 2 months. **b,** The Spearman correlation heatmap of these 108 QC samples.

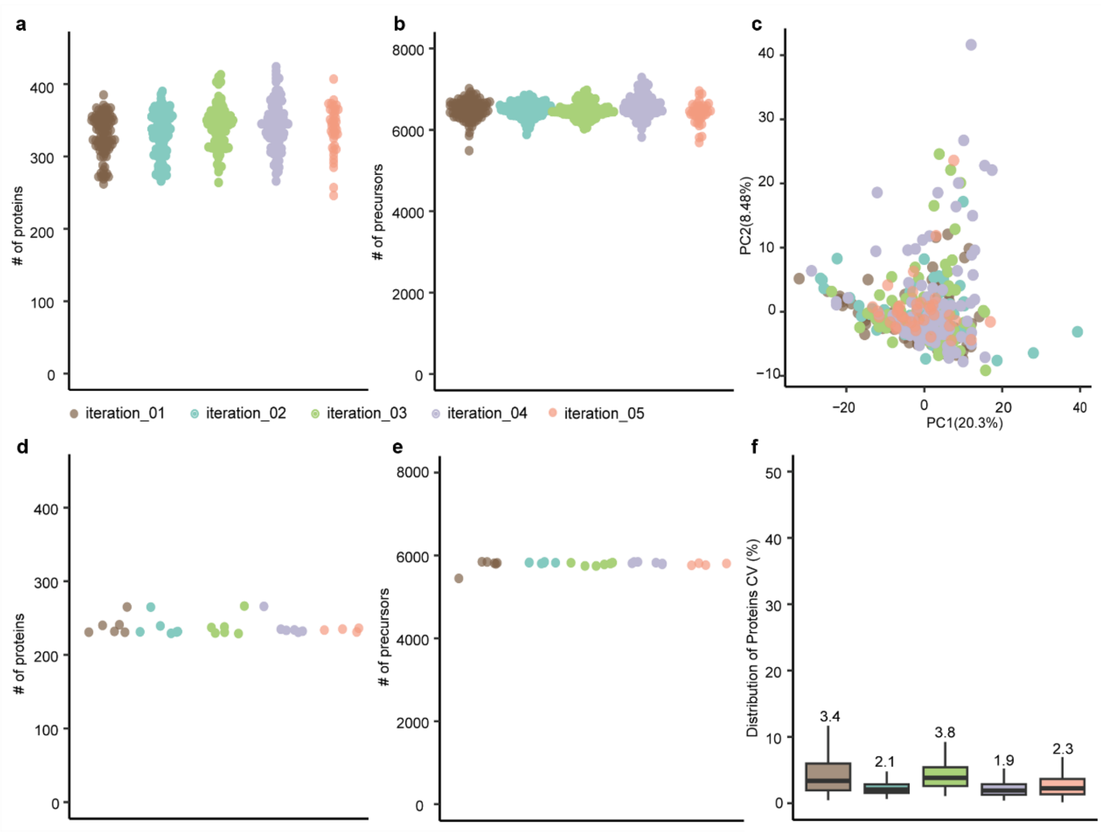

Supplementary Fig. S8 Performance of the π-Hub data factory for analyzing plasma. a, The number of proteins quantified in 398 plasma samples, which were processed in 5 iterations. **b,** The number of precursors detected in 398 plasma samples. **c,** The principal component analysis of samples processed in different iterations. **d,** The number of proteins quantified in QC samples. We used a pool of healthy human plasma as QC samples during plasma proteome analysis. **e,** The number of precursors detected in QC samples. **b,** The distribution of CVs for proteins detected in QC samples in each iteration.

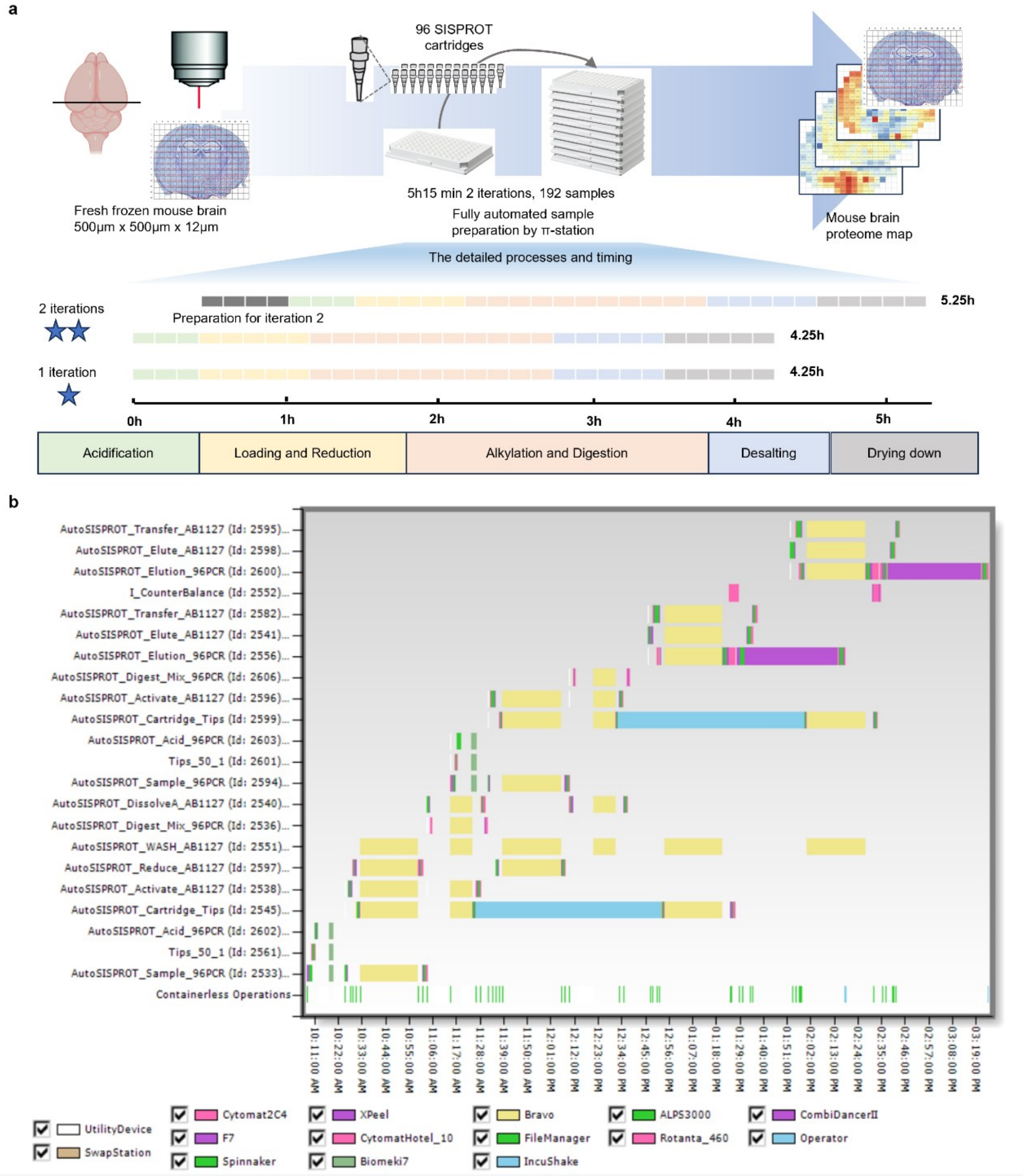

Supplementary Fig. S9 The workflow of spatial proteomic analysis at the π-HuB data factory. a, Schematic of the spatial proteomic workflow. **b,** Gantt chart for the spatial proteomic workflow that involves 2 iterations of analysis.

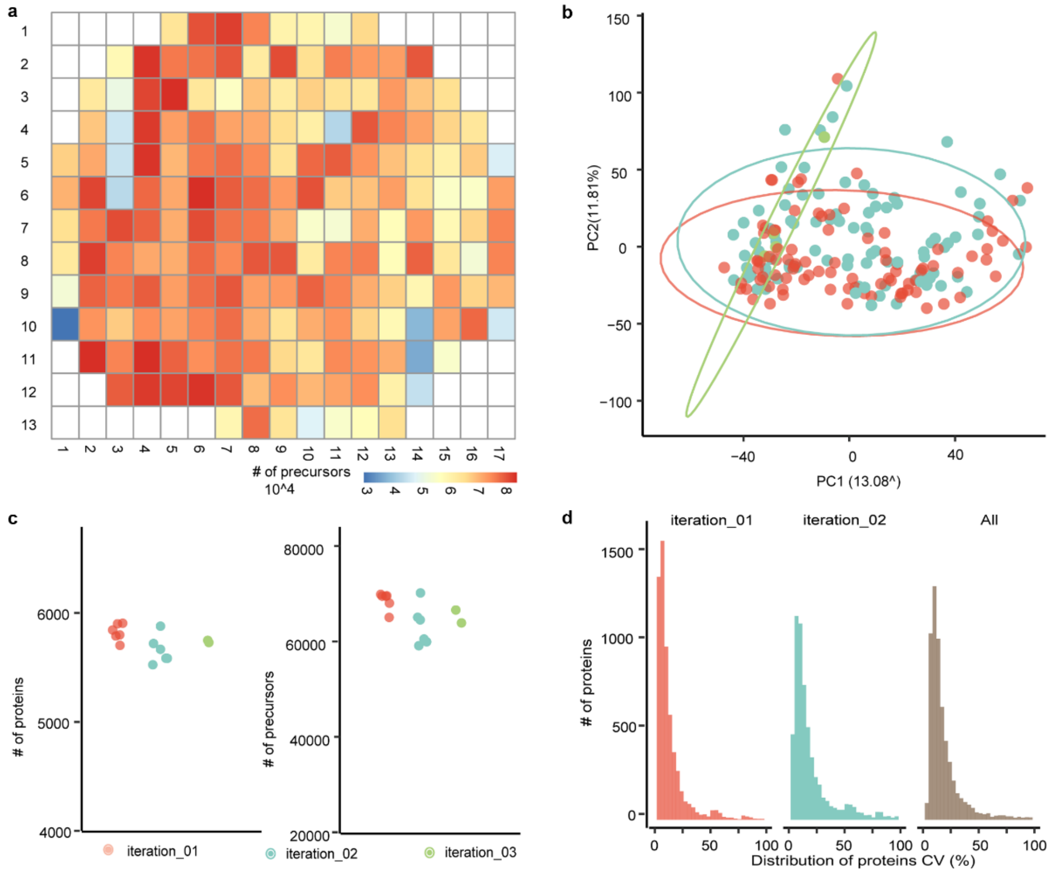

Supplementary Fig. S10 Performance of the spatial proteomic analysis at the π-Hub data factory. a, The number of precursors identified in each micro-specimen of the coronal section of the mouse brain. **b,** The principal component analysis (PCA) of samples analyzed across 3 iterations. **c,** The number of proteins and precursors identified in sample preparation quality control. 250 ng of mouse brain lysates were used as the quality control. **d,** The distribution of CVs of protein intensities in sample preparation quality control samples by iterations.

Momentum processes for generating proteome data

Contents

1. Illustration of the process for generating spatial proteome data

2. Illustration of the process for generating cell line proteome data

3. Illustration of the process for generating plasma proteome data

1. The process for generating spatial proteome data

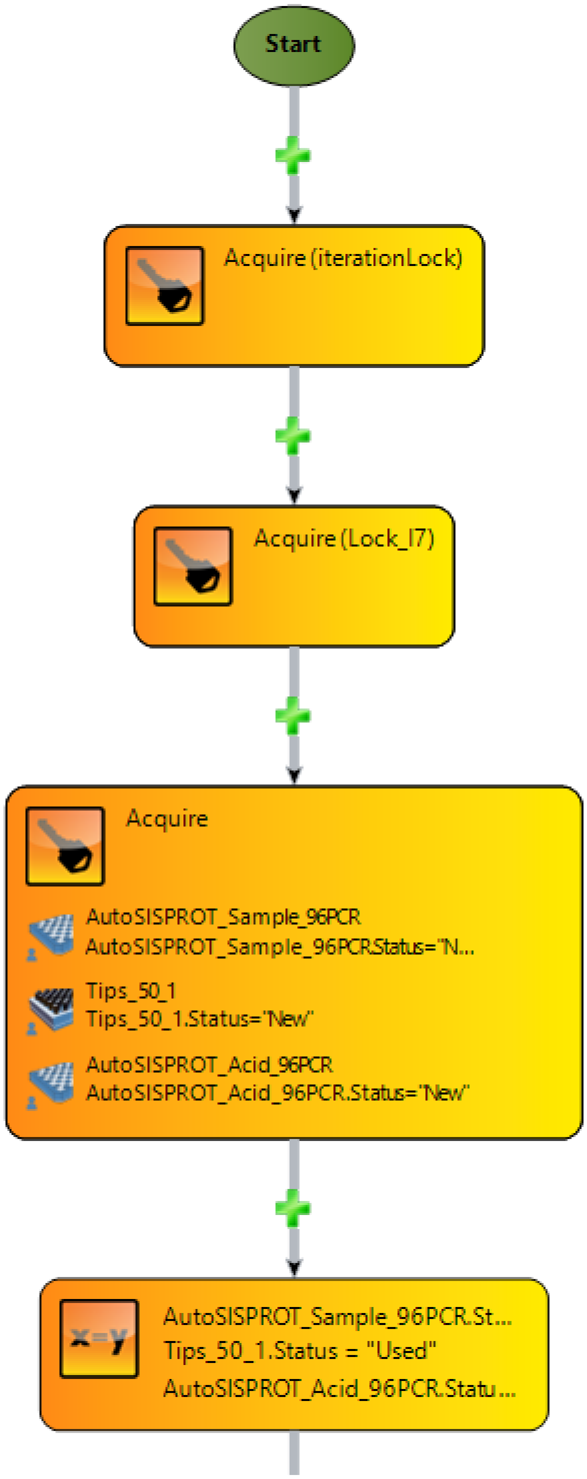

Acquire samples, reagents, and labware such as tips.

Set the status of labware.

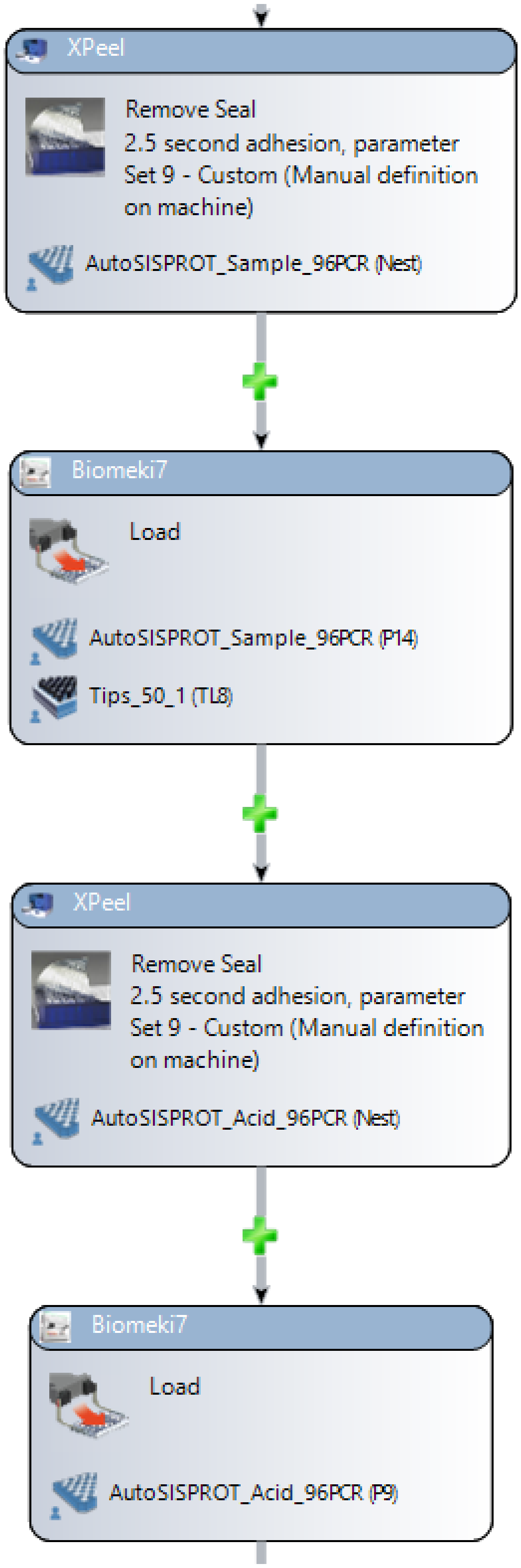

Peel off the film from the sample plate.

Load the sample plate and tip box to Biomek i7.

Peel off the film from the formic acid plate.

Load the formic acid container to Biomek i7.

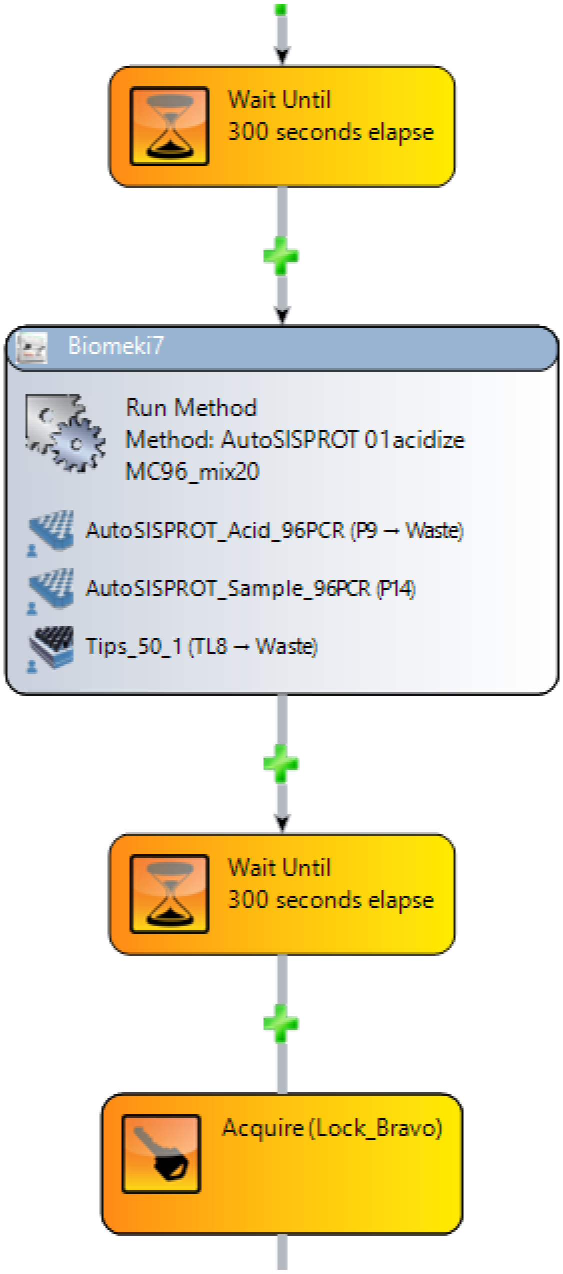

Run the sample acidification method on Biomek i7.

Acquire and lock Bravo for the following process.

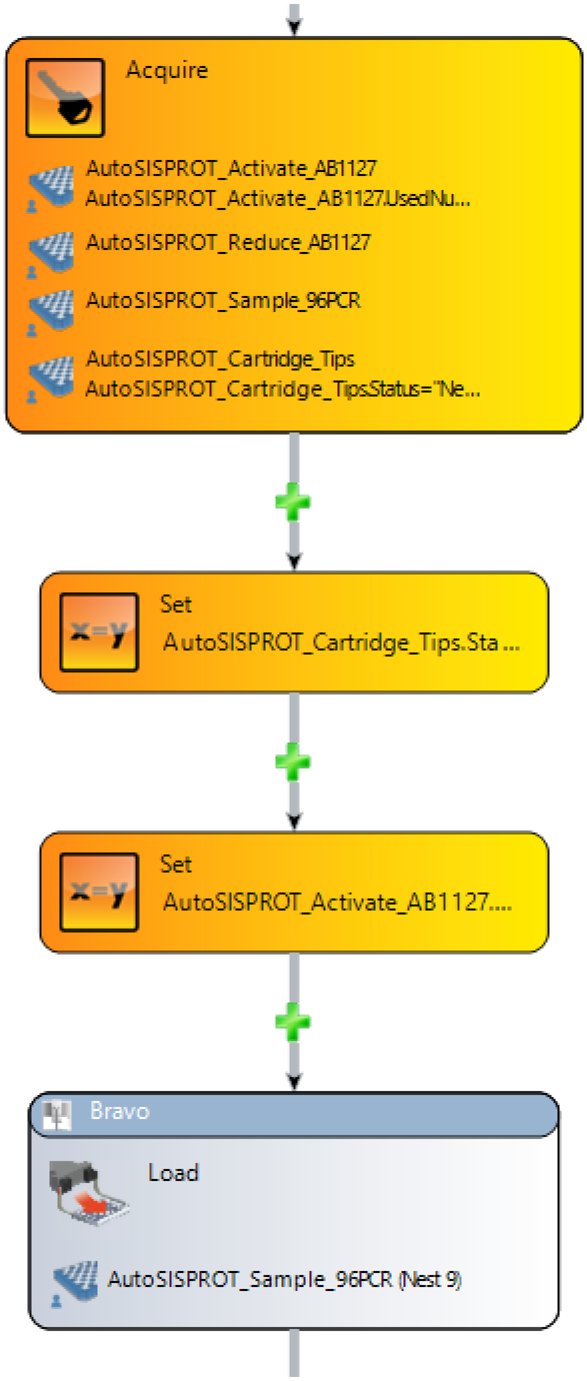

Acquire samples, reagents, and labware.

Set the status of SISPROT Cartridges.

Set the status of Buffer Activate.

Load the sample plate to Bravo.

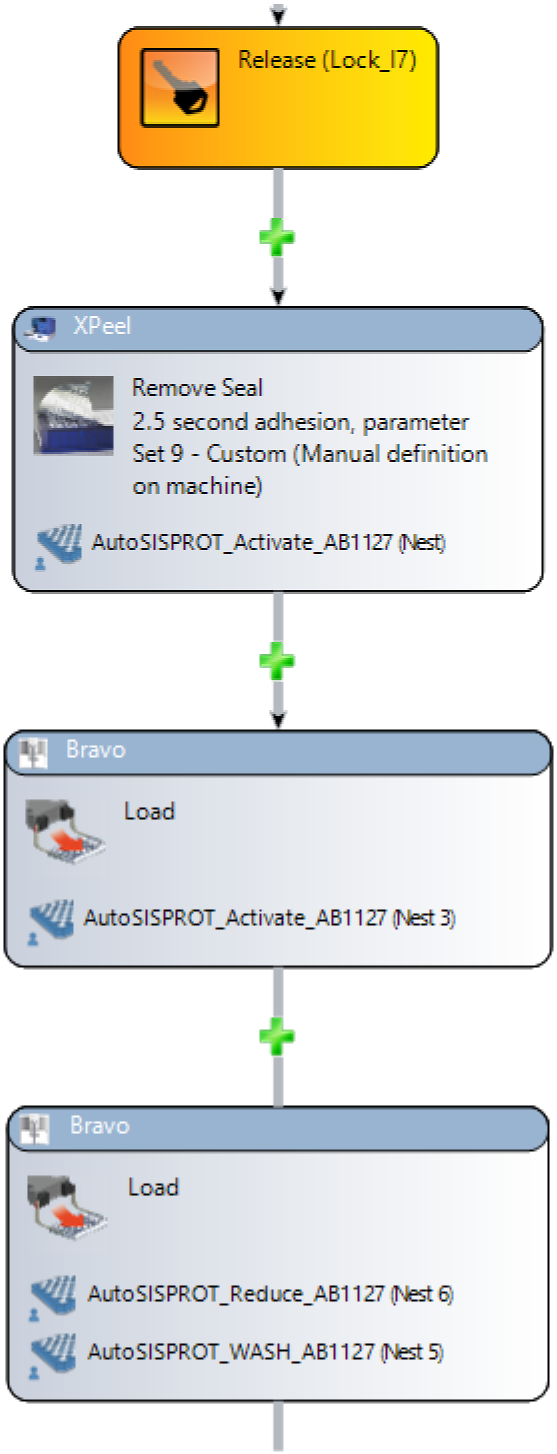

Release Biomek i7.

Peel off the film from Buffer Activate container.

Load the Buffer Activate container to Bravo.

Load the Buffer Reduce and Wash containers to Bravo.

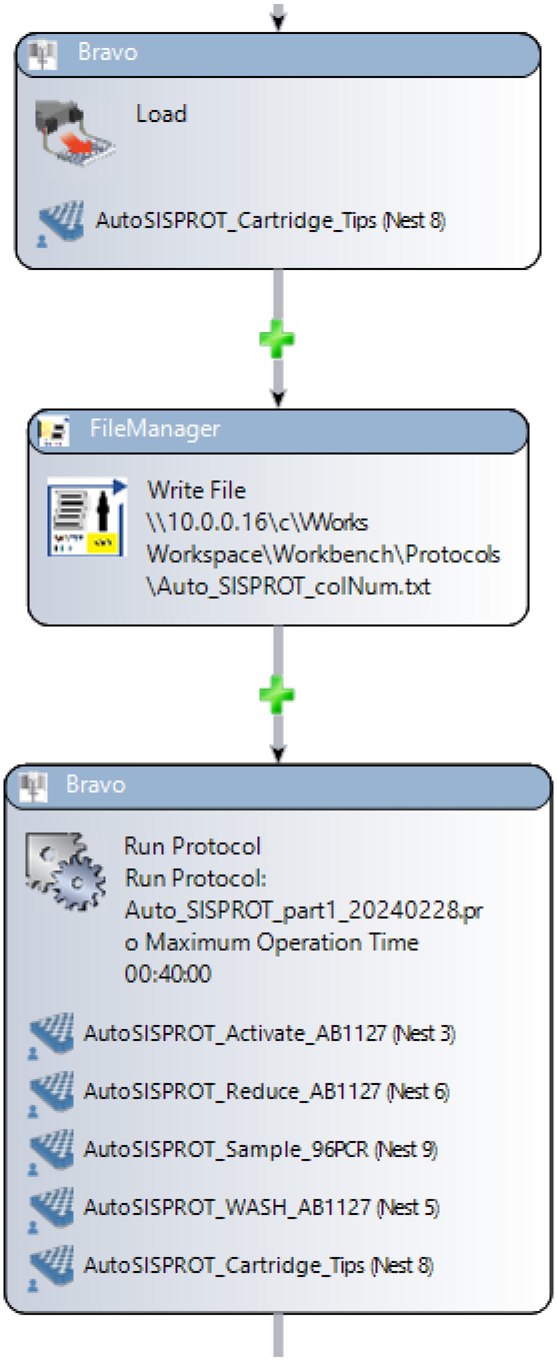

Load the SISPROT Cartridges to Bravo.

Read the column numbers of the sample plate with samples.

Run the Bravo protocol for activation and equilibration of SISPROT cartridges, sample loading, and reduction.

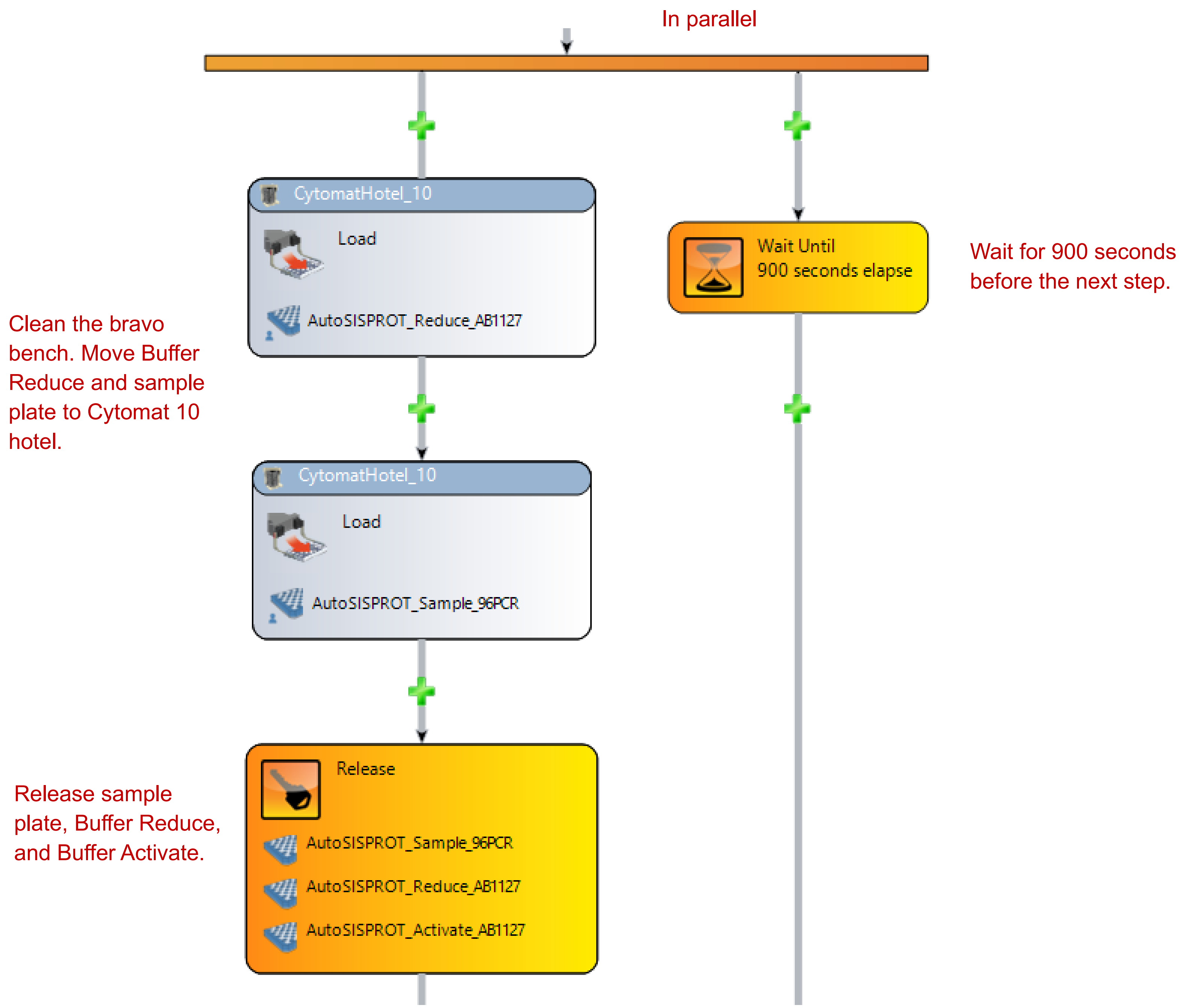
In parallel
CytomatHotel_10
Load
AutoSISPROT_Reduce_AB1127
Wait Until
900 seconds elapse
Wait for 900 seconds before the next step.
Clean the bravo bench. Move Buffer Reduce and sample plate to Cytomat 10 hotel.
CytomatHotel_10
Load
AutoSISPROT_Sample_96PCR
Release
AutoSISPROT_Sample_96PCR
AutoSISPROT_Reduce_AB1127
AutoSISPROT_Activate_AB1127
Release sample plate, Buffer Reduce, and Buffer Activate.

Acquire Buffer Dissolve A, DigestMix, and Activate.

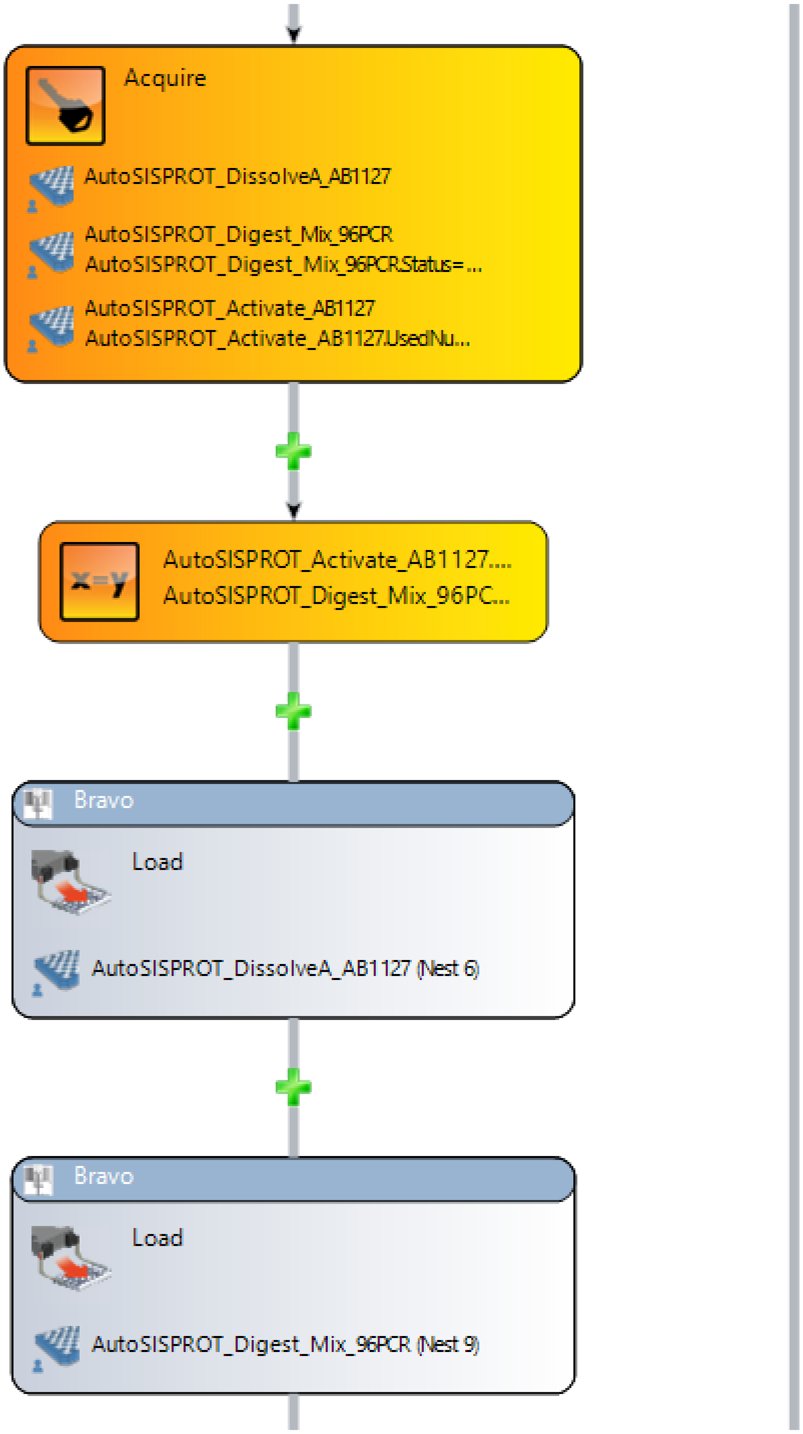

Load Buffer Dissolve A, Digest Mix to Bravo.

图 8

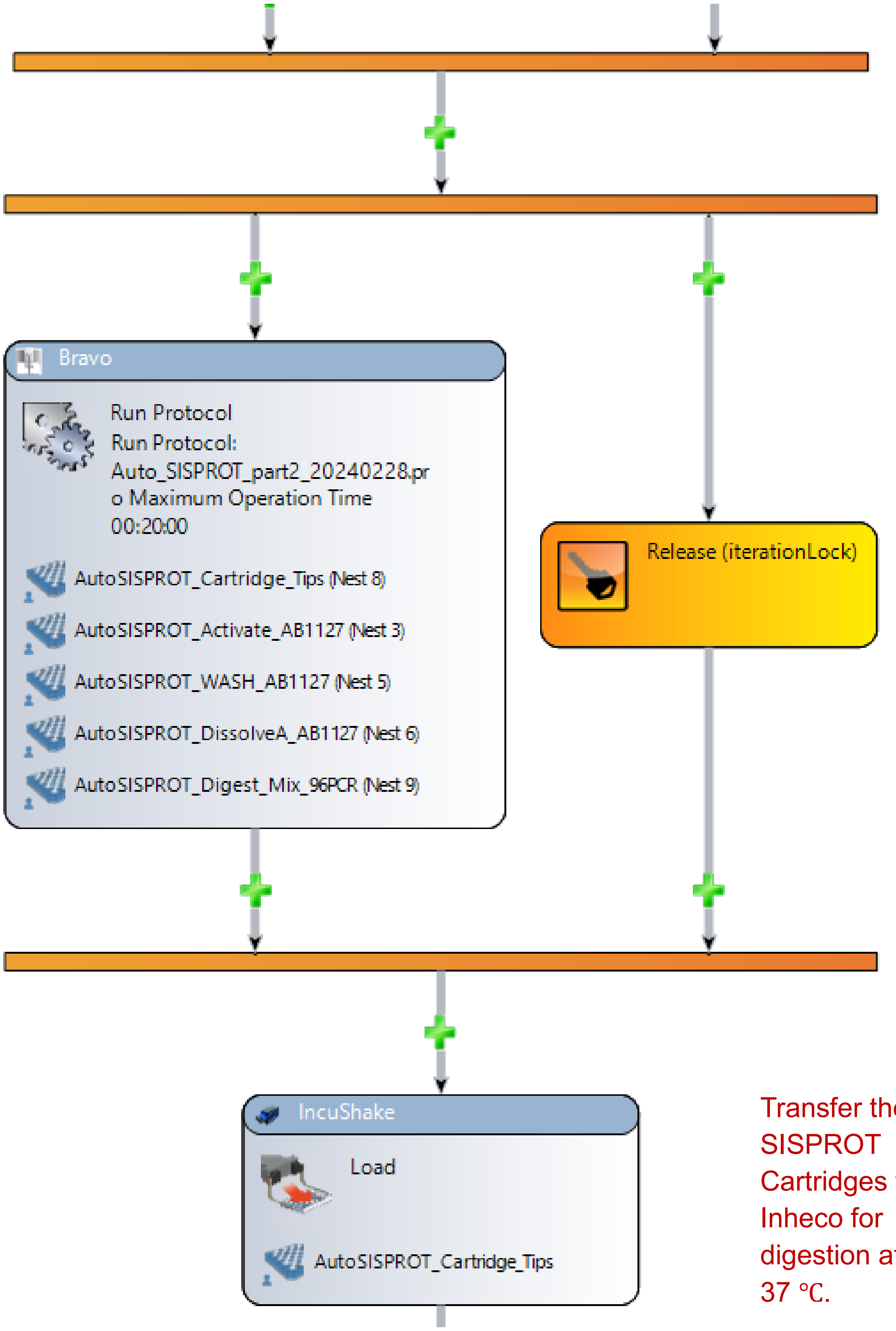

Run the Bravo protocol for pH transformation and adding the Digest Mix.

Transfer the SISPROT Cartridges to Inheco for digestion at 37 ℃.

Prepare Inheco to 37 ℃.

Incubate the SISPROT Cartridges in Inheco for digestion at 37 ℃ for 1h 20min.

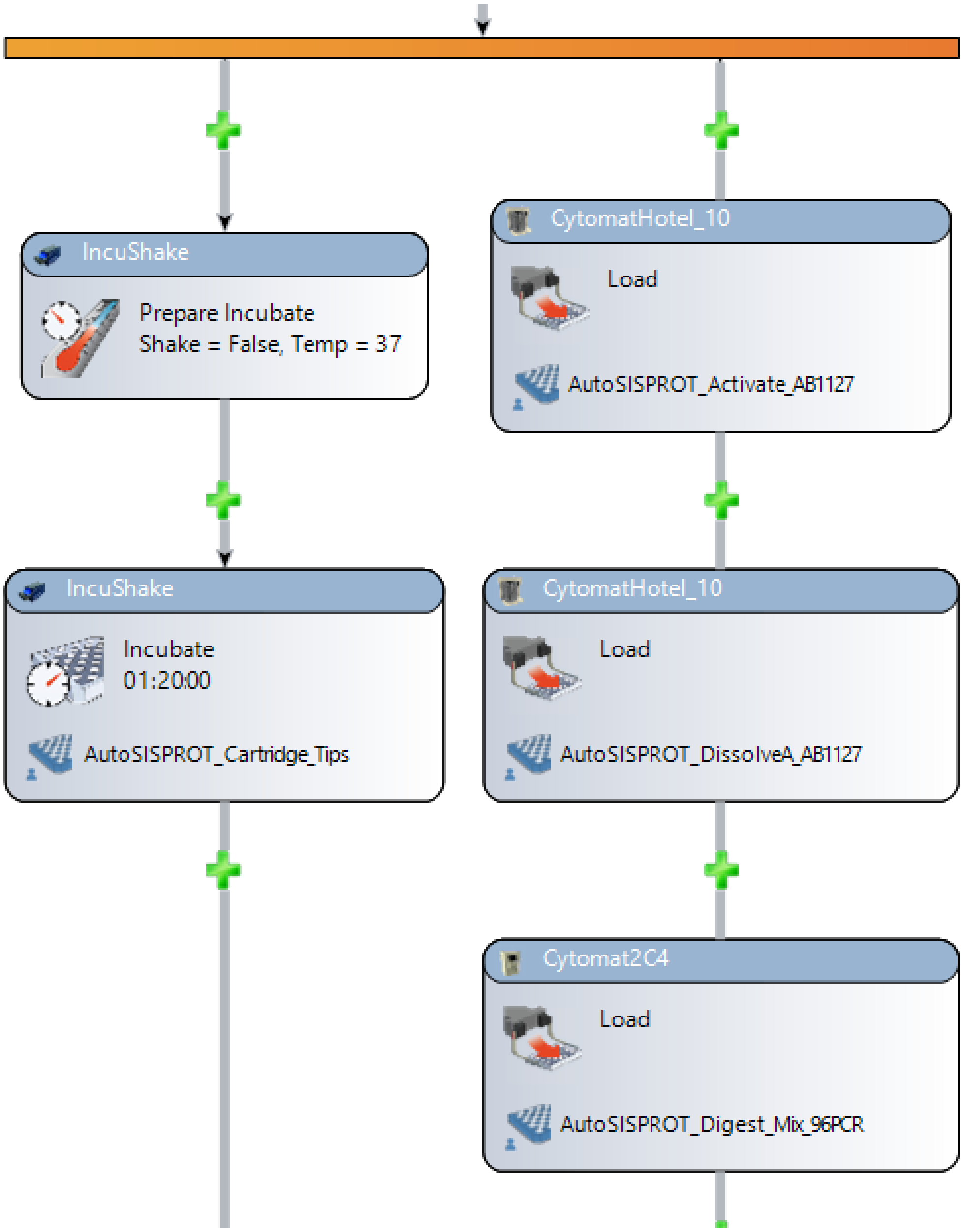

Move the Buffer Activate, Dissolve A and Digest Mix to Cytomat 10 hotel.

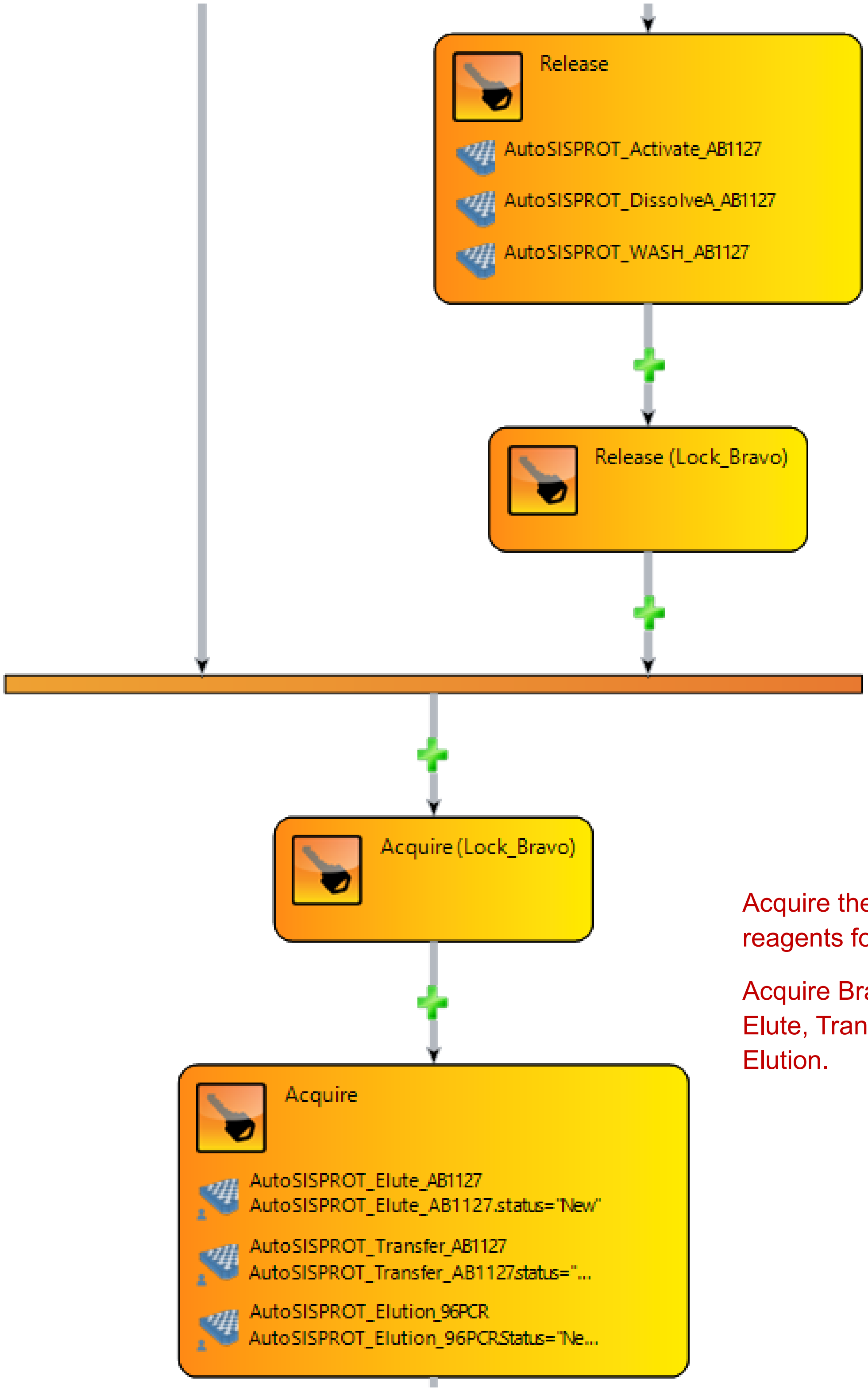

Release the Buffer Activate, Dissolve A, Wash and Bravo.

Acquire the instrument and reagents for the next step.

Acquire Bravo, Buffer Elute, Transfer, and Elution.

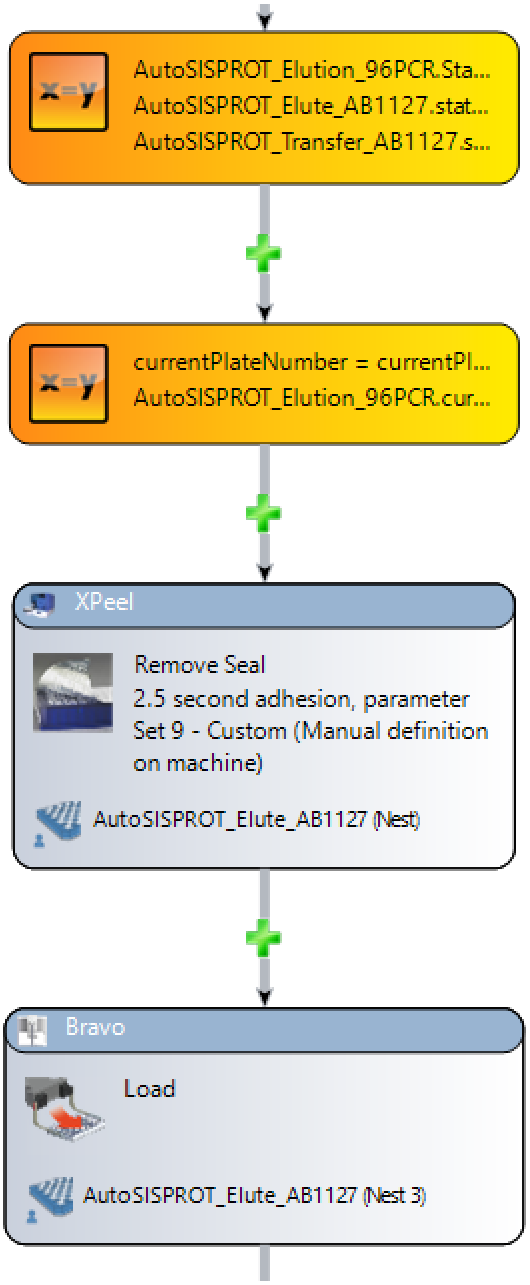

Identify the status of reagents and labware.

Peel off the film from the Buffer Elute container.

Load the Buffer Elute to Bravo.

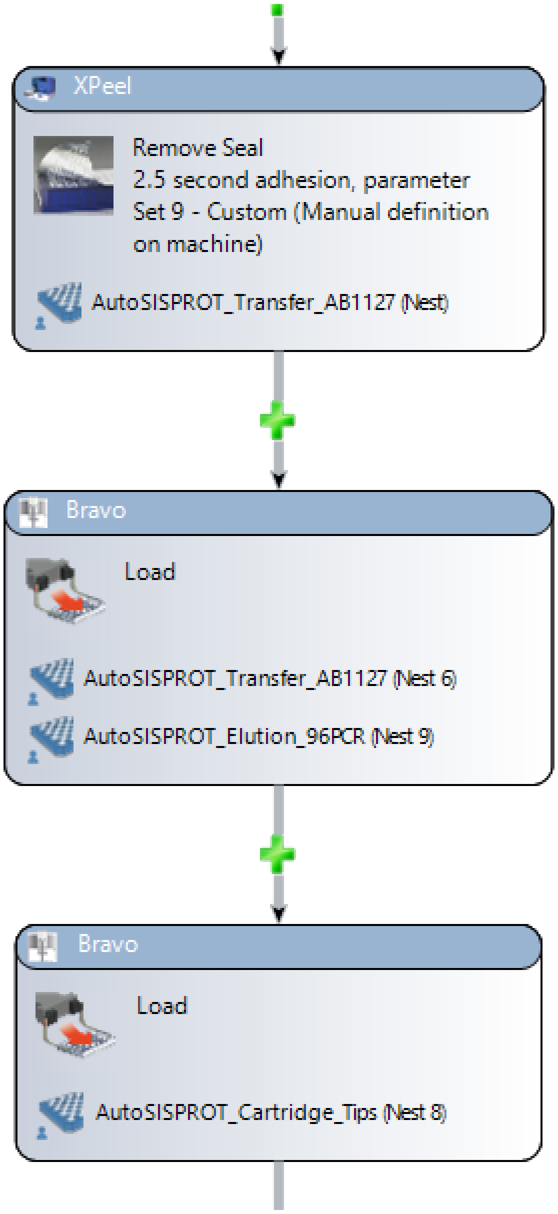

Peel off the film from the Buffer Transfer container.

Load the Buffer Transfer and the plate for collection elution to Bravo.

Transfer SISPROT Cartridges back to Bravo.

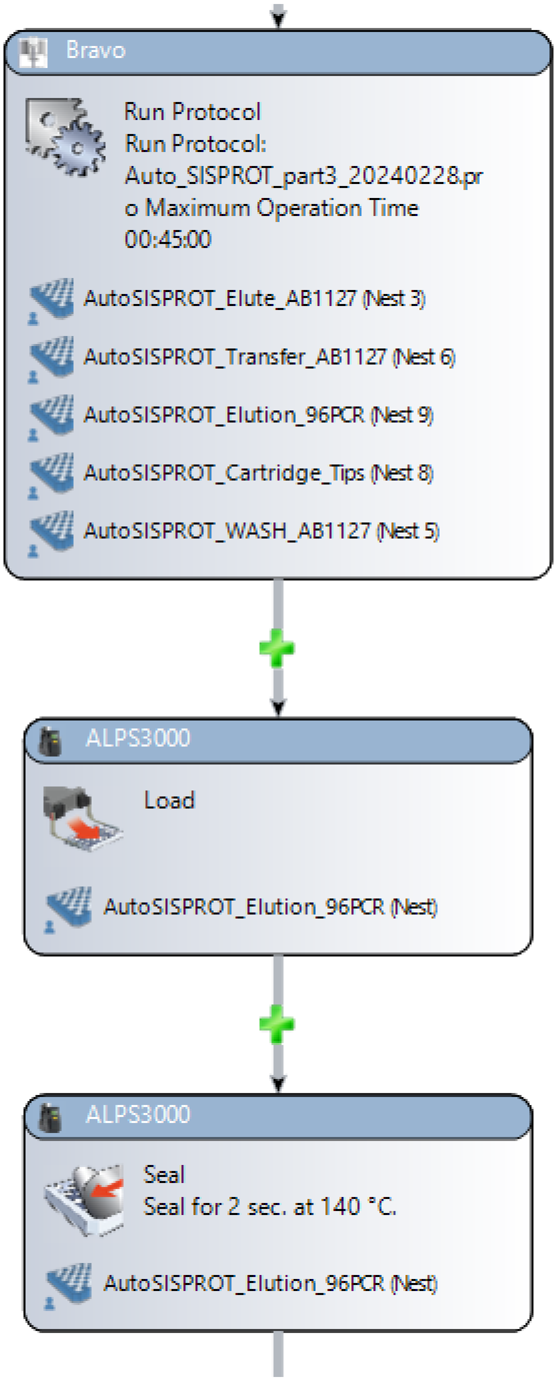

Run the Bravo protocol for desalting and eluting peptides.

Load the elution plate to ALPS300.

Seal the elution plate by ALPS300.

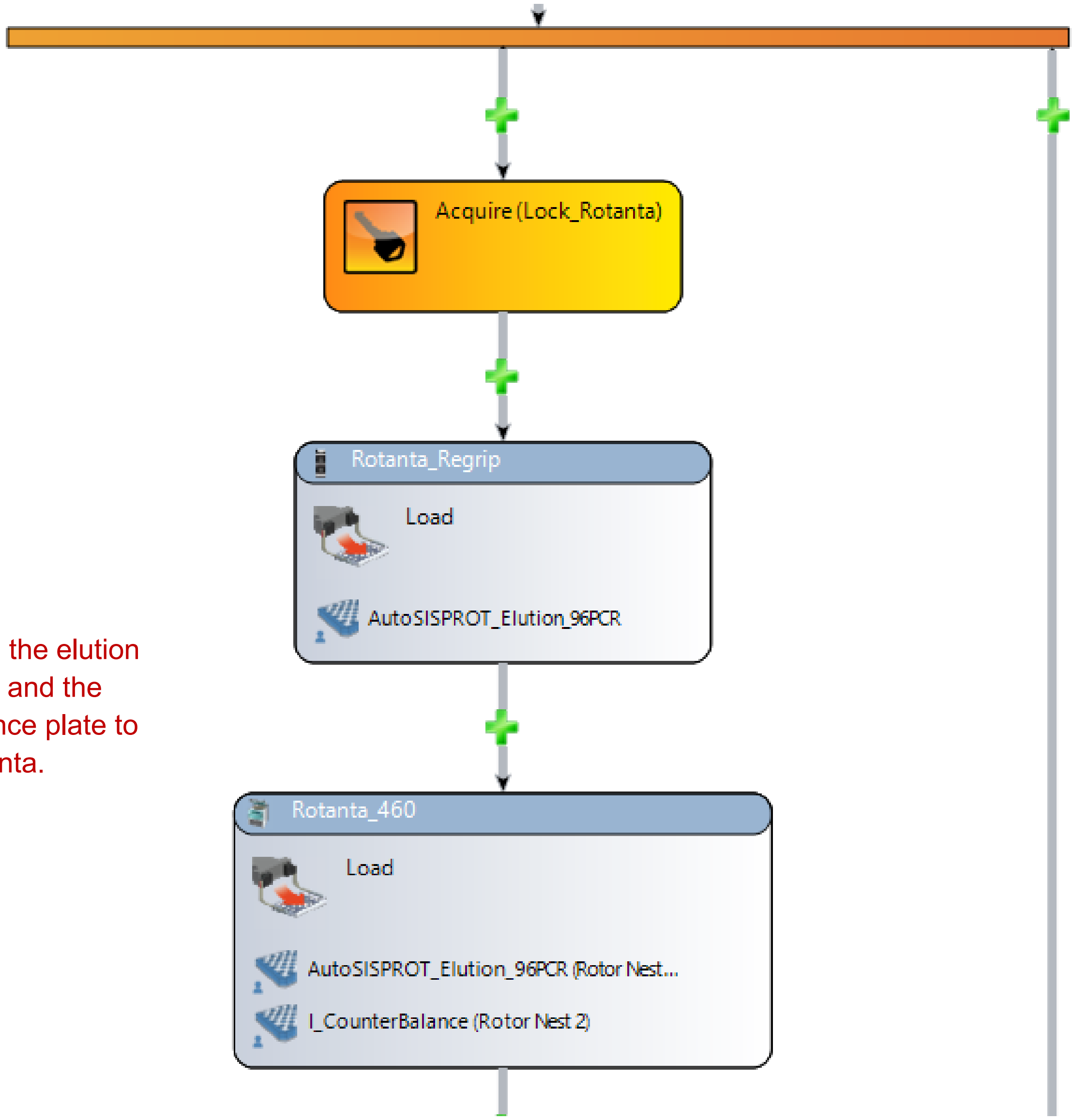

Load the elution plate and the balance plate to Rotanta.

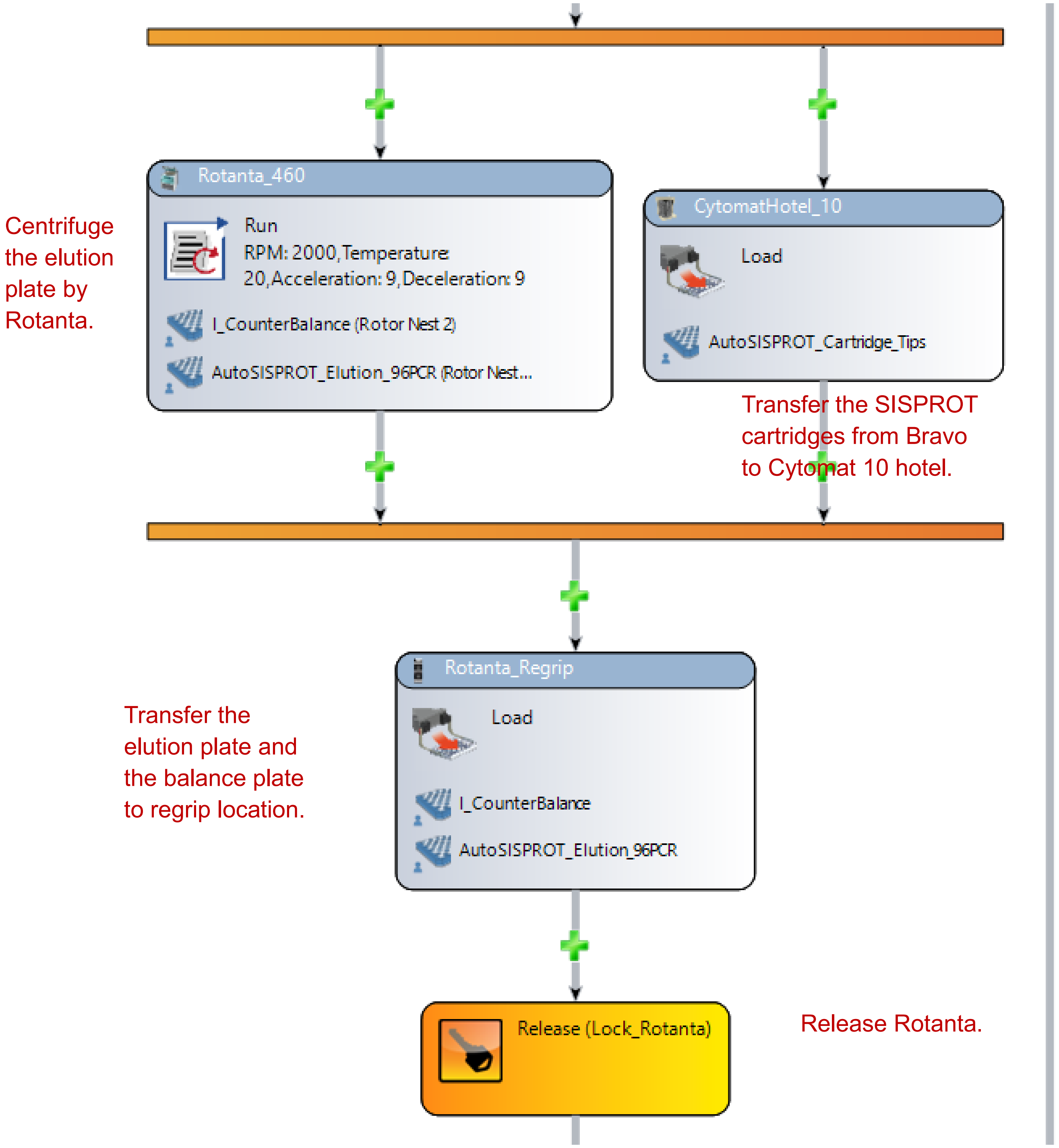
Centrifuge the elution plate by Rotanta.
Rotanta_460
Run
RPM: 2000, Temperature: 20, Acceleration: 9, Deceleration: 9
I_CounterBalance (Rotor Nest 2)
AutoSISPROT_Elution_96PCR (Rotor Nest...
CytomatHotel_10
Load
AutoSISPROT_Cartridge_Tips
Transfer the SISPROT cartridges from Bravo to Cytomat 10 hotel.
Transfer the elution plate and the balance plate to regrip location.
Rotanta_Regrip
Load
I_CounterBalance
AutoSISPROT_Elution_96PCR
Release (Lock_Rotanta)
Release Rotanta.

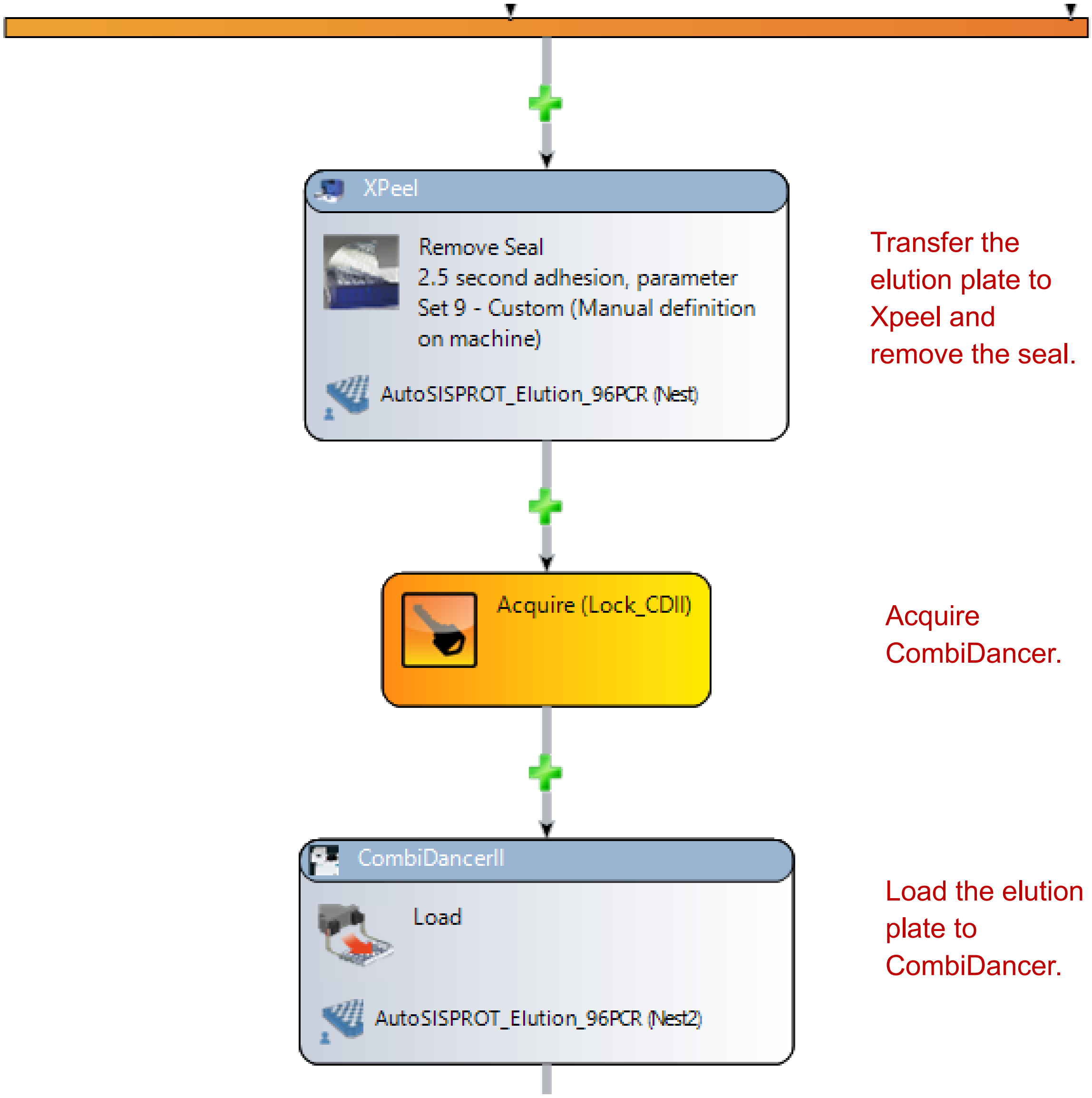

Transfer the elution plate to Xpeel and remove the seal.

Acquire CombiDancer.

Load the elution plate to CombiDancer.

Dry down eluted peptides by CombiDancer.

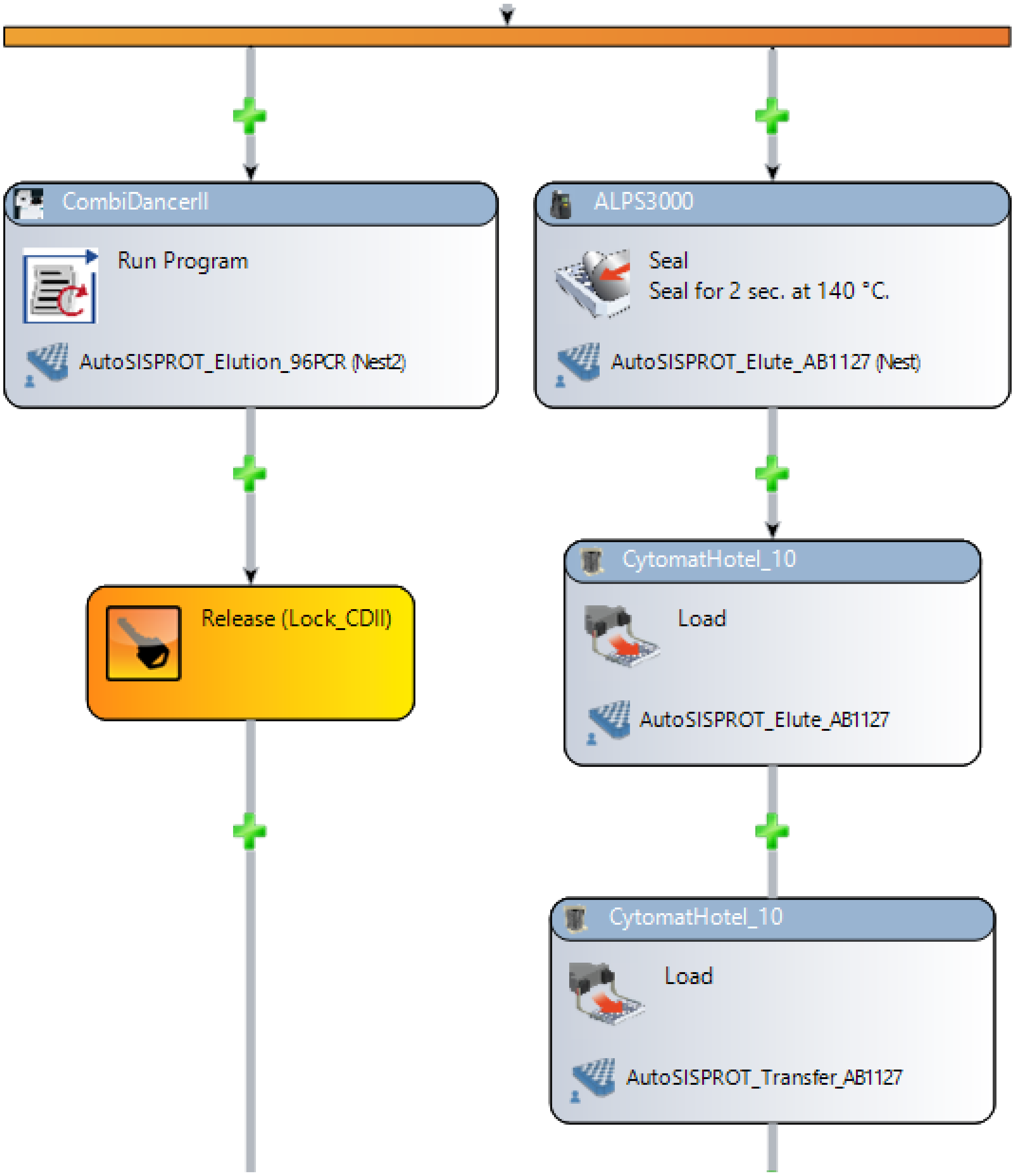

Seal the plate of Buffer Elute by ALPS3000.

Transfer the plate of Buffer Elute to Cytomat 10 hotel.

Transfer the plate of Buffer Transfer to Cytomat 10 hotel.

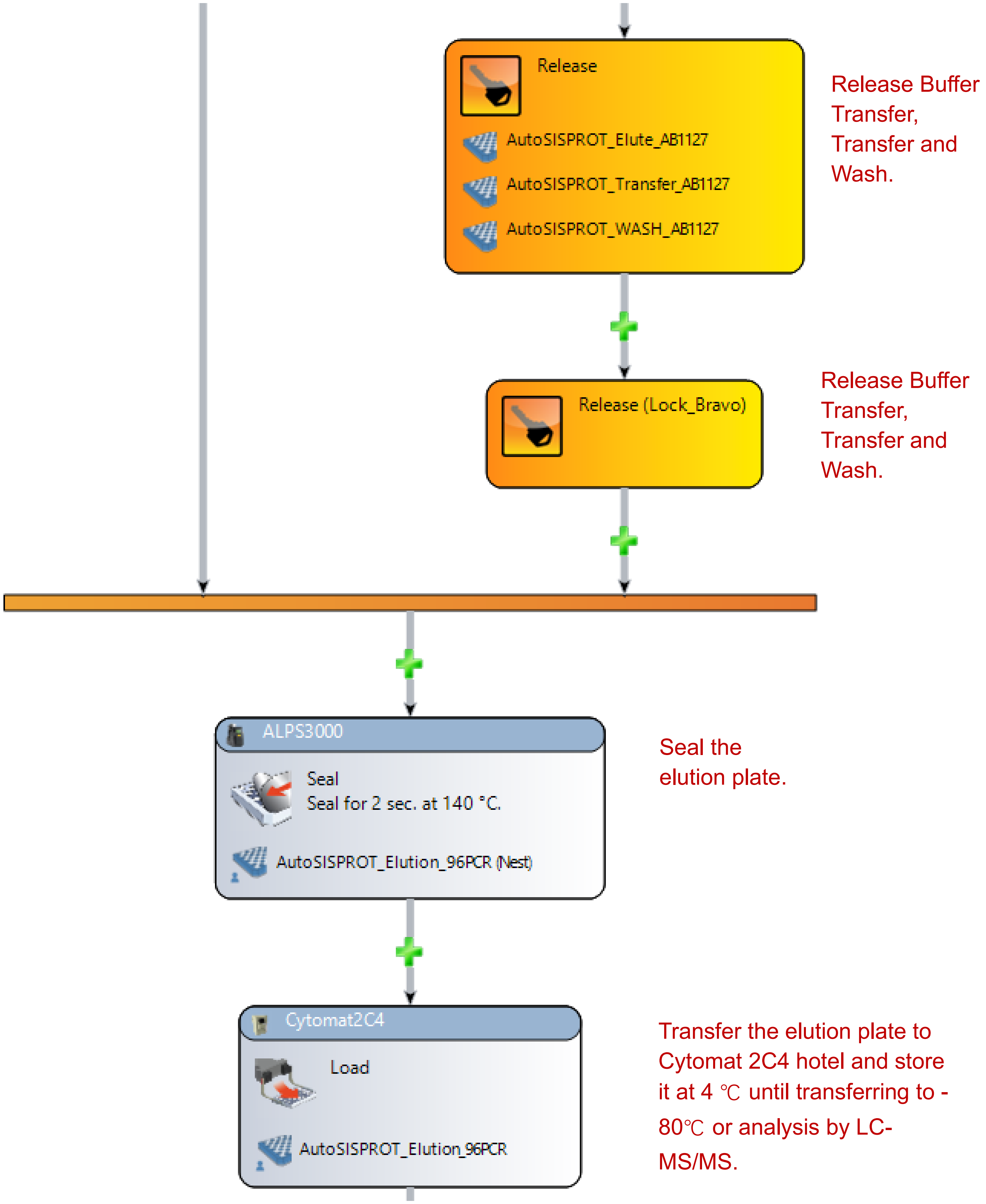
Release
AutoSISPROT_Elute_AB1127
AutoSISPROT_Transfer_AB1127
AutoSISPROT_WASH_AB1127
Release Buffer Transfer, Transfer and Wash.
Release (Lock_Bravo)
Release Buffer Transfer, Transfer and Wash.
ALPS3000
Seal
Seal for 2 sec. at 140 °C.
AutoSISPROT_Elution_96PCR (Nest)
Seal the elution plate.
Cytomat2C4
Load
AutoSISPROT_Elution_96PCR
Transfer the elution plate to Cytomat 2C4 hotel and store it at 4 ℃ until transferring to -80℃ or analysis by LC-MS/MS.

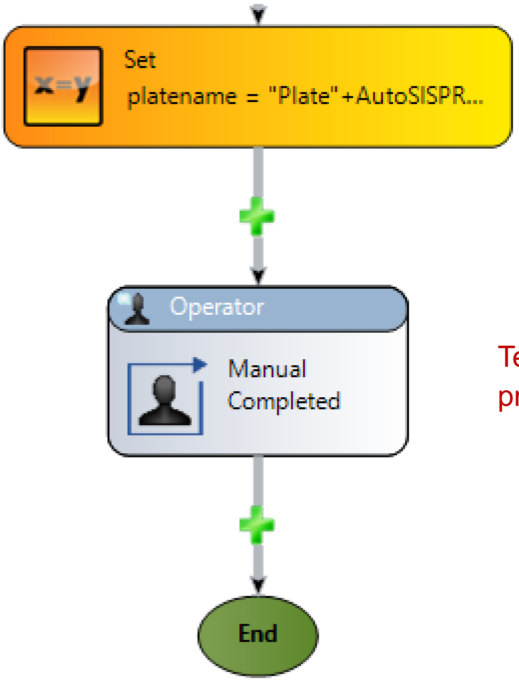

Set the plate name.

Tell the operator that the process is completed.

2. The process for generating cell line proteome data

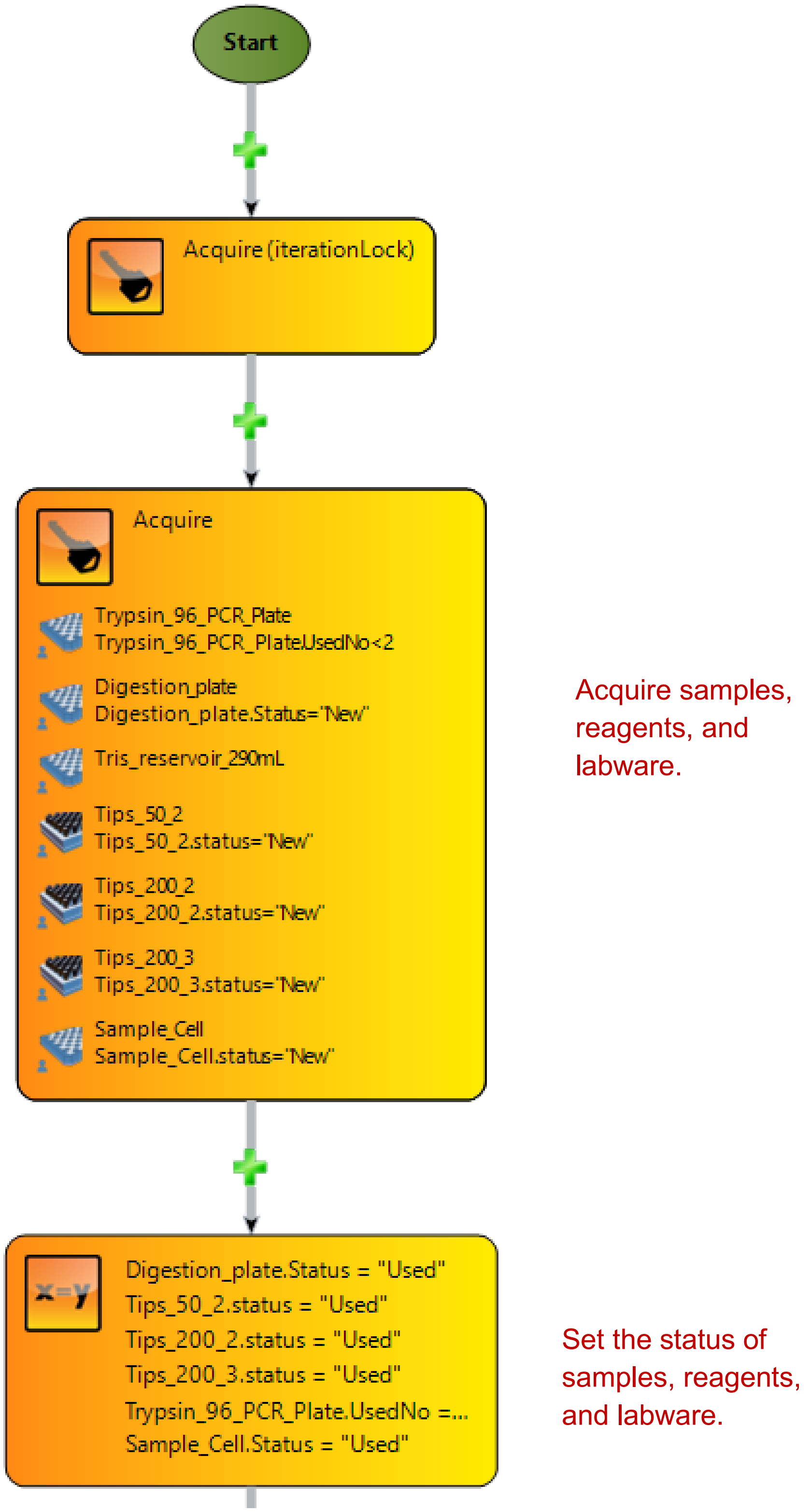

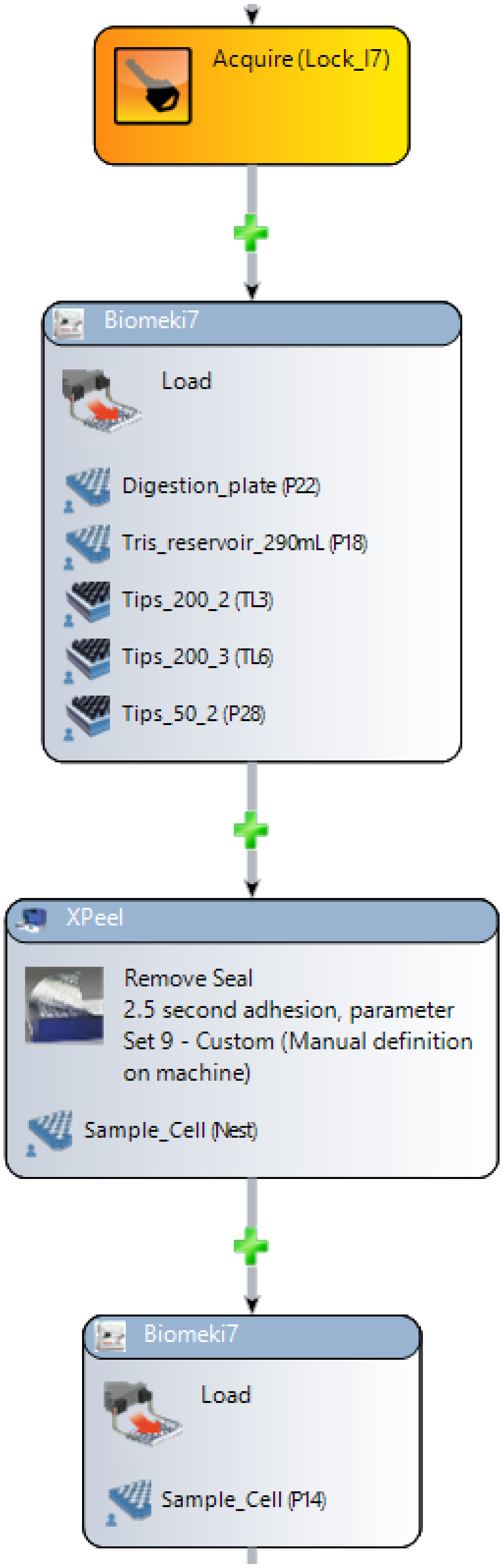

Acquire Biomek i7.

Load the sample plates and tips to Biomek i7.

Peel off the film of the sample plate.

Load the sample plate to Biomek i7.

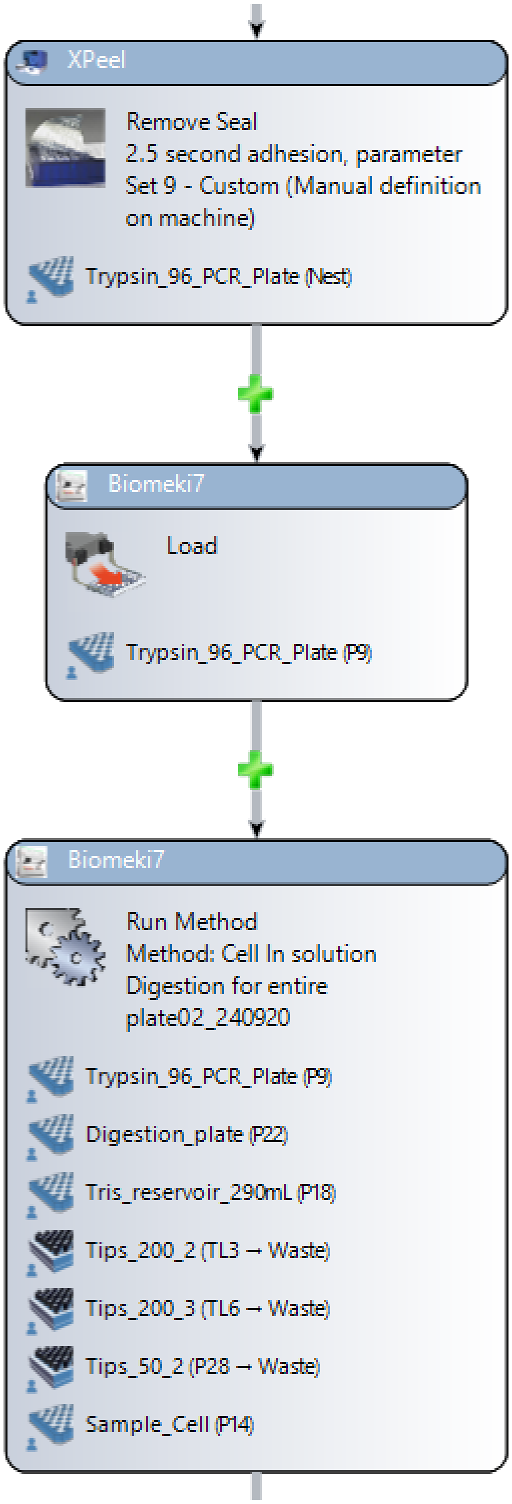

Peel off the film of the trypsin plate.

Load the trypsin plate to Biomek i7.

Run the method for adjusting the volume of samples and adding trypsin on Biomek i7.

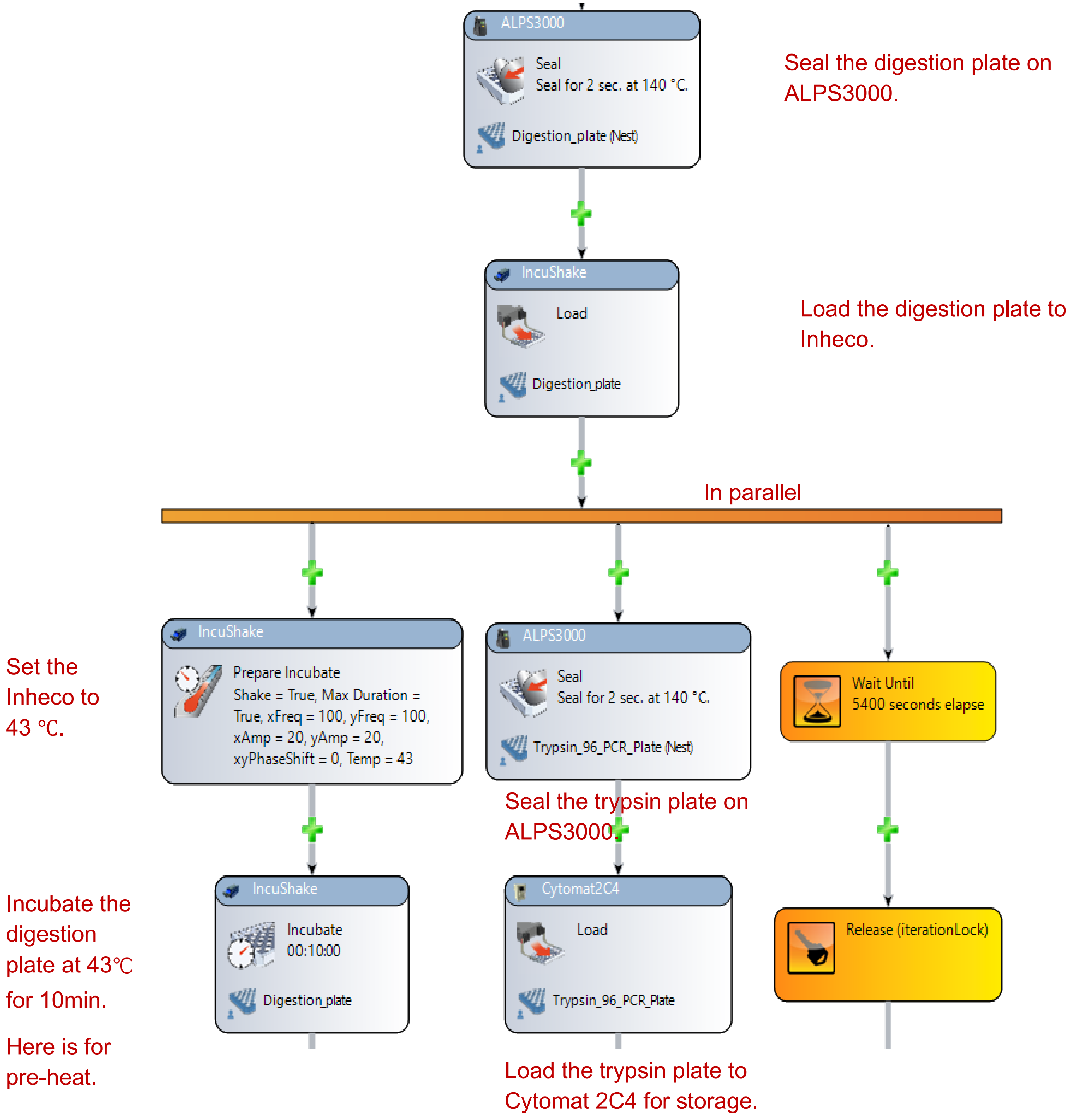
ALPS3000
Seal
Seal for 2 sec. at 140 °C.
Digestion_plate (Nest)
Seal the digestion plate on ALPS3000.
IncuShake
Load
Digestion_plate
Load the digestion plate to Inheco.
In parallel
IncuShake
Prepare Incubate
Shake = True, Max Duration = True, xFreq = 100, yFreq = 100, xAmp = 20, yAmp = 20, xyPhaseShift = 0, Temp = 43
Set the Inheco to 43 ℃.
IncuShake
Incubate
00:10:00
Digestion_plate
Incubate the digestion plate at 43℃ for 10min.
Here is for pre-heat.
ALPS3000
Seal
Seal for 2 sec. at 140 °C.
Trypsin_96_PCR_Plate (Nest)
Seal the trypsin plate on ALPS3000.
Cytomat2C4
Load
Trypsin_96_PCR_Plate
Load the trypsin plate to Cytomat 2C4 for storage.
Wait Until
5400 seconds elapse
Release (iterationLock)

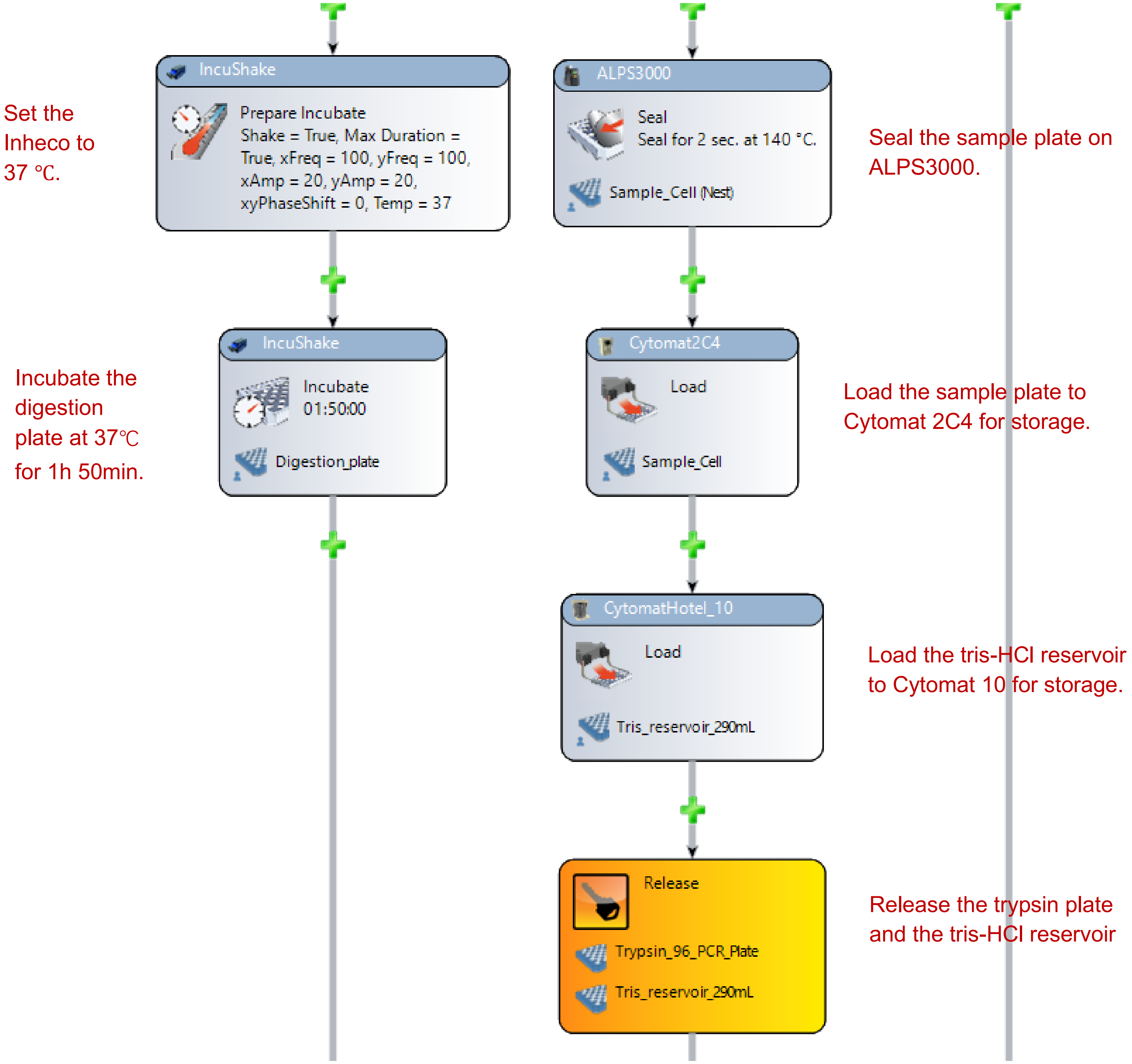
Set the Inheco to 37 ℃.
IncuShake
Prepare Incubate
Shake = True, Max Duration = True, xFreq = 100, yFreq = 100, xAmp = 20, yAmp = 20, xyPhaseShift = 0, Temp = 37
ALPS3000
Seal
Seal for 2 sec. at 140 °C.
Sample_Cell (Nest)
Seal the sample plate on ALPS3000.
Incubate the digestion plate at 37℃ for 1h 50min.
IncuShake
Incubate
01:50:00
Digestion_plate
Cytomat2C4
Load
Sample_Cell
Load the sample plate to Cytomat 2C4 for storage.
CytomatHotel_10
Load
Tris_reservoir_290mL
Load the tris-HCl reservoir to Cytomat 10 for storage.
Release
Trypsin_96_PCR_Plate
Tris_reservoir_290mL
Release the trypsin plate and the tris-HCl reservoir

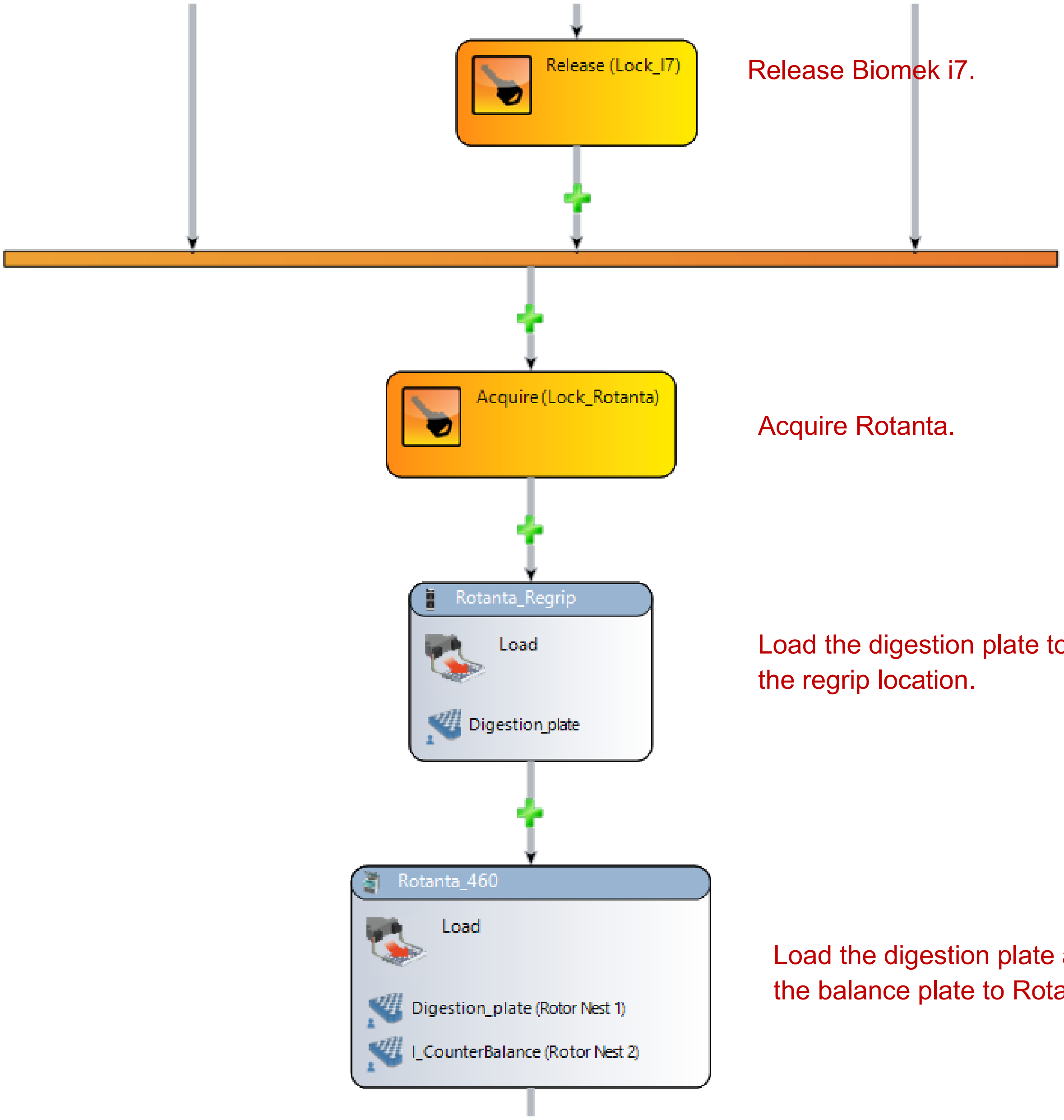

Release Biomek i7.

Acquire Rotanta.

Load the digestion plate to the regrip location.

Load the digestion plate and the balance plate to Rotanta.

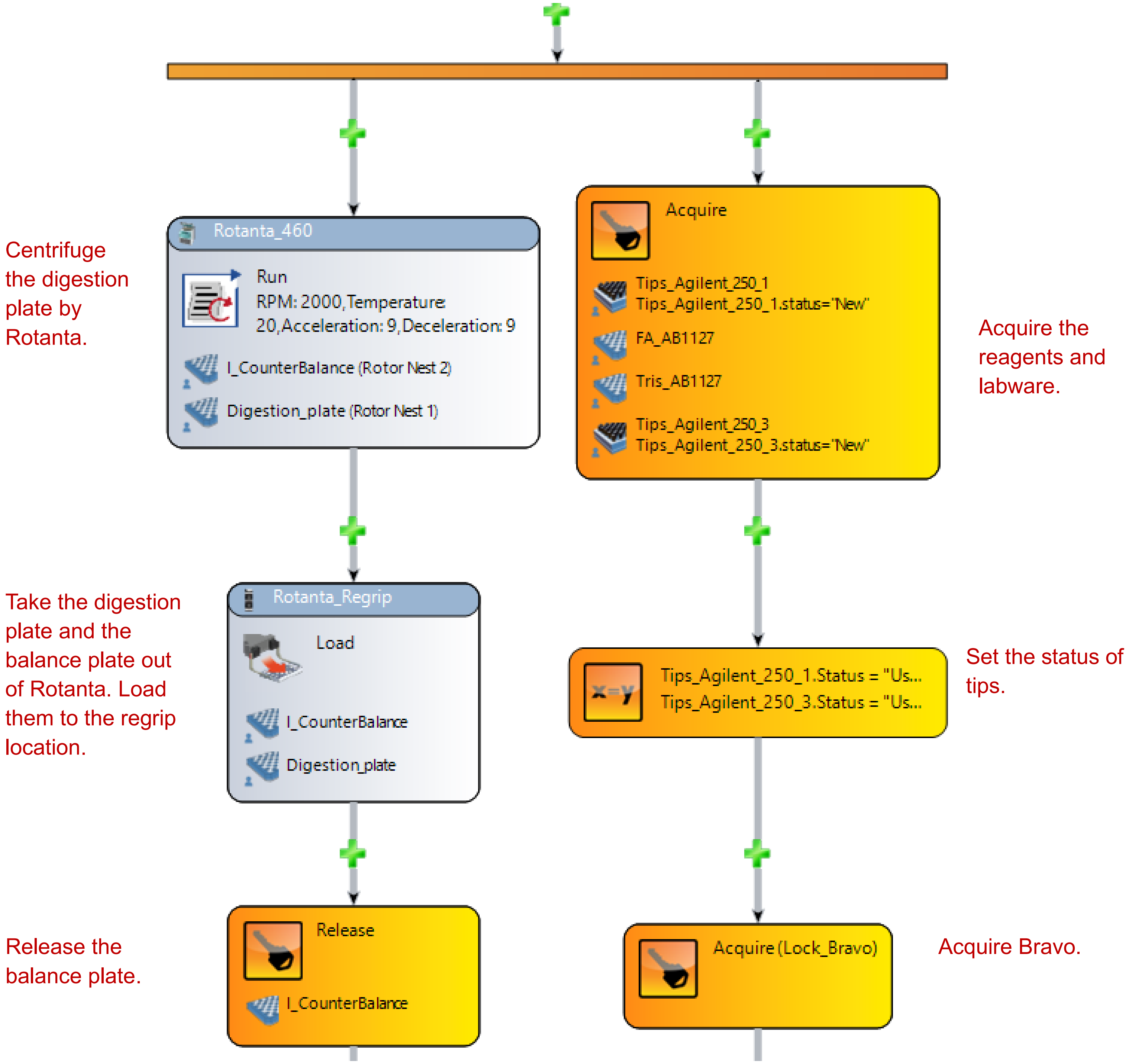
Centrifuge the digestion plate by Rotanta.
Rotanta_460
Run
RPM: 2000,Temperature: 20,Acceleration: 9,Deceleration: 9
l_CounterBalance (Rotor Nest 2)
Digestion_plate (Rotor Nest 1)
Acquire
Tips_Agilent_250_1
Tips_Agilent_250_1.status='New'
FA_AB1127
Tris_AB1127
Tips_Agilent_250_3
Tips_Agilent_250_3.status='New'
Acquire the reagents and labware.
Take the digestion plate and the balance plate out of Rotanta. Load them to the regrip location.
Rotanta_Regrip
Load
l_CounterBalance
Digestion_plate
Tips_Agilent_250_1.Status = "Us...
Tips_Agilent_250_3.Status = "Us...
Set the status of tips.
Release the balance plate.
Release
l_CounterBalance
Acquire (Lock_Bravo)
Acquire Bravo.

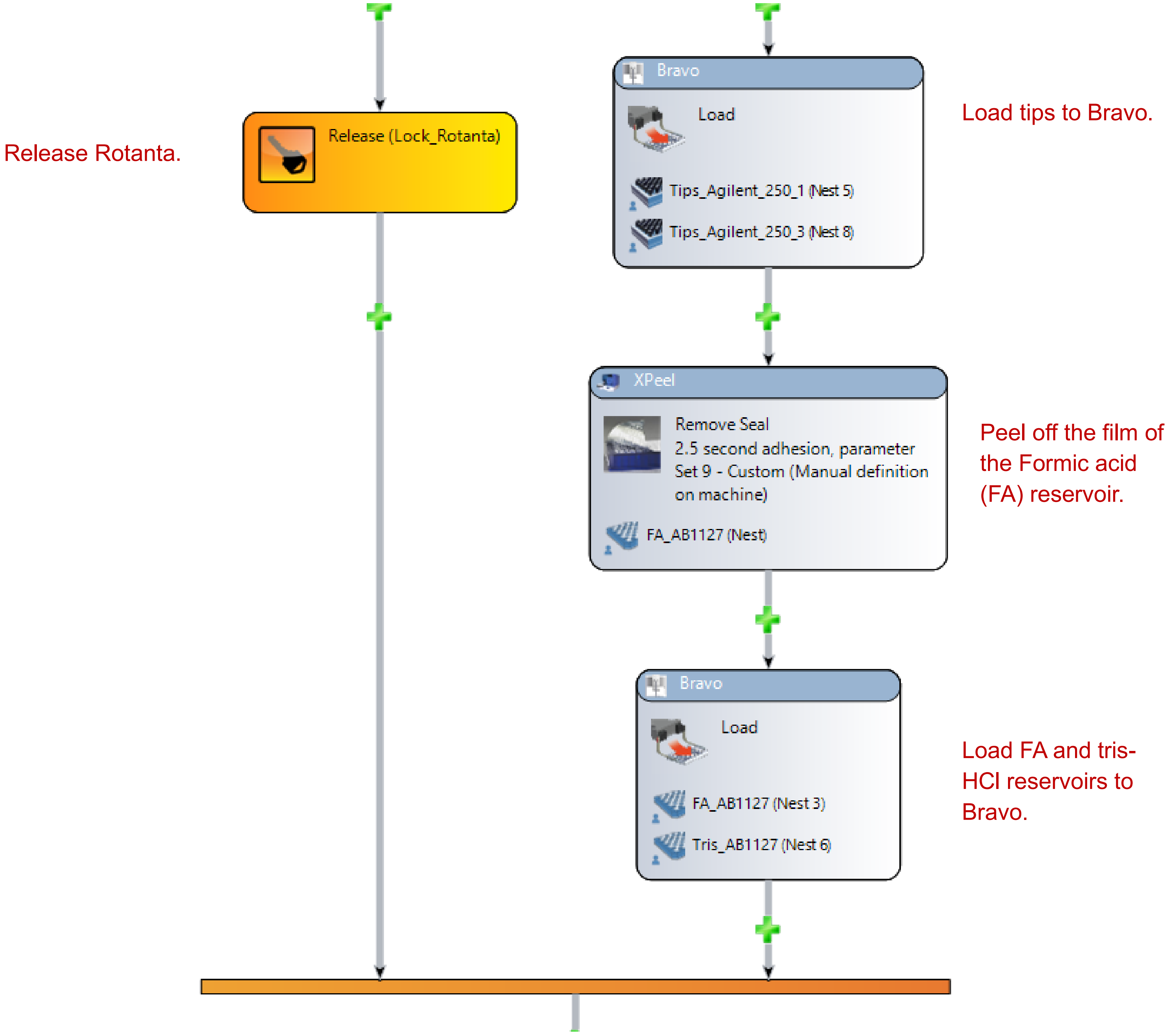
Release Rotanta.
Release (Lock_Rotanta)
Bravo
Load
Tips_Agilent_250_1 (Nest 5)
Tips_Agilent_250_3 (Nest 8)
Load tips to Bravo.
XPeel
Remove Seal
2.5 second adhesion, parameter Set 9 - Custom (Manual definition on machine)
FA_AB1127 (Nest)
Peel off the film of the Formic acid (FA) reservoir.
Bravo
Load
FA_AB1127 (Nest 3)
Tris_AB1127 (Nest 6)
Load FA and tris-HCl reservoirs to Bravo.

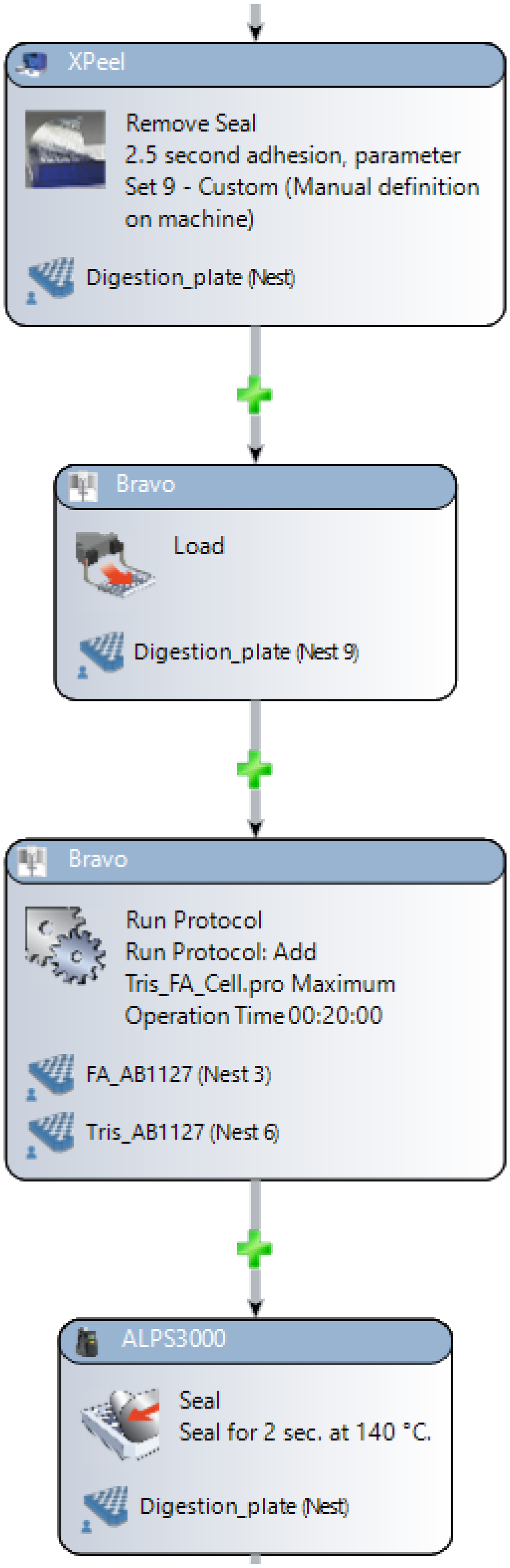

Peel off the film of the digestion plate.

Load the digestion plate to Bravo.

Run the Bravo protocol for terminating digestion.

Seal the digestion plate by ALPS3000.

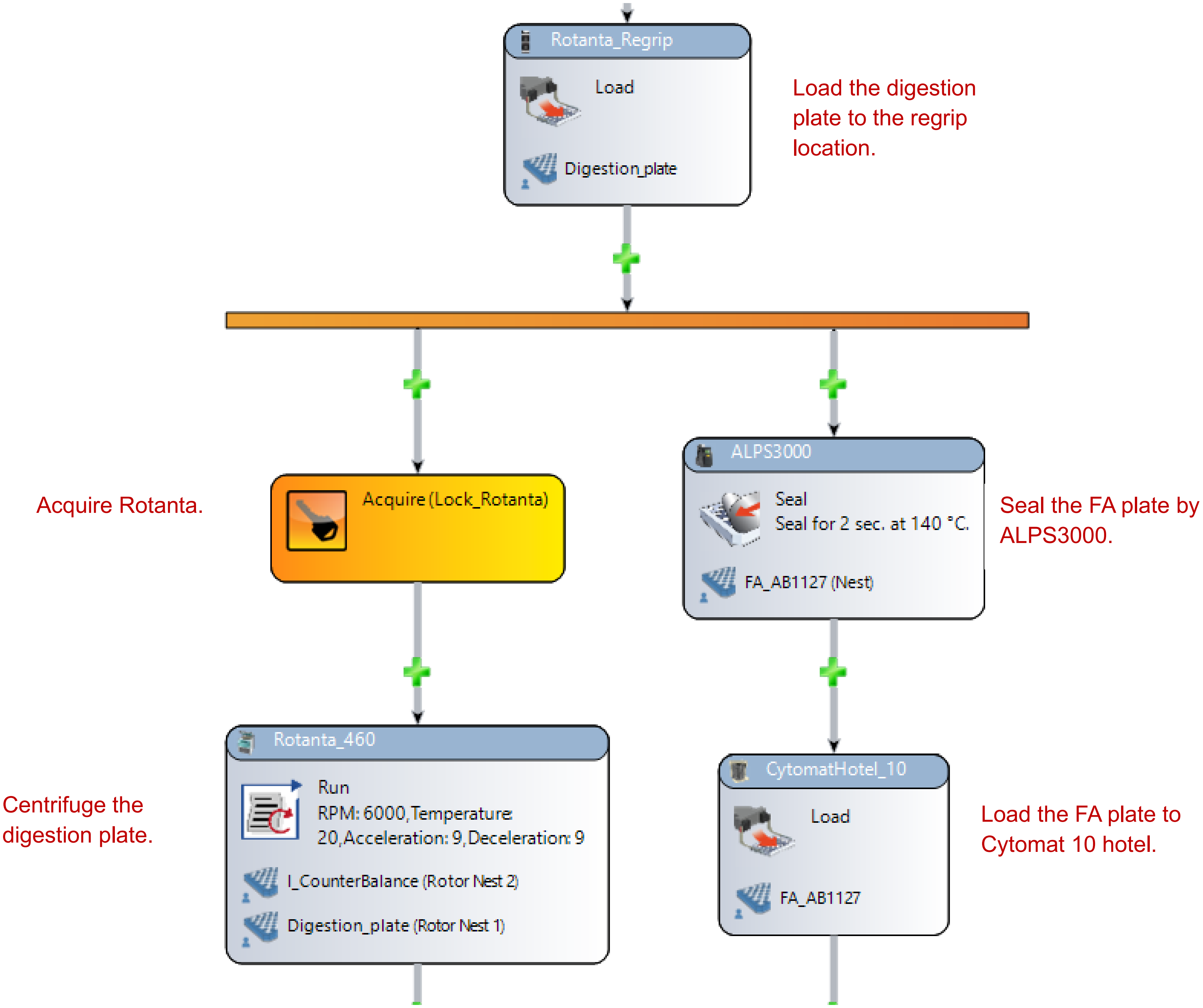

Rotanta_Regrip
Load
Digestion_plate
Load the digestion plate to the regrip location.
Acquire Rotanta.
Acquire (Lock_Rotanta)
ALPS3000
Seal
Seal for 2 sec. at 140 °C.
FA_AB1127 (Nest)
Seal the FA plate by ALPS3000.
Centrifuge the digestion plate.
Rotanta_460
Run
RPM: 6000, Temperature: 20, Acceleration: 9, Deceleration: 9
I_CounterBalance (Rotor Nest 2)
Digestion_plate (Rotor Nest 1)
CytomatHotel_10
Load
FA_AB1127
Load the FA plate to Cytomat 10 hotel.

Release Rotanta.

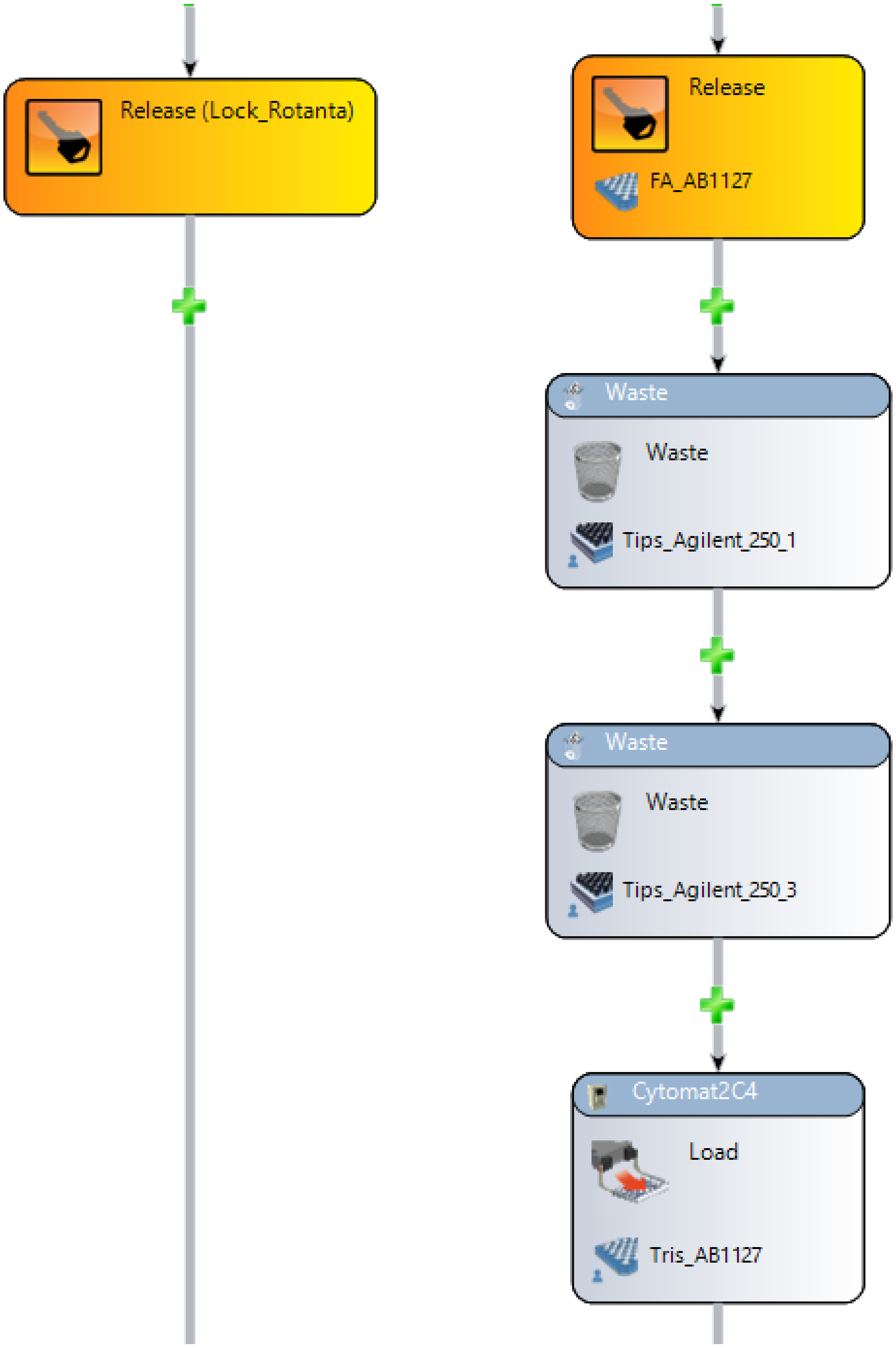

Release the FA plate.

Dispose of tips in the trash can.

Dispose of tips in the trash can.

Load tris-HCl reservoir to Cytomat 2C4.

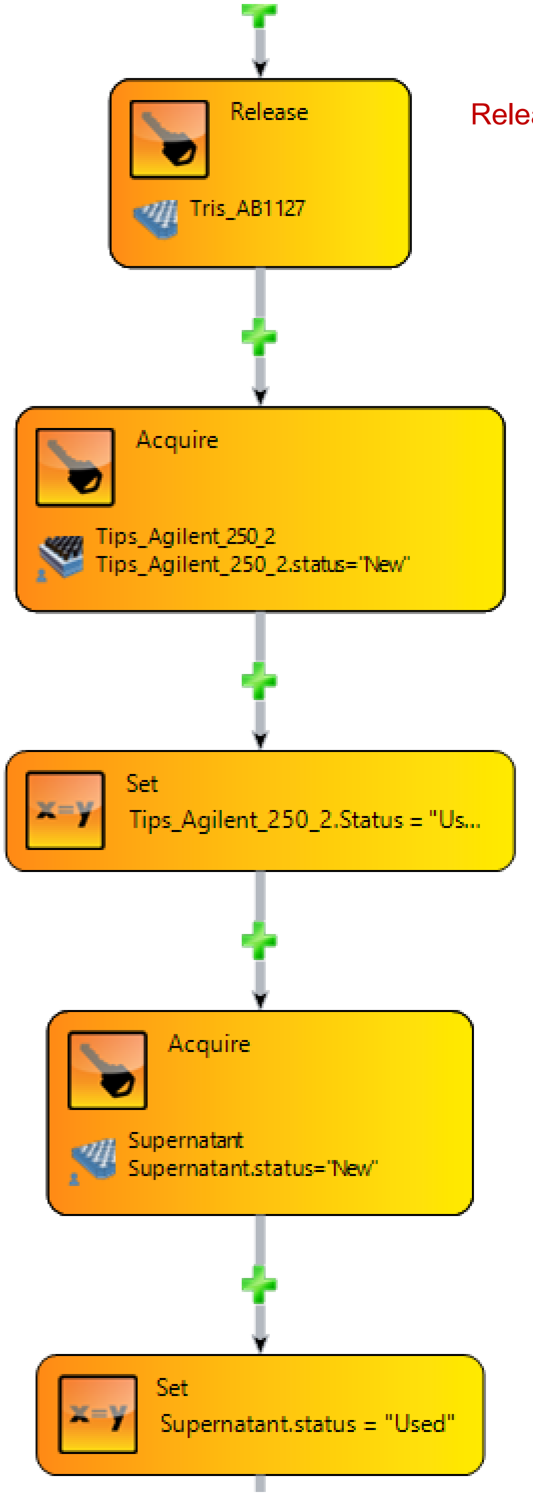

Release tris-HCl.

Acquire tips.

Set the status of tips.

Acquire a plate for supernatant.

Set the status of the supernatant plate.

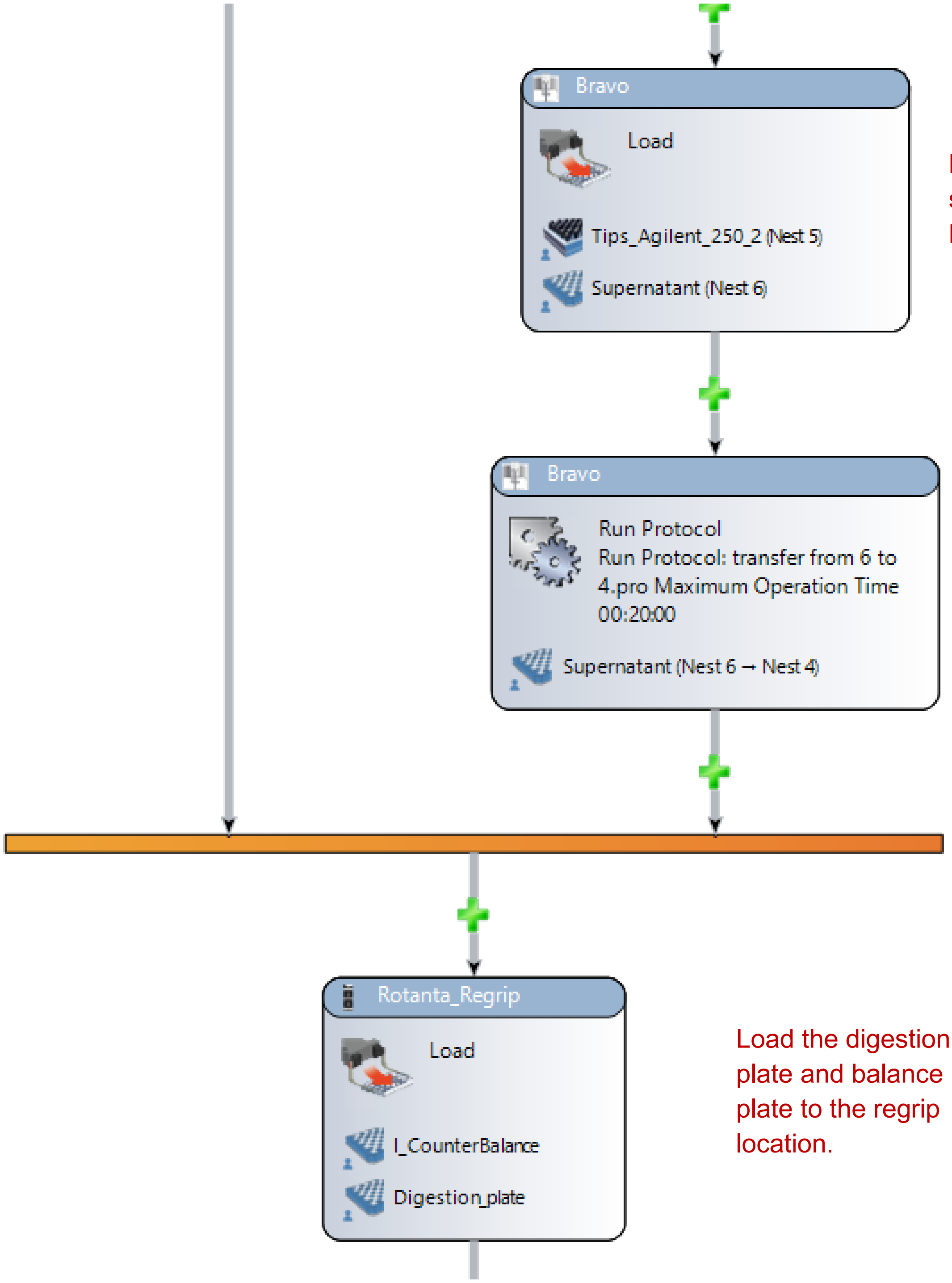

Load tips and the supernatant plate to Bravo.

Run the Bravo protocol to transfer the supernatant plate from 6 to 4.

Load the digestion plate and balance plate to the regrip location.

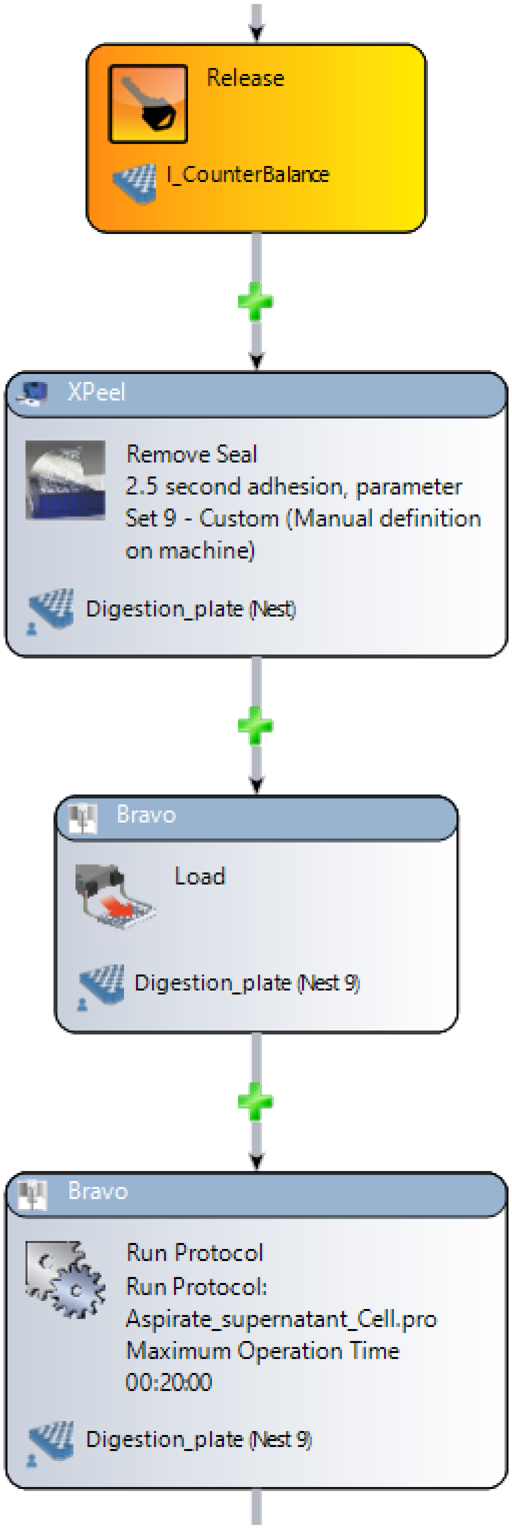

Release the balance plate.

Peel off the film of the digestion plate.

Load the digestion plate to Bravo.

Run the Bravo protocol to transfer the supernatant from the digestion plate to the supernatant plate.

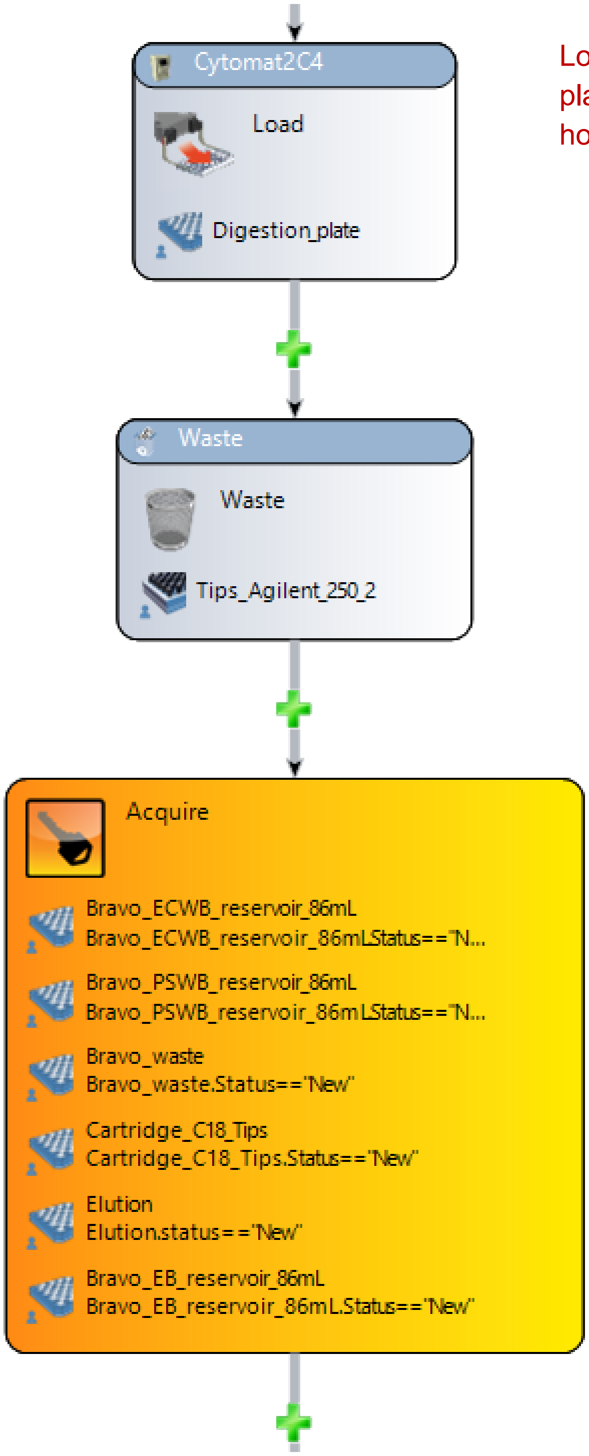

Load the digestion plate to Cytomat 2C4 hotel.

Dispose of tips in the trash can.

Acquire reagents and labware (e.g., tips and cartridges) for desalting.

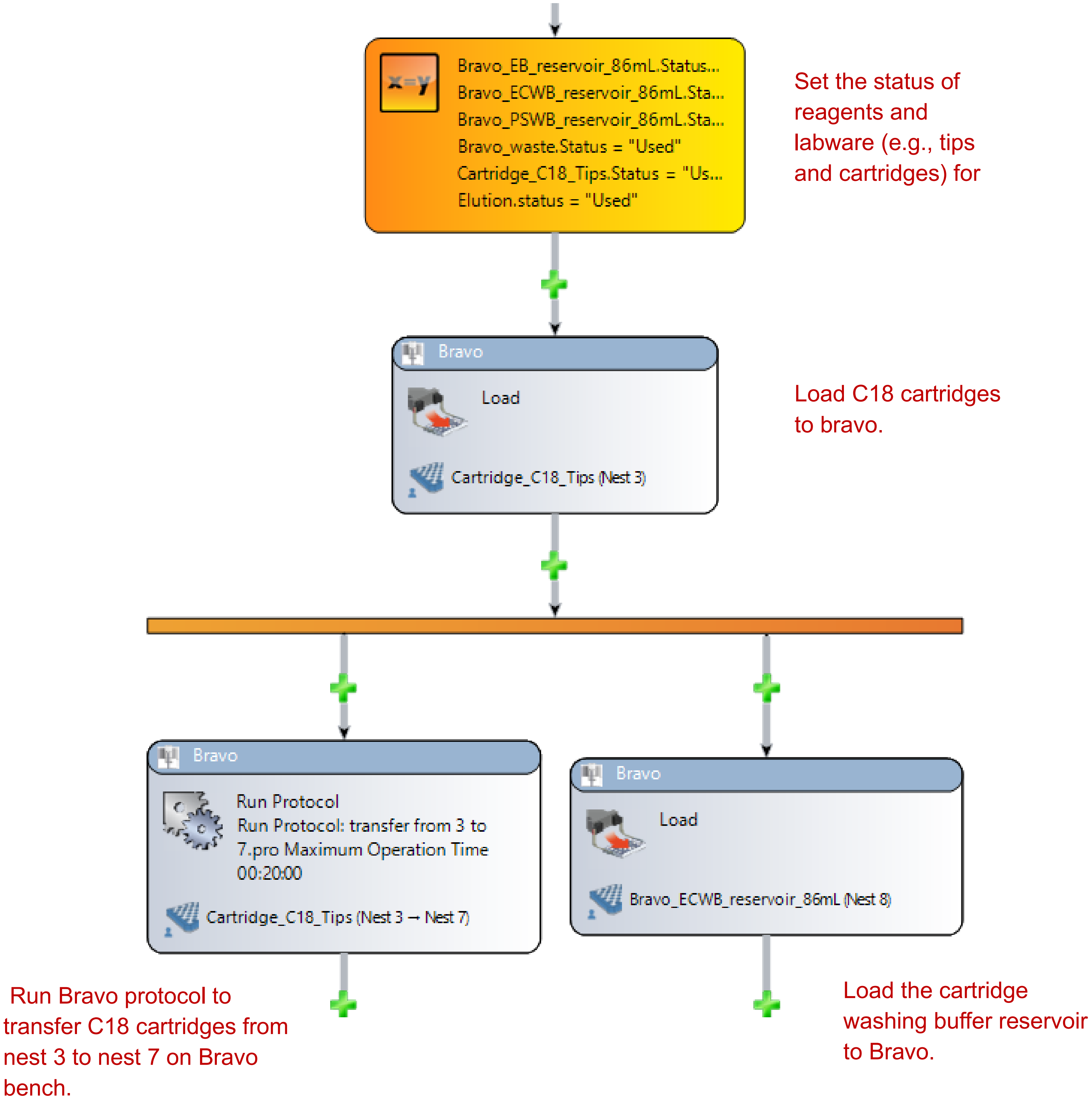
Bravo_EB_reservoir_86mL.Status...
Bravo_ECWB_reservoir_86mL.Sta...
Bravo_PSWB_reservoir_86mL.Sta...
Bravo_waste.Status = "Used"
Cartridge_C18_Tips.Status = "Us...
Elution.status = "Used"
Set the status of reagents and labware (e.g., tips and cartridges) for
Bravo
Load
Cartridge_C18_Tips (Nest 3)
Load C18 cartridges to bravo.
Bravo
Run Protocol
Run Protocol: transfer from 3 to 7.pro Maximum Operation Time 00:20:00
Cartridge_C18_Tips (Nest 3 → Nest 7)
Bravo
Load
Bravo_ECWB_reservoir_86mL (Nest 8)
Run Bravo protocol to transfer C18 cartridges from nest 3 to nest 7 on Bravo bench.
Load the cartridge washing buffer reservoir to Bravo.

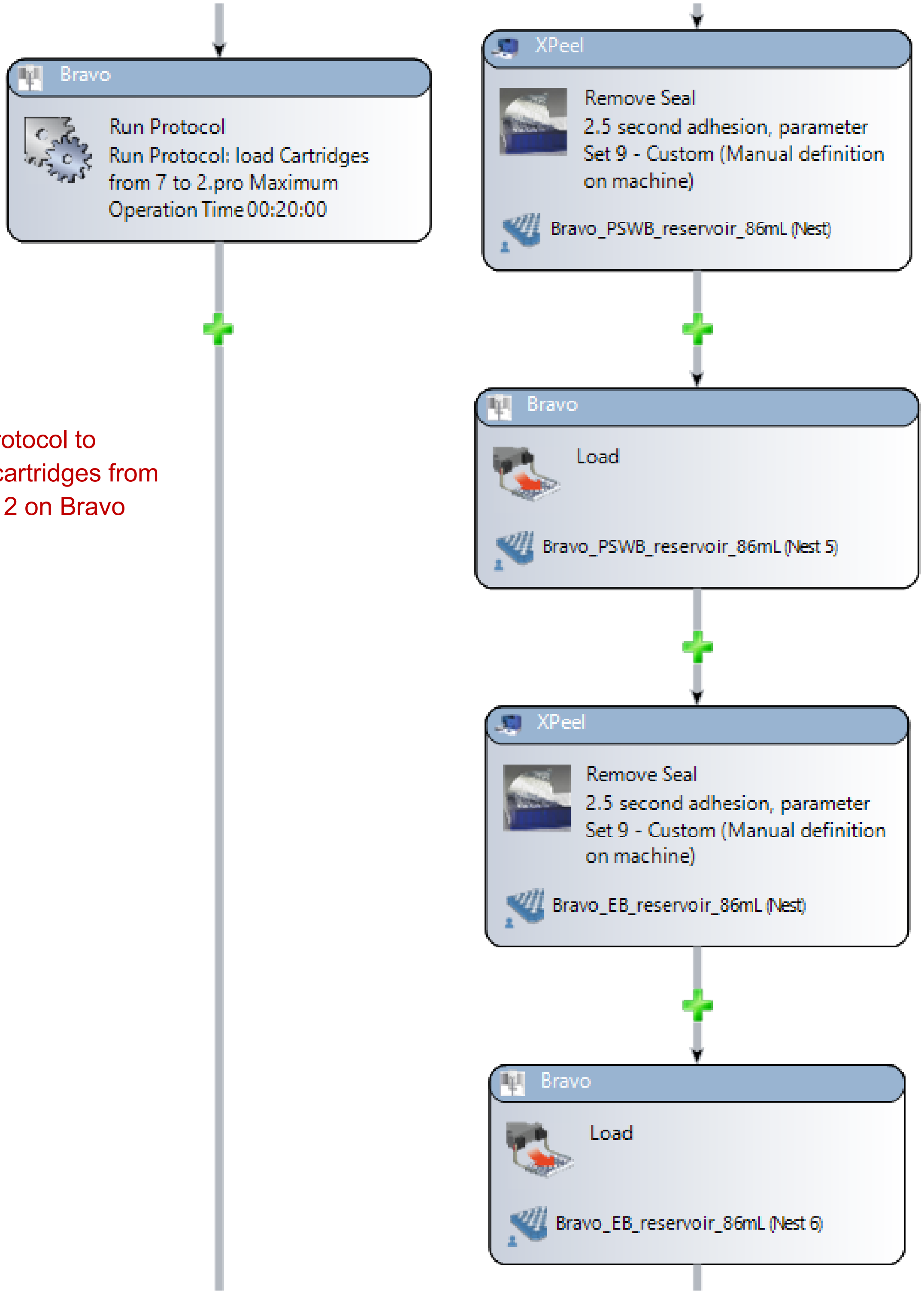

Run Bravo protocol to transfer C18 cartridges from nest 7 to nest 2 on Bravo bench.

Peel off the film of the washing buffer reservoir.

Load the washing buffer reservoir to Bravo.

Peel off the film of the eluting buffer reservoir.

Load the eluting buffer reservoir to Bravo.

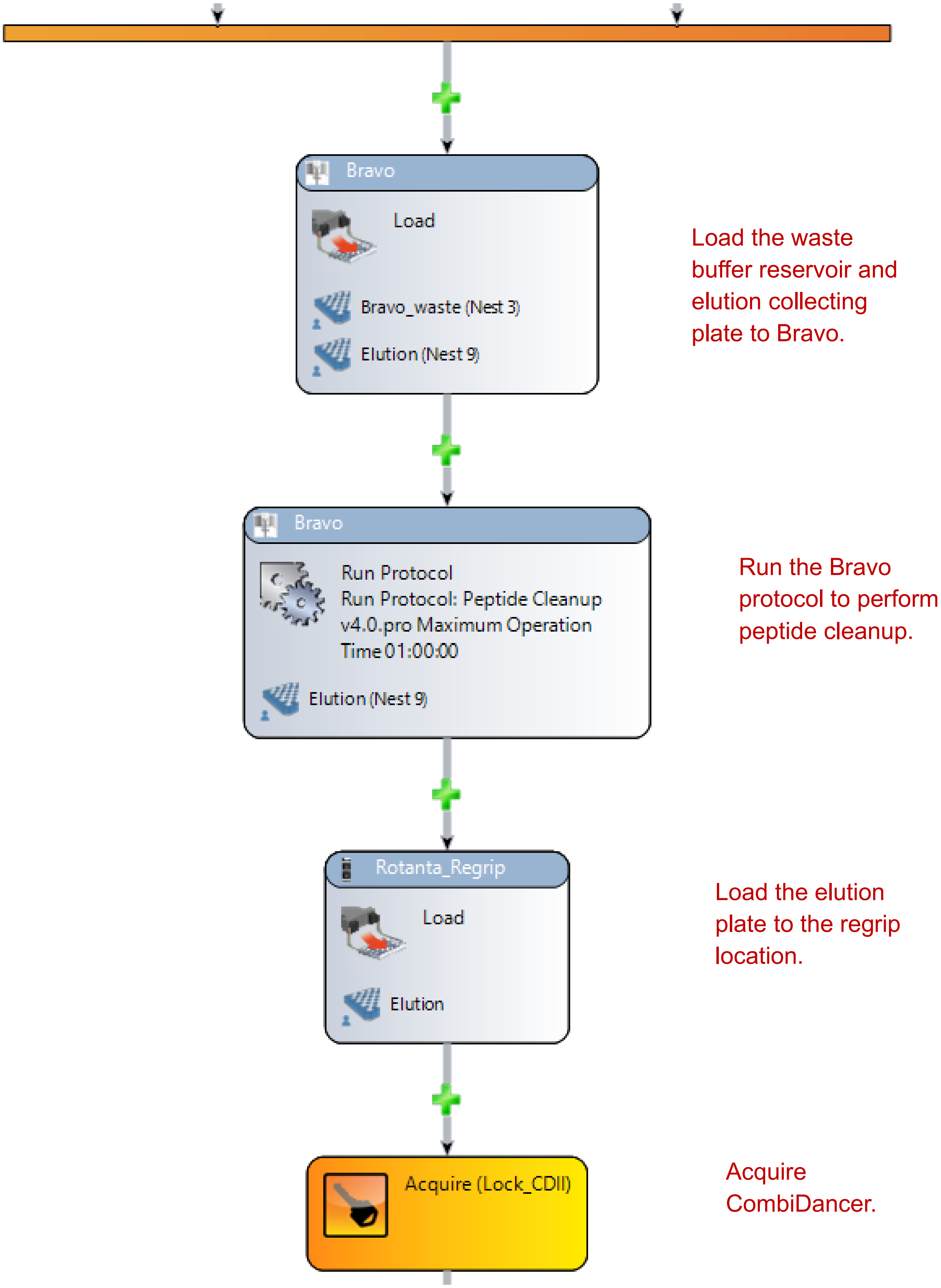
Bravo
Load
Bravo_waste (Nest 3)
Elution (Nest 9)
Bravo
Run Protocol
Run Protocol: Peptide Cleanup v4.0.pro Maximum Operation Time 01:00:00
Elution (Nest 9)
Rotanta_Regrip
Load
Elution
Acquire (Lock_CDII)
Load the waste buffer reservoir and elution collecting plate to Bravo.
Run the Bravo protocol to perform peptide cleanup.
Load the elution plate to the regrip location.
Acquire CombiDancer.

Load the elution plate to CombiDancer.

Dry down peptides by CombiDancer.

Dispose of the eluting buffer reservoir in the trash can.

Load the elution plate to the regrip location.

Dispose of the waste reservoir in the trash can.

Dispose of the waste reservoir in the trash can.

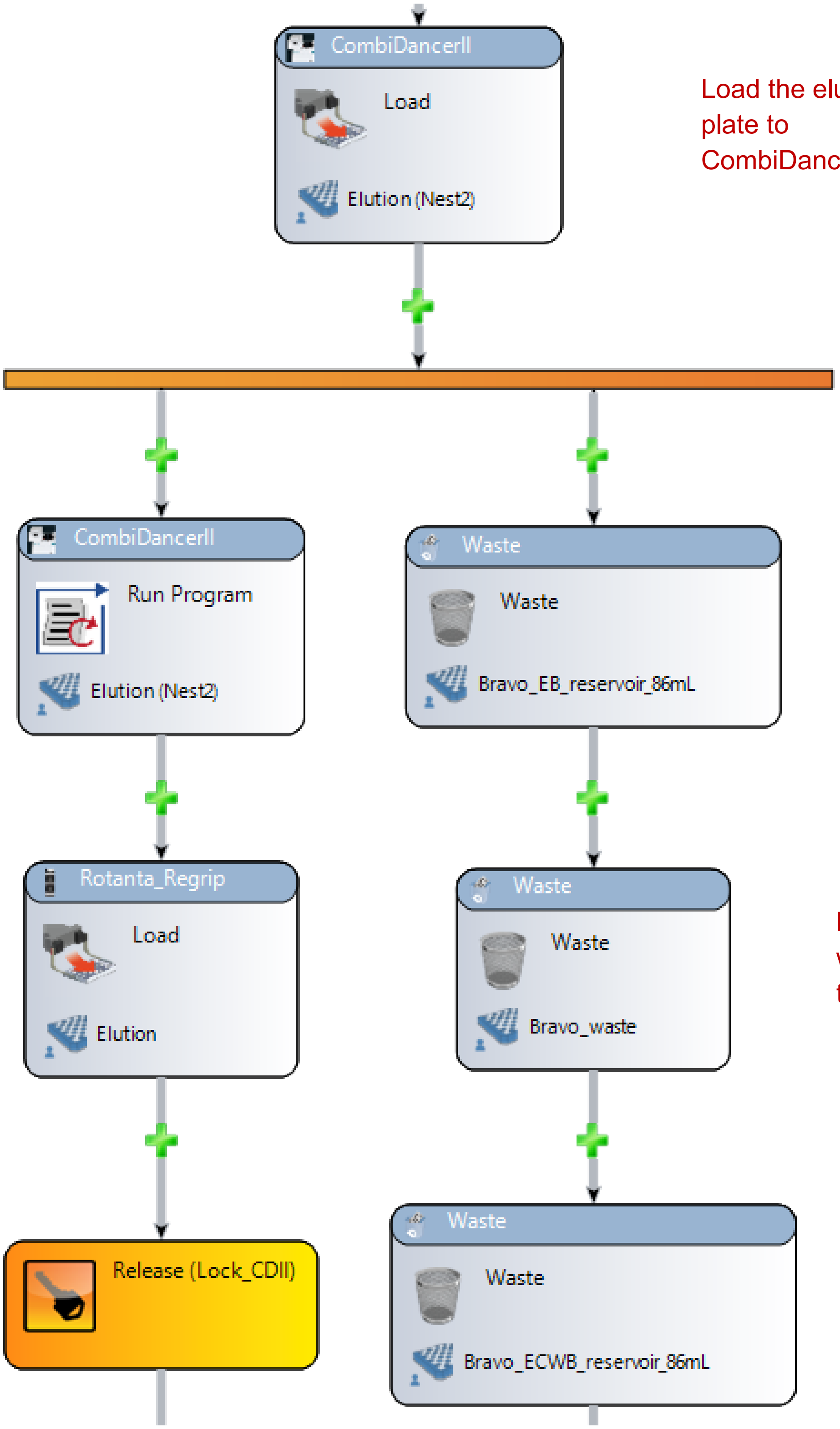

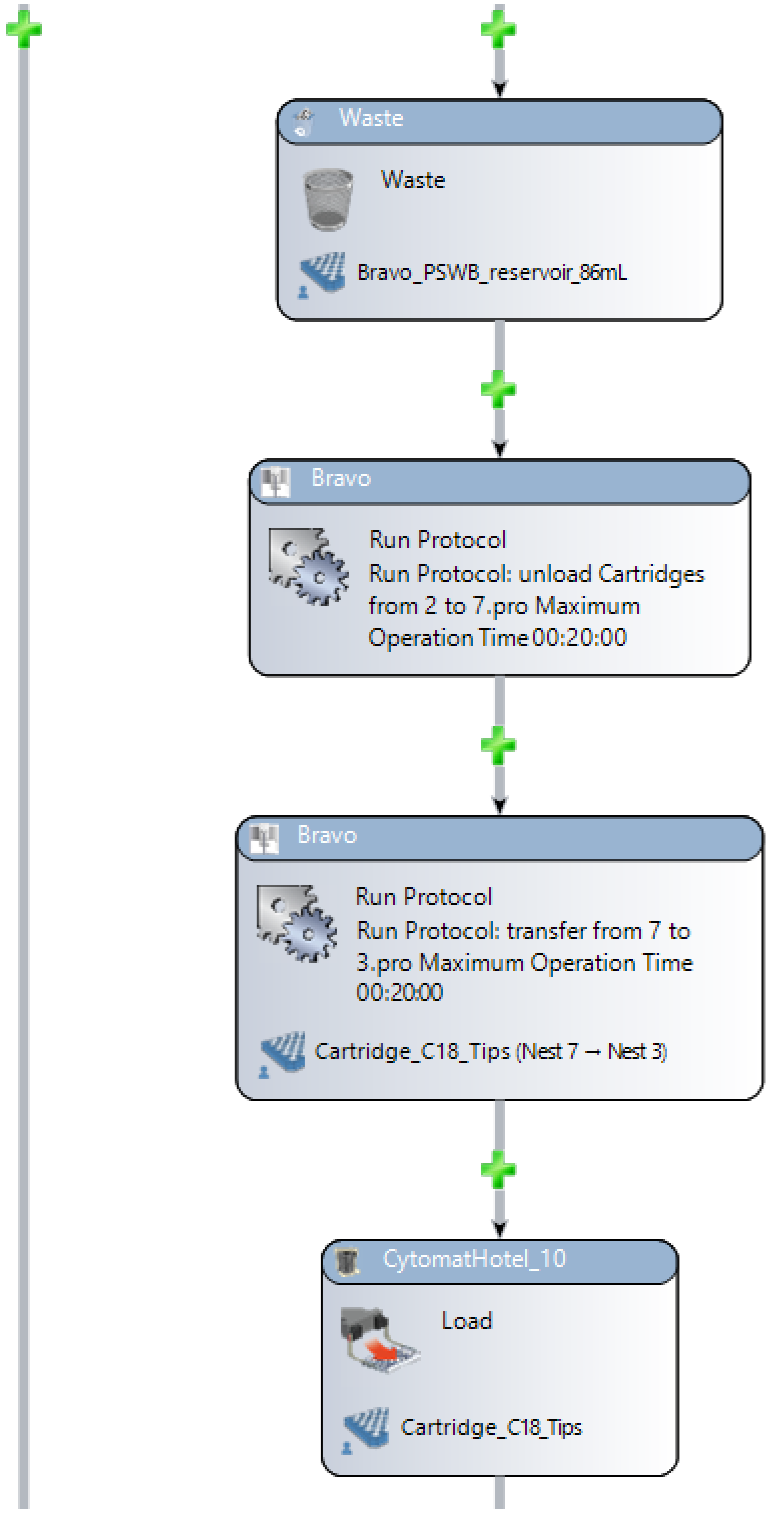

Dispose of the cartridge washing buffer reservoir in the trash can.

Run the Bravo protocol to unload the cartridges to nest 7.

Run the Bravo protocol to transfer the cartridges from nest 7 to nest 3.

Load the C18 cartridges to Cytomat 10 hotel.

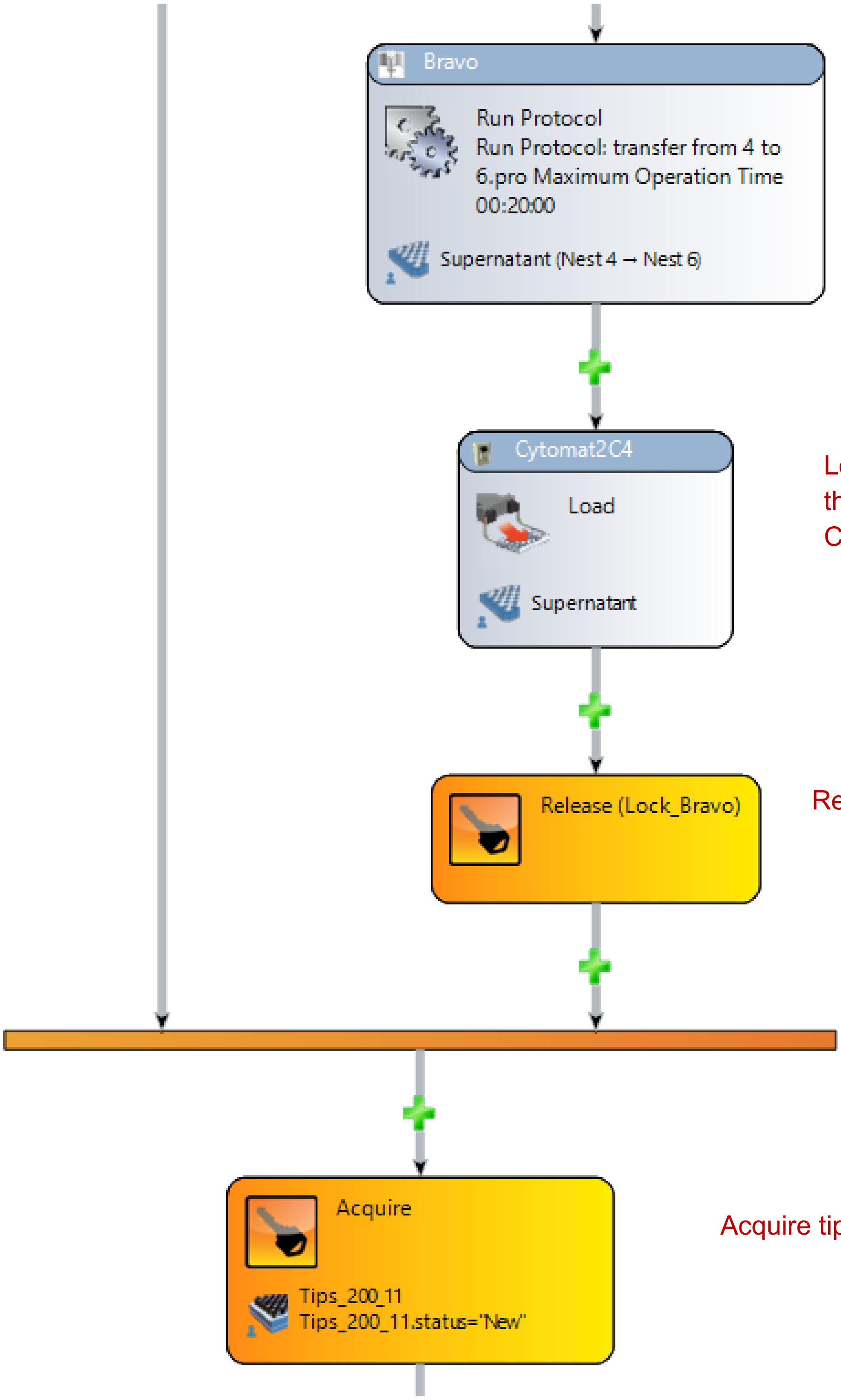

Run the Bravo protocol to transfer the flow-through plate from nest 4 to nest 6.

Load the flow-through plate to Cytomat 2 hotel.

Release Bravo.

Acquire tips.

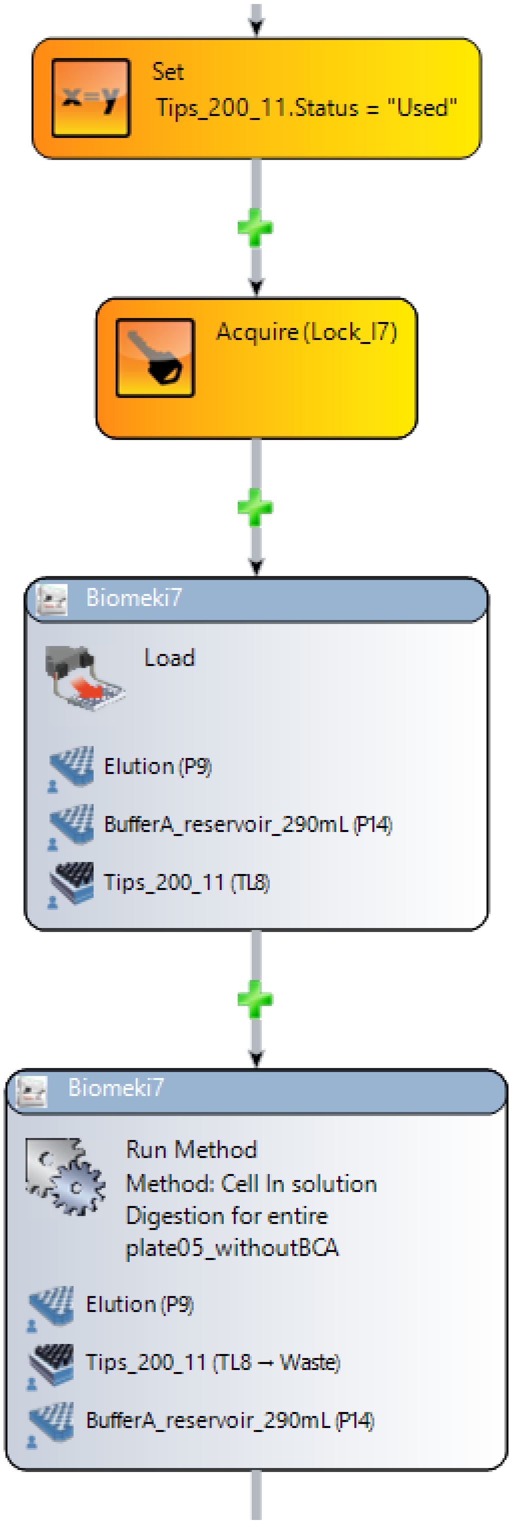

Set the status of tips.

Acquire Biomek i7.

Load tips and buffer to Biomek i7 for preparing peptide solutions.

Dissolve the peptides in 0.1% FA buffer by Biomek i7.

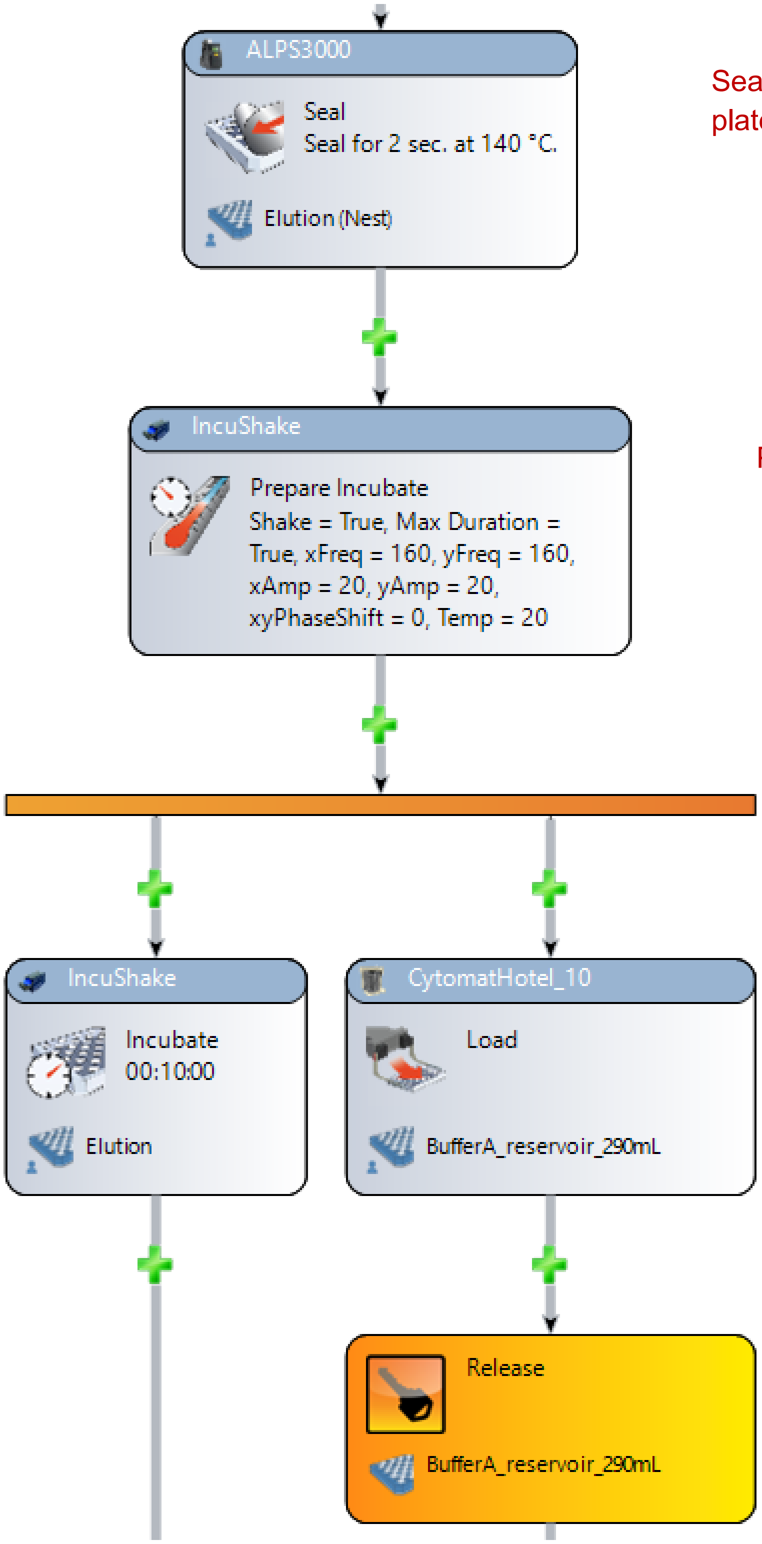

Seal the elution plate by ALPS3000.

Prepare Inheco.

Incubate the elution plate in Inheco to dissolve peptides.

Load the BufferA reservoir (0.1% FA in water) to Cytomat 10 hotel.

Release Buffer A reservoir.

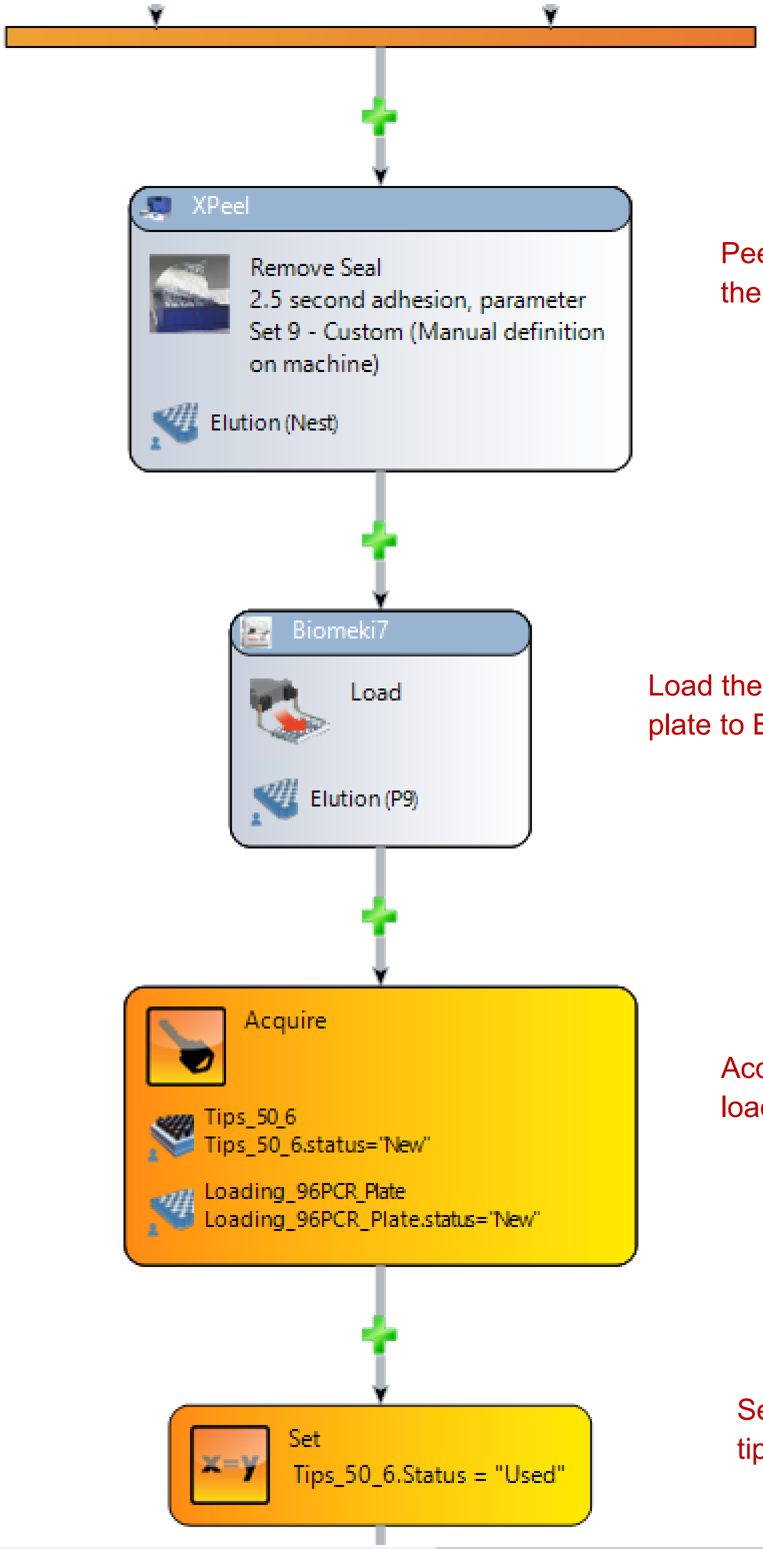

Peel off the film of the elution plate.

Load the elution plate to Biomek i7.

Acquire tips and the loading plate.

Set the status of tips.

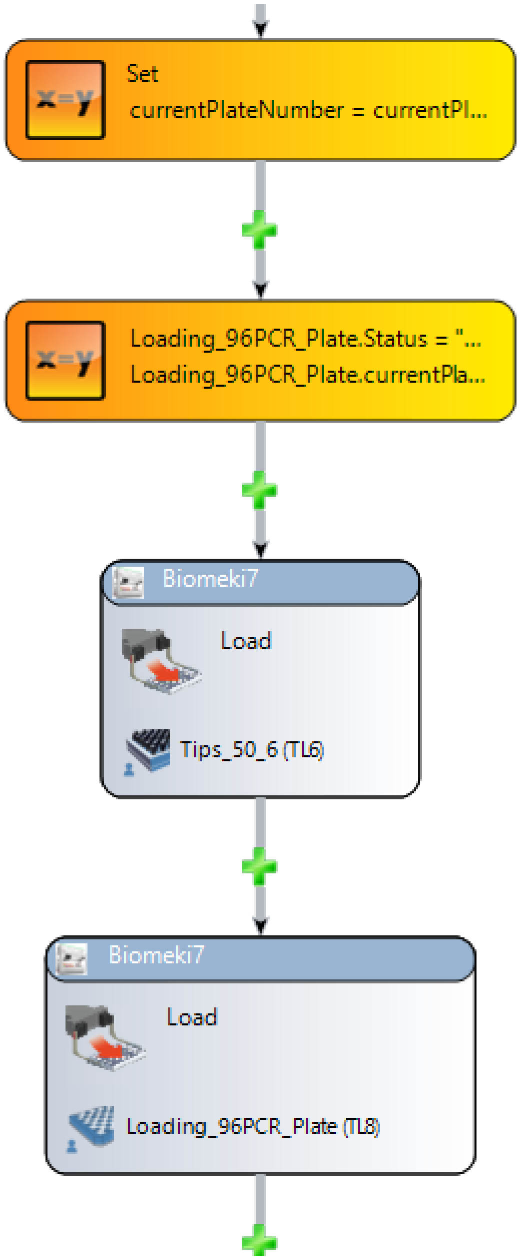

Set the number (ID) of the loading plate.

Set the status of the loading plate.

Load tips to Biomek i7.

Load the loading plate to Biomek i7.

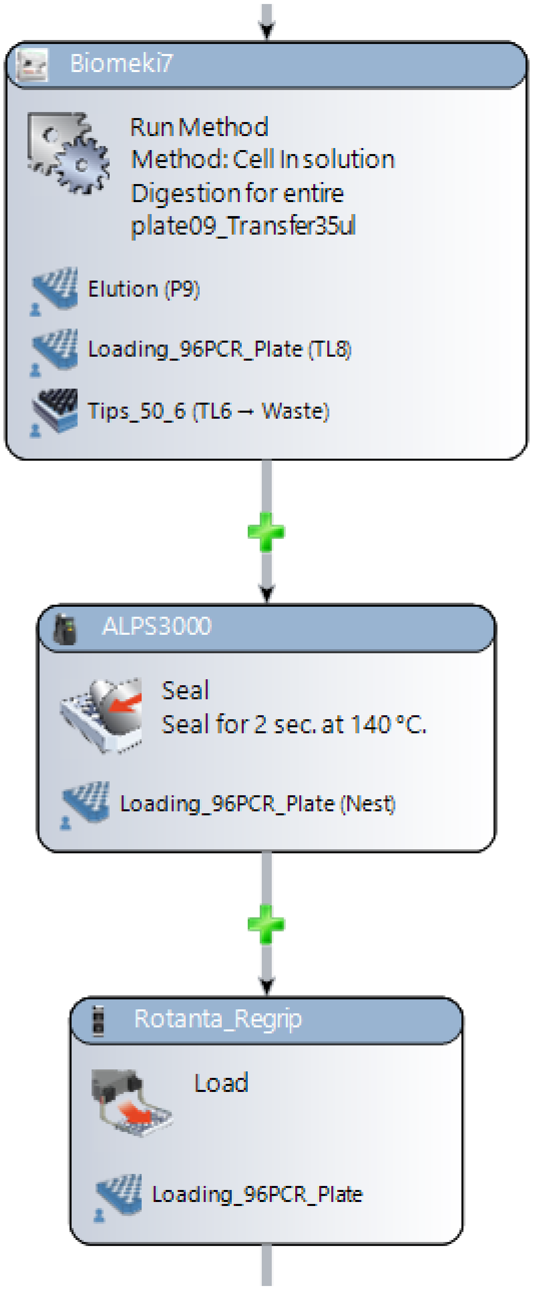

Transfer peptides from the elution plate to the loading plate by Biomek i7.

Seal the loading plate by ALPS3000.

Load the loading plate to the regrip location.

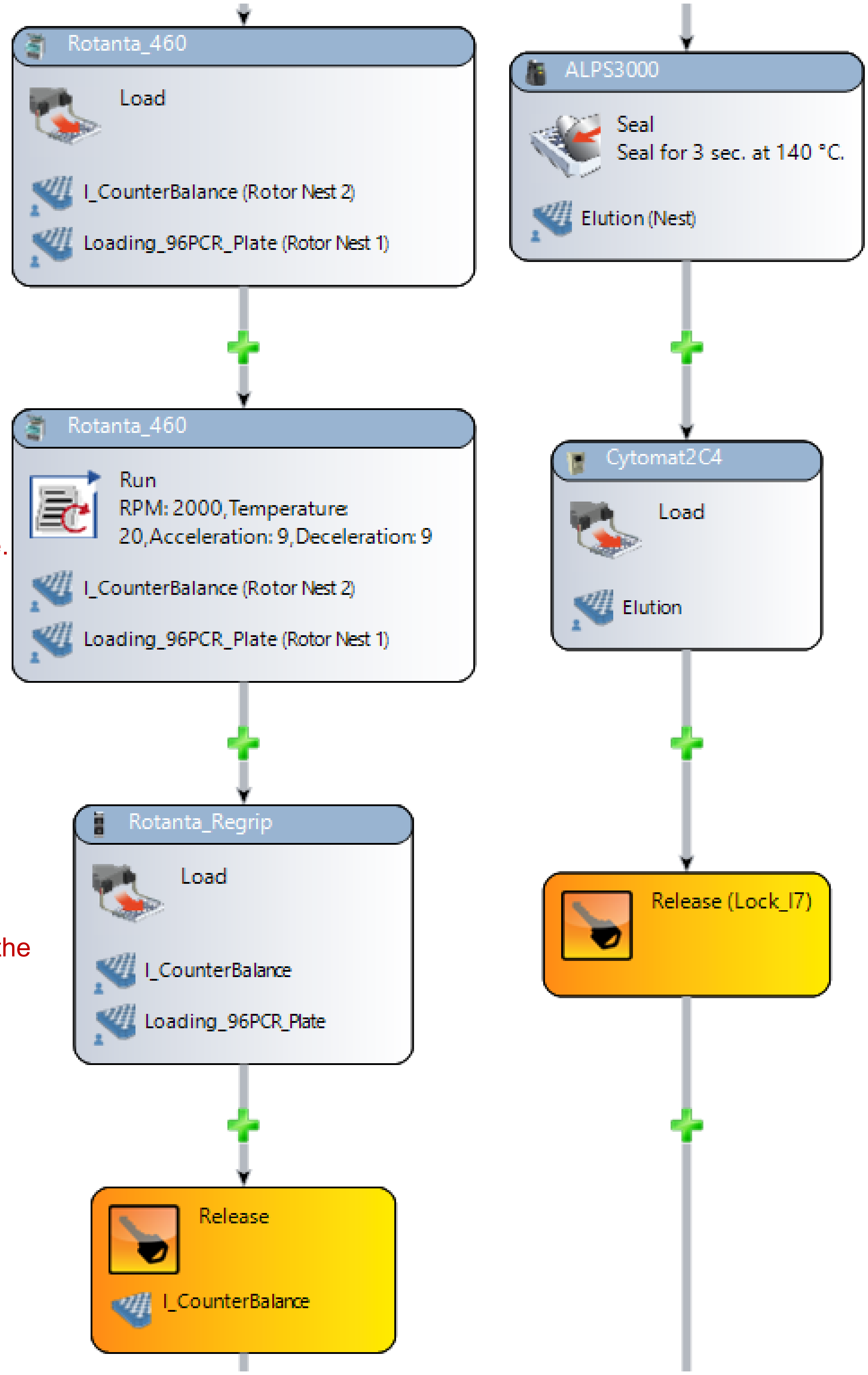

Load the loading plate and the balance plate to Rotanta.

Centrifuge the loading plate and the balance plate.

Load the loading plate and the balance plate to the regrip location.

Release the balance plate.

Seal the elution plate by ALPS3000.

Load the elution plate to Cytomat 2C4 hotel.

Release Biomek i7.

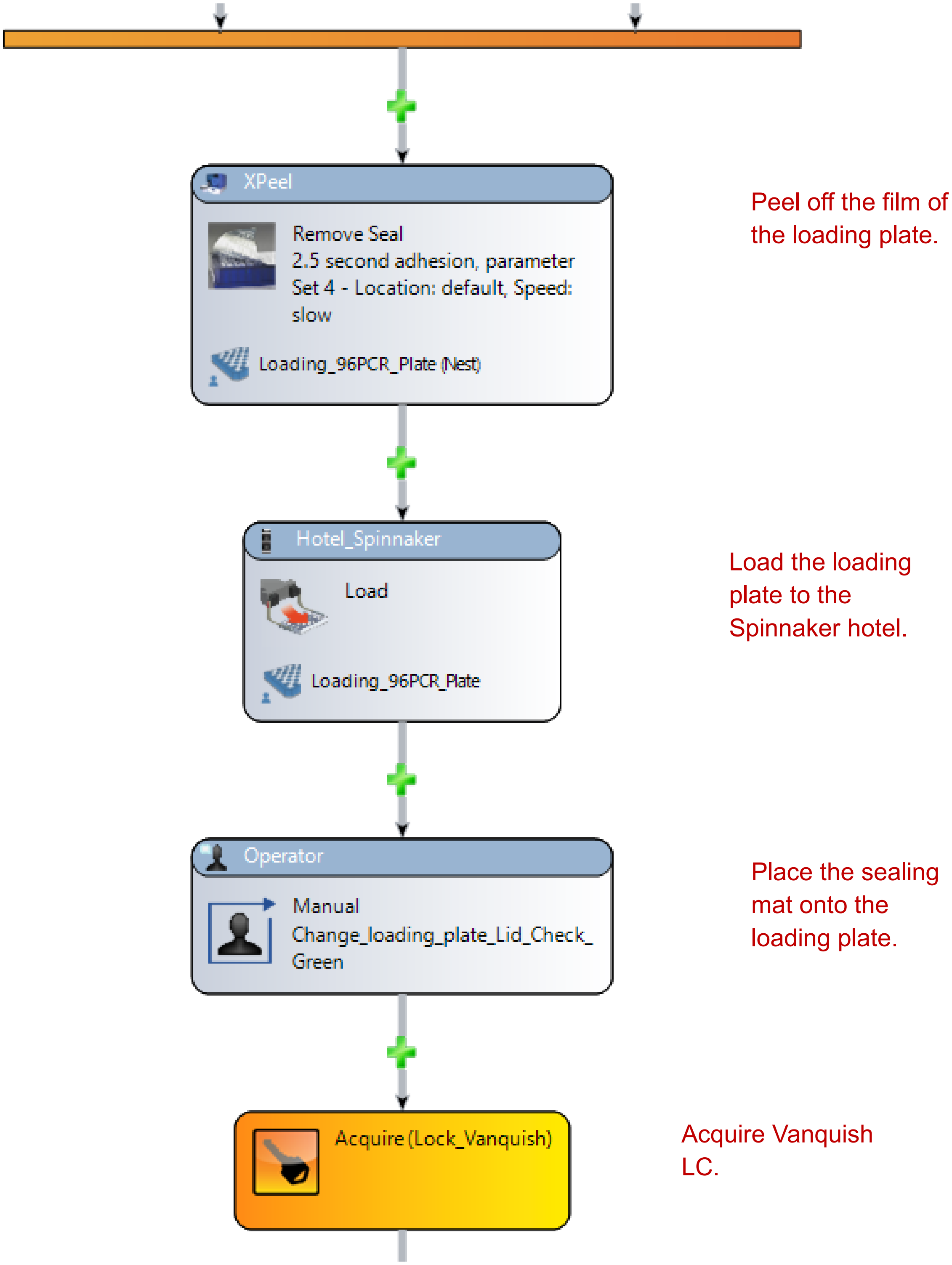
XPeel
Remove Seal
2.5 second adhesion, parameter Set 4 - Location: default, Speed: slow
Loading_96PCR_Plate (Nest)
Peel off the film of the loading plate.
Hotel_Spinnaker
Load
Loading_96PCR_Plate
Load the loading plate to the Spinnaker hotel.
Operator
Manual
Change_loading_plate_Lid_Check_Green
Place the sealing mat onto the loading plate.
Acquire (Lock_Vanquish)
Acquire Vanquish LC.

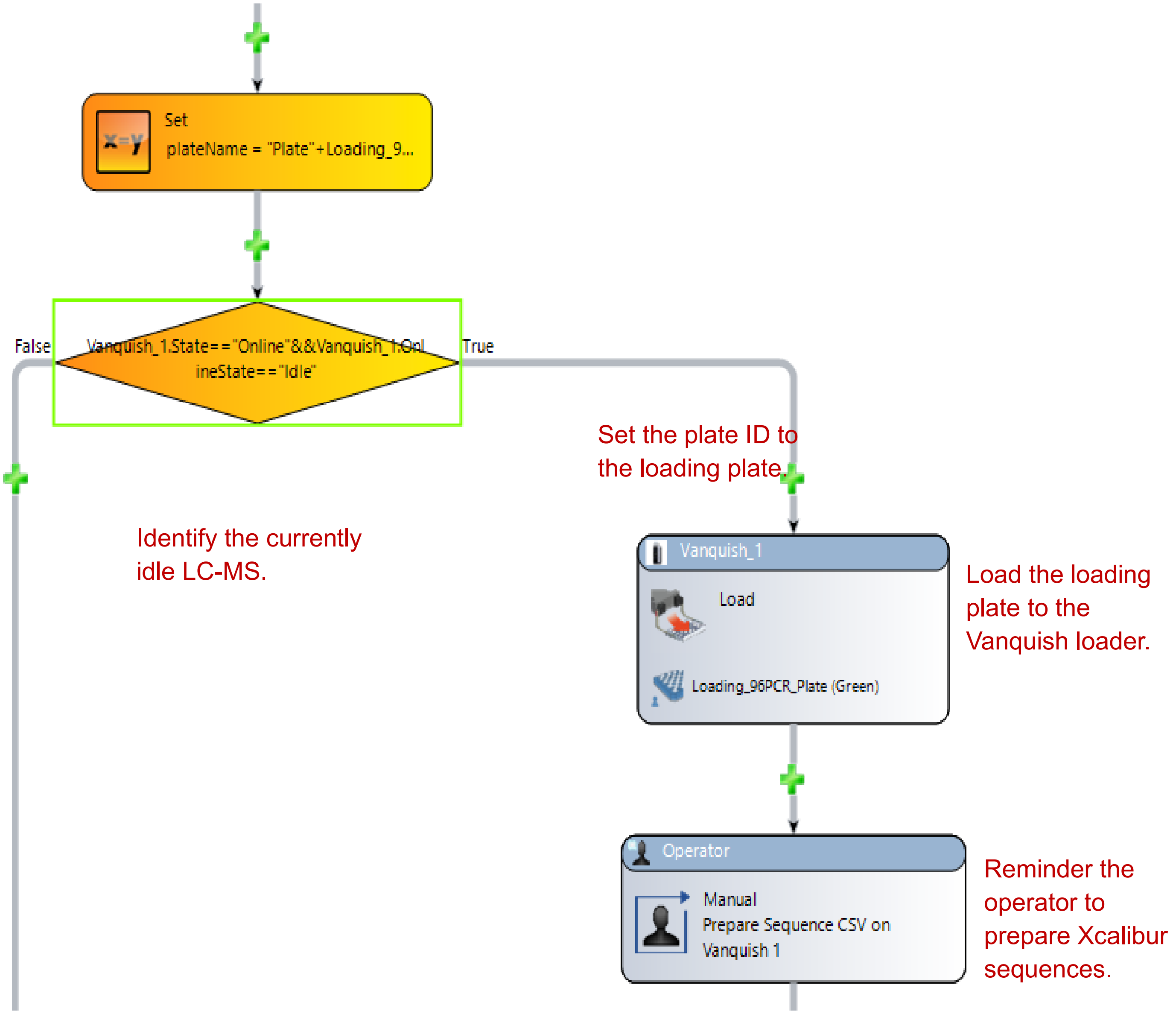
Set
plateName = "Plate"+Loading_9...
False
Vanquish_1.State=="Online"&&Vanquish_1.OnlineState=="Idle"
True
Set the plate ID to the loading plate.
Identify the currently idle LC-MS.
Vanquish_1
Load
Loading_96PCR_Plate (Green)
Load the loading plate to the Vanquish loader.
Operator
Manual
Prepare Sequence CSV on Vanquish 1
Reminder the operator to prepare Xcalibur sequences.

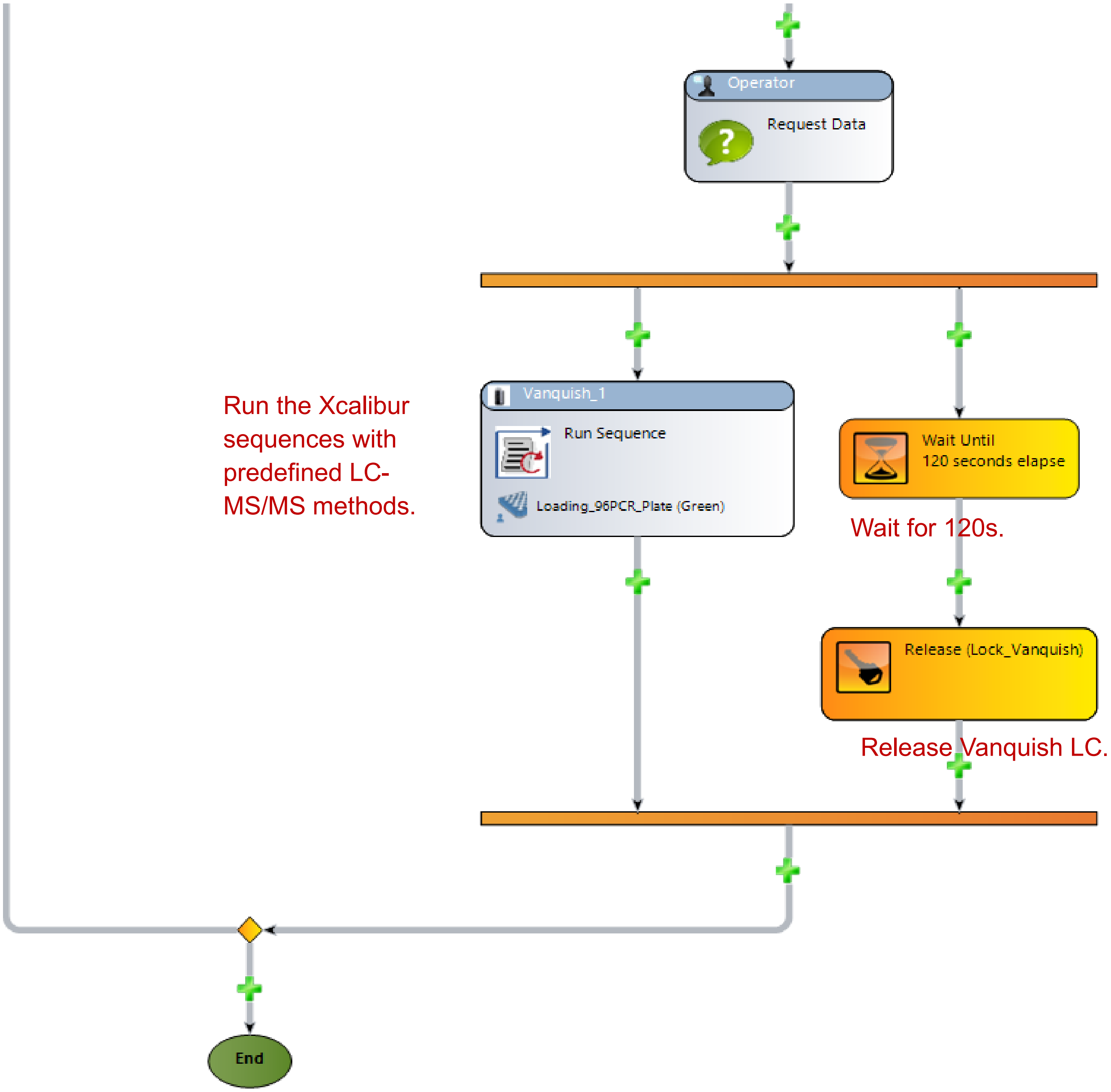
Operator
Request Data
Run the Xcalibur sequences with predefined LC-MS/MS methods.
Vanquish_1
Run Sequence
Loading_96PCR_Plate (Green)
Wait Until
120 seconds elapse
Wait for 120s.
Release (Lock_Vanquish)
Release Vanquish LC.
End

3. The process for generating plasma proteome data

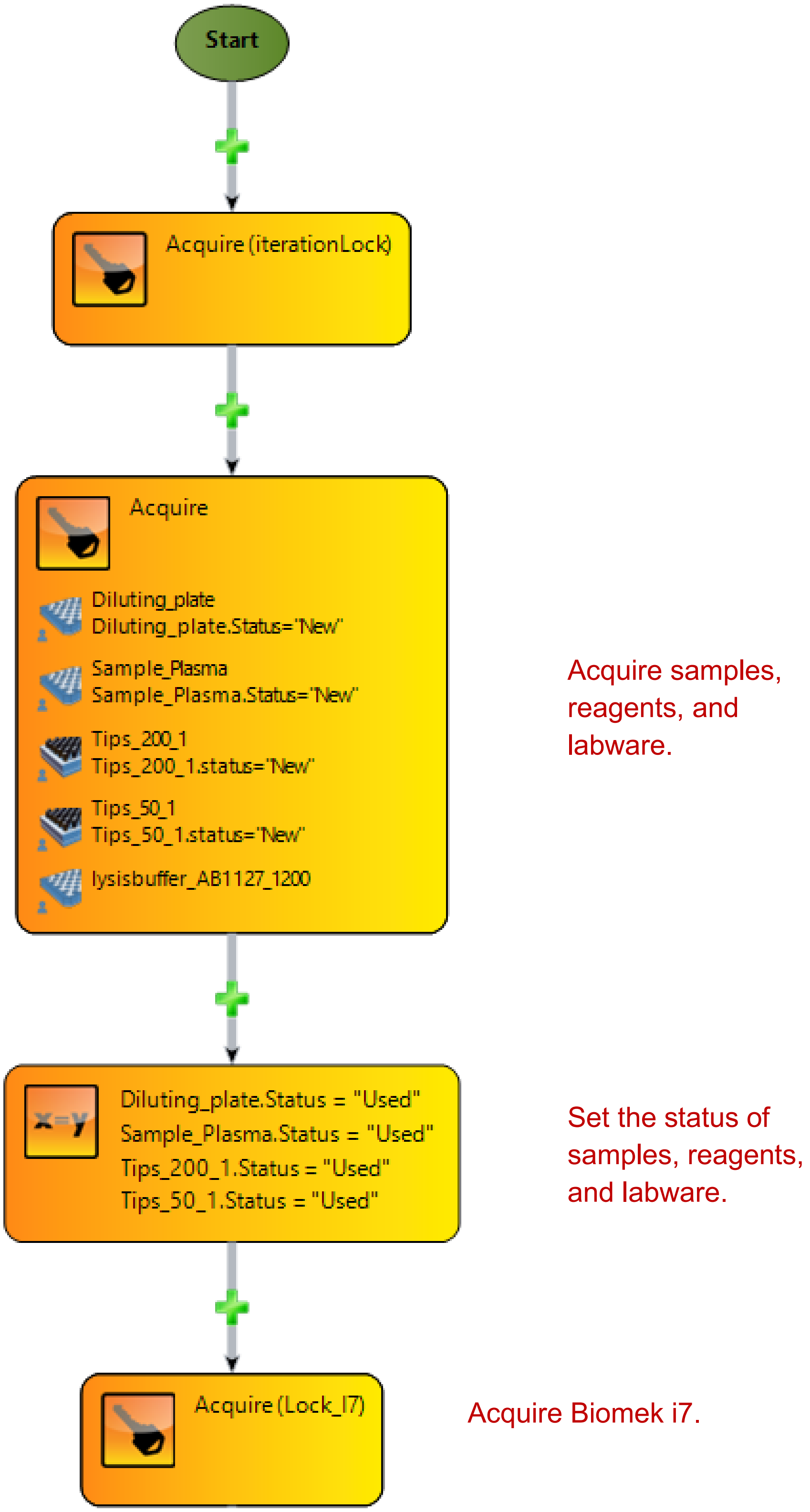

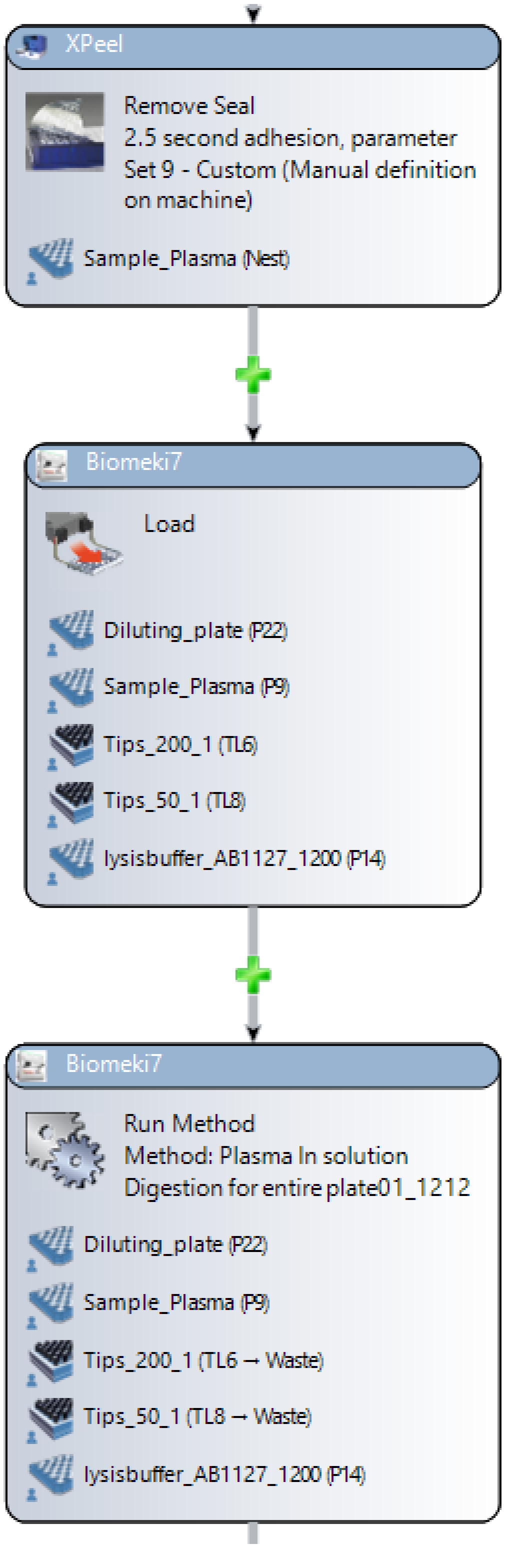

Peel off the film of the sample plate.

Load the sample plates, diluting buffer, diluting plate, and tips to Biomek i7.

Dilute the plasma samples by adding lysis buffer by Biomek i7.

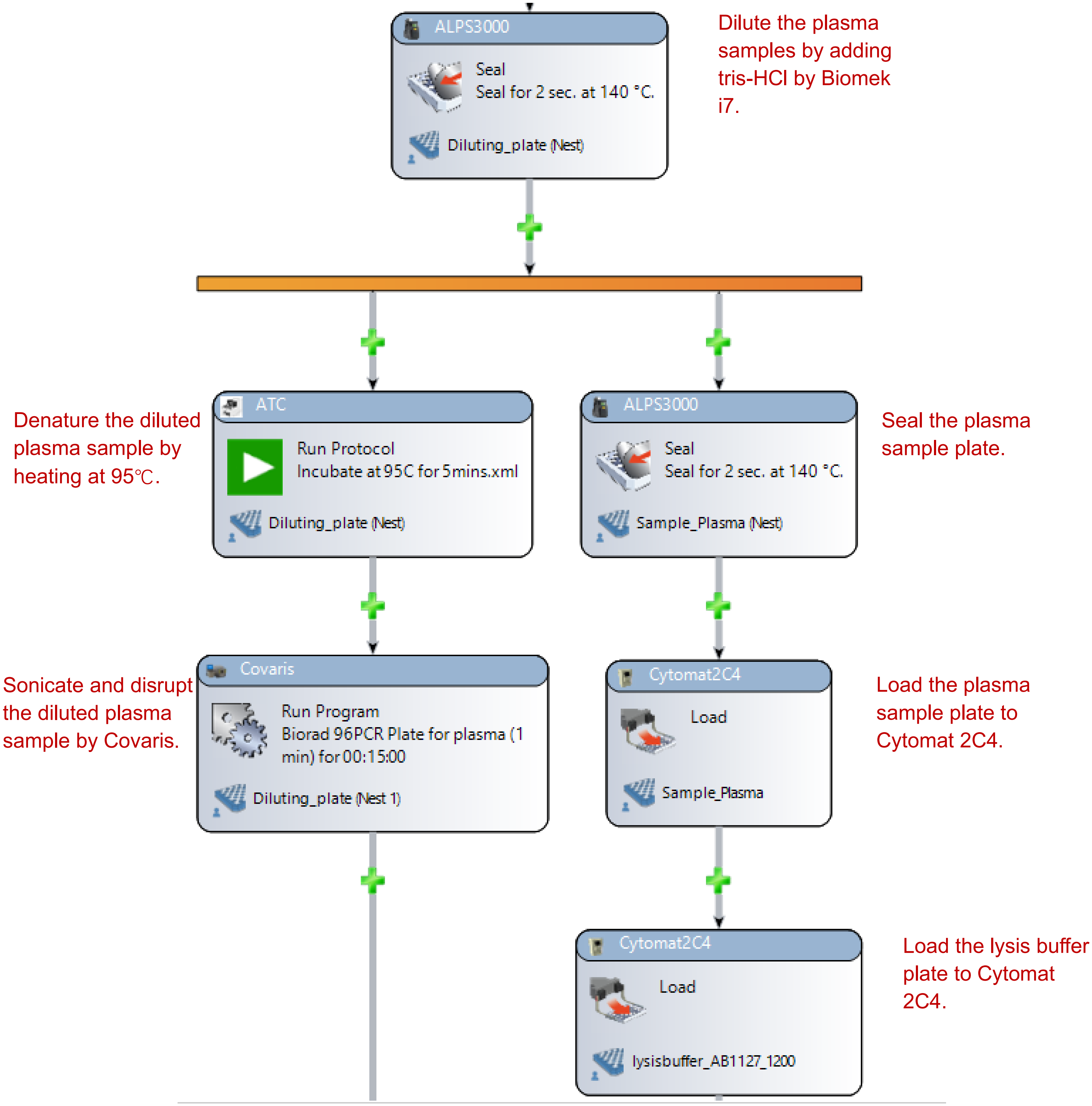
ALPS3000
Seal
Seal for 2 sec. at 140 °C.
Diluting_plate (Nest)
Dilute the plasma samples by adding tris-HCl by Biomek i7.
ATC
Run Protocol
Incubate at 95C for 5mins.xml
Diluting_plate (Nest)
Denature the diluted plasma sample by heating at 95℃.
ALPS3000
Seal
Seal for 2 sec. at 140 °C.
Sample_Plasma (Nest)
Seal the plasma sample plate.
Covaris
Run Program
Biorad 96PCR Plate for plasma (1 min) for 00:15:00
Diluting_plate (Nest 1)
Sonicate and disrupt the diluted plasma sample by Covaris.
Cytomat2C4
Load
Sample_Plasma
Load the plasma sample plate to Cytomat 2C4.
Cytomat2C4
Load
lysisbuffer_AB1127_1200
Load the lysis buffer plate to Cytomat 2C4.

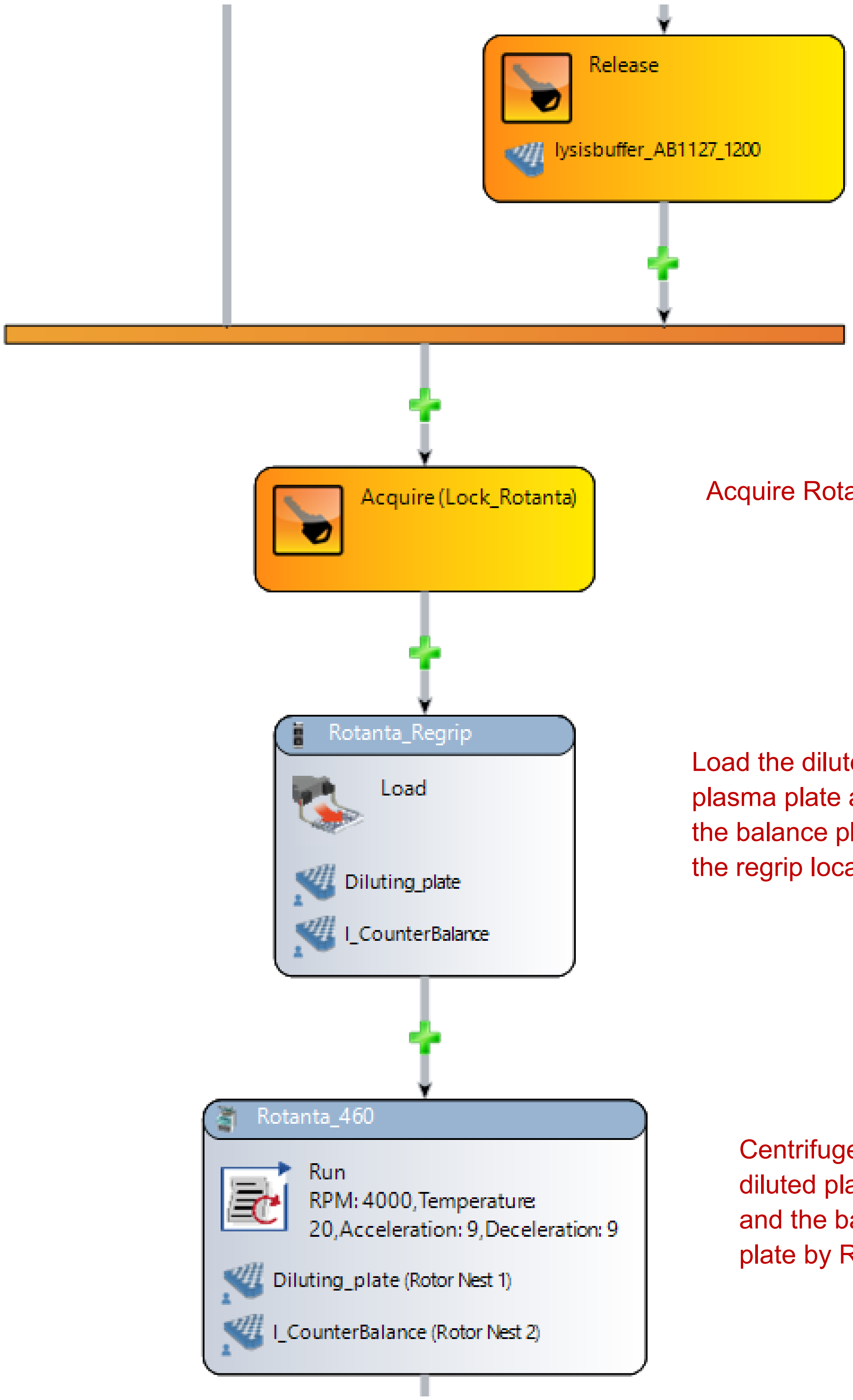

Release the lysis buffer.

Acquire Rotanta.

Load the diluted plasma plate and the balance plate to the regrip location.

Centrifuge the diluted plasma plate and the balance plate by Rotanta.

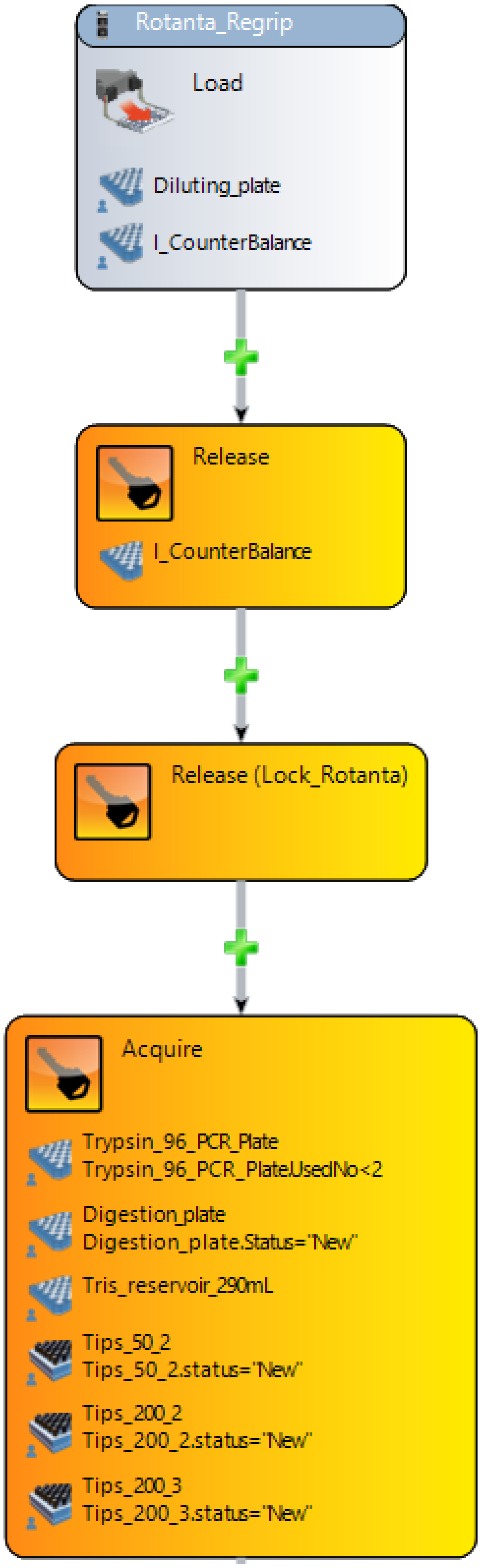

Load the diluted plasma plate and the balance plate to the regrip location.

Release the balance plate.

Release Rotanta.

Acquire plates, tips, and reagents.

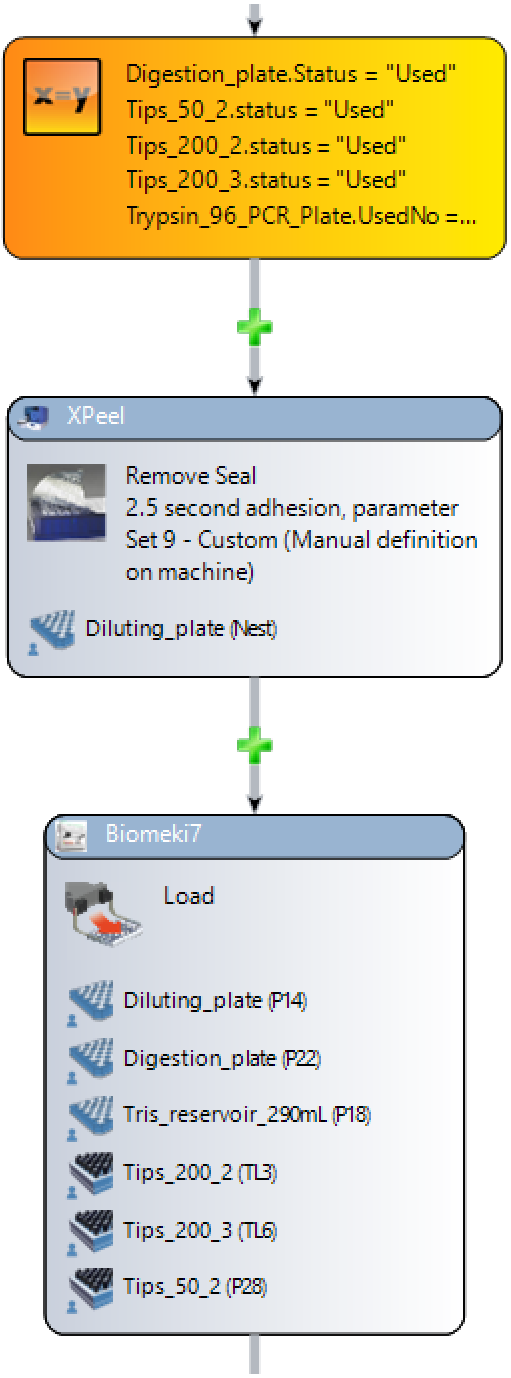

Set the status of plates, tips, and reagents.

Peel off the film of the diluted plasma plate.

Load plates, tips, buffers, and the diluted plasma to Biomek i7.

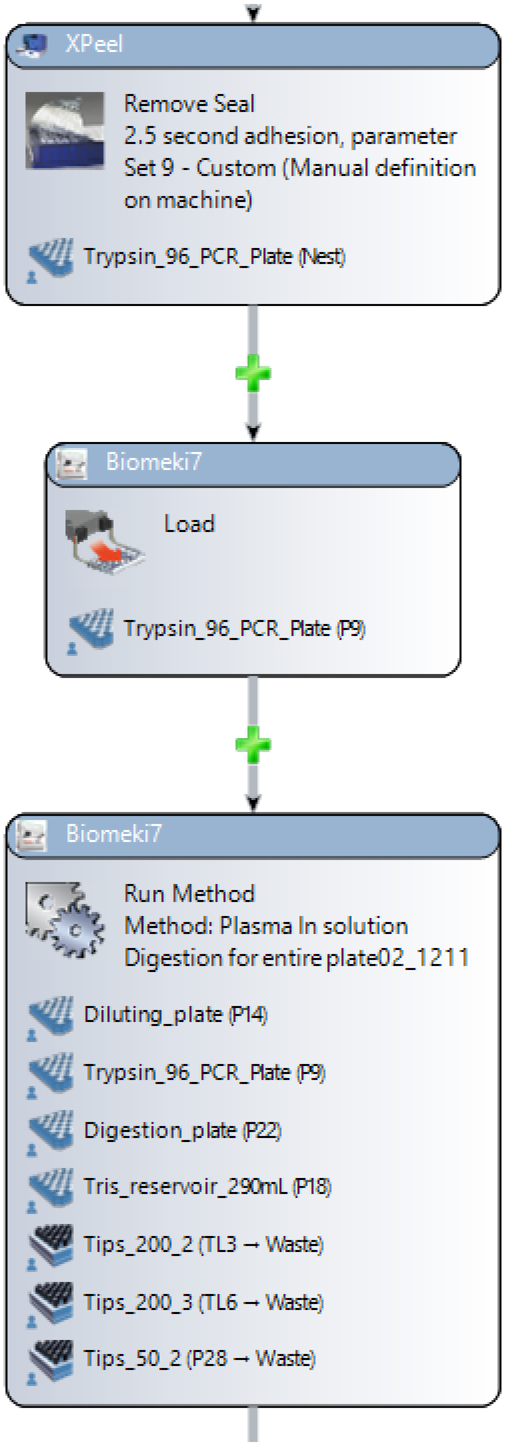

Peel off the film of the trypsin plate.

Load the trypsin plate to Biomek i7.

Add trypsin to the plasma samples by Biomek i7.

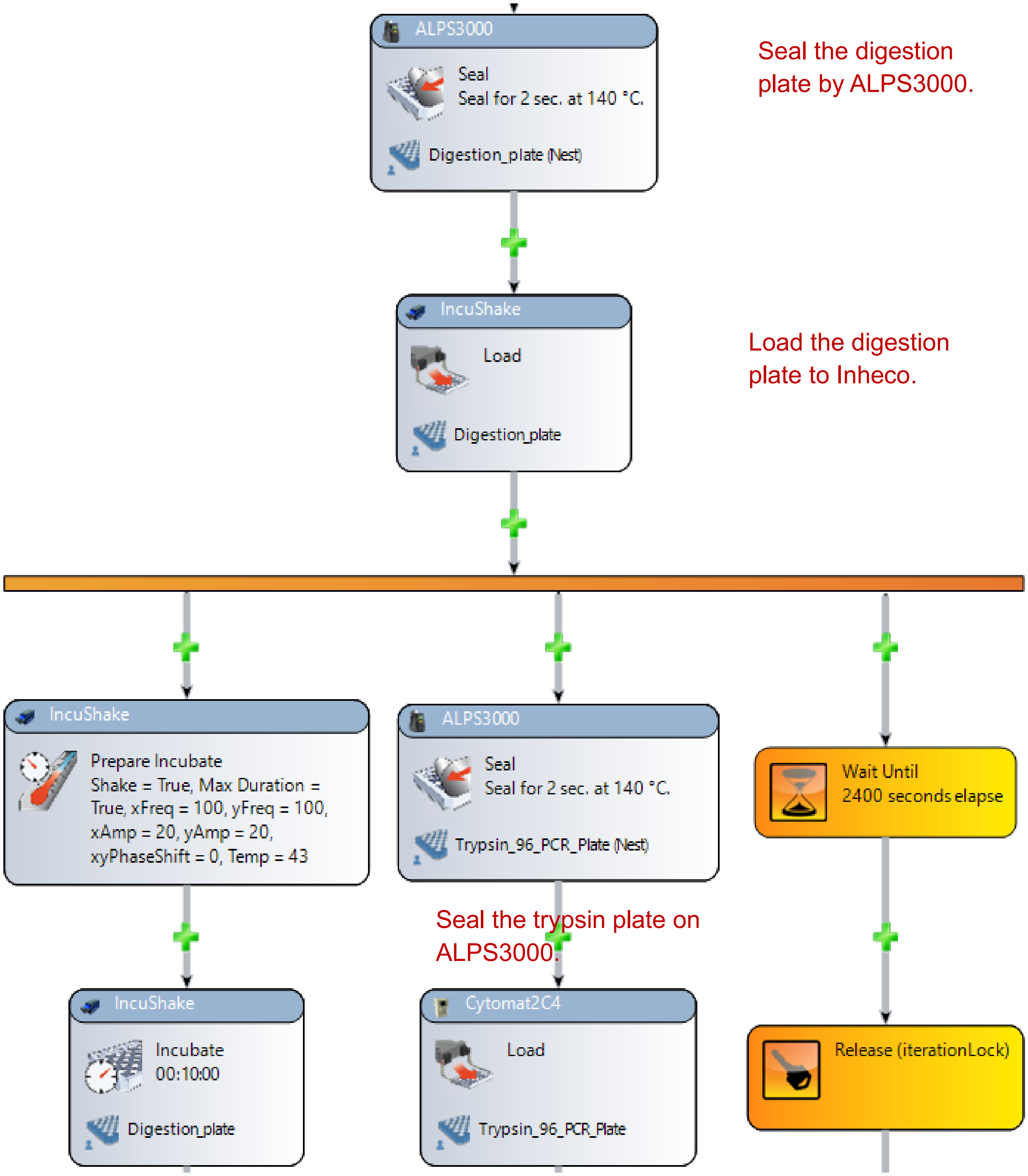

Seal the digestion plate by ALPS3000.

Load the digestion plate to Inheco.

Set Inheco to 43 ℃.

Seal the trypsin plate on ALPS3000.

Incubate the digestion plate at 43℃ for 10min.

Here is for pre-heat.

Load the trypsin plate to Cytomat 2C4 for storage.

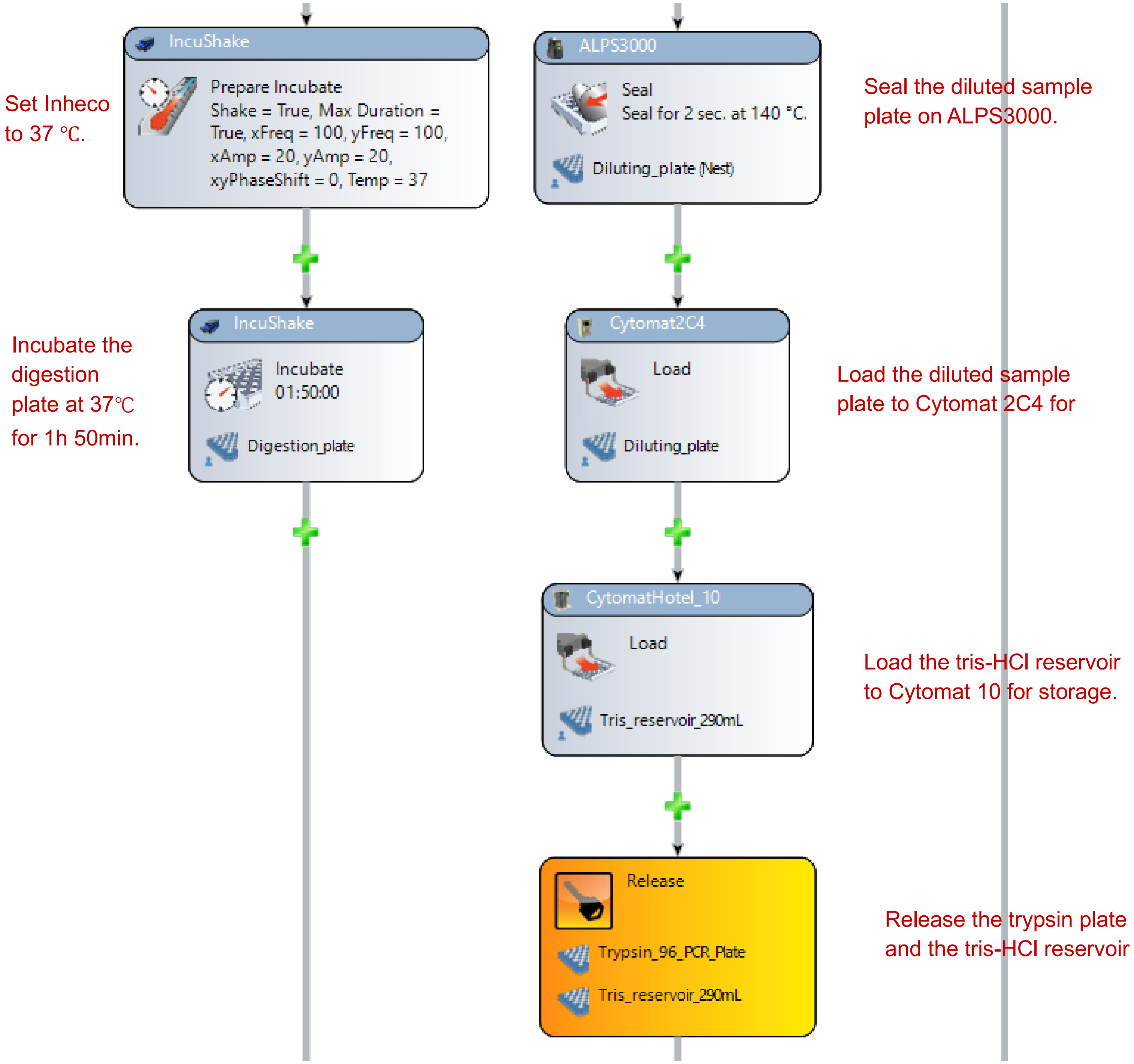
Set Inheco to 37 ℃.
IncuShake
Prepare Incubate
Shake = True, Max Duration = True, xFreq = 100, yFreq = 100, xAmp = 20, yAmp = 20, xyPhaseShift = 0, Temp = 37
ALPS3000
Seal
Seal for 2 sec. at 140 °C.
Diluting_plate (Nest)
Seal the diluted sample plate on ALPS3000.
Incubate the digestion plate at 37℃ for 1h 50min.
IncuShake
Incubate
01:50:00
Digestion_plate
Cytomat2C4
Load
Diluting_plate
Load the diluted sample plate to Cytomat 2C4 for
CytomatHotel_10
Load
Tris_reservoir_290mL
Load the tris-HCl reservoir to Cytomat 10 for storage.
Release
Trypsin_96_PCR_Plate
Tris_reservoir_290mL
Release the trypsin plate and the tris-HCl reservoir

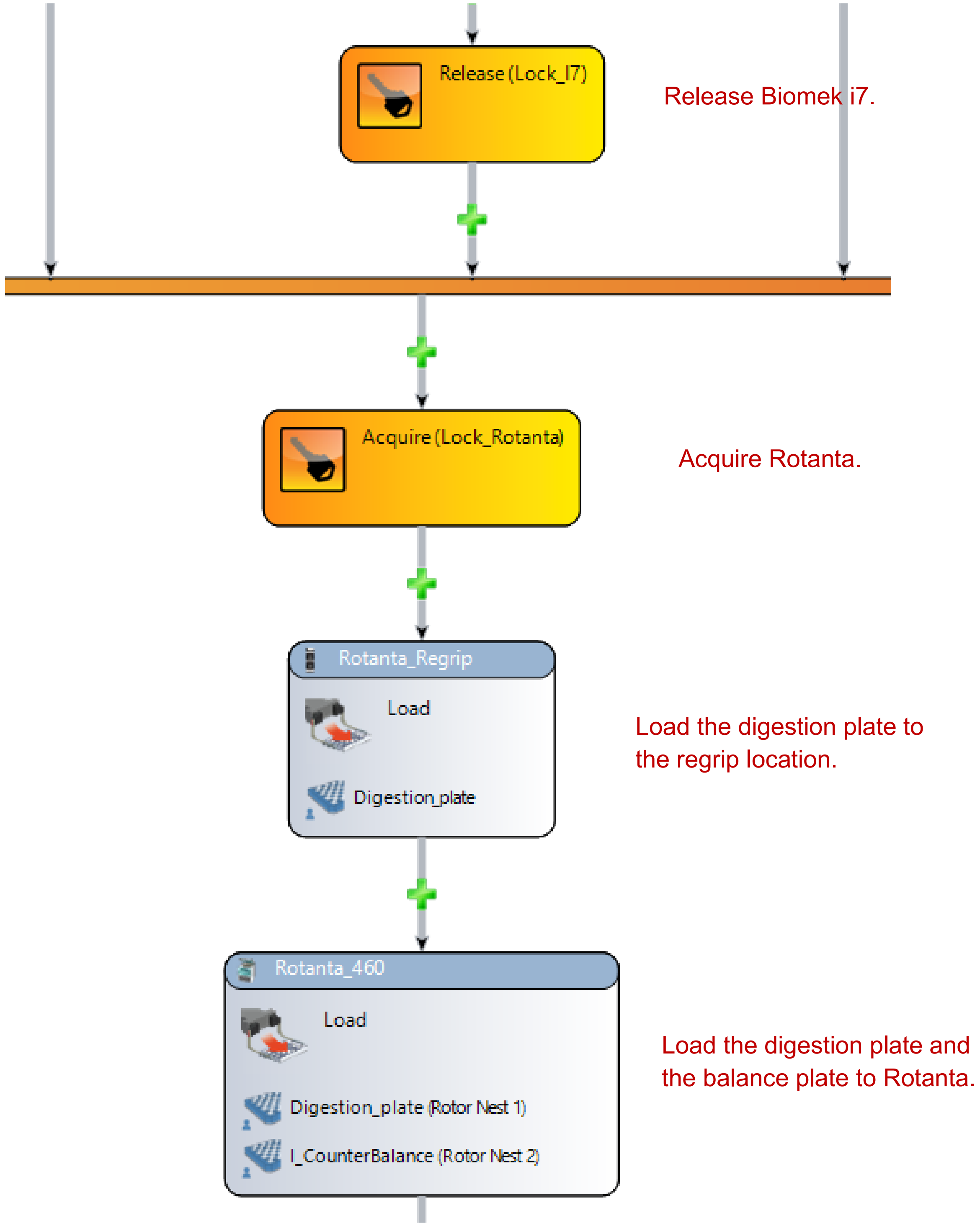
Release (Lock_I7)
Release Biomek i7.
Acquire (Lock_Rotanta)
Acquire Rotanta.
Rotanta_Regrip
Load
Digestion_plate
Load the digestion plate to the regrip location.
Rotanta_460
Load
Digestion_plate (Rotor Nest 1)
I_CounterBalance (Rotor Nest 2)
Load the digestion plate and the balance plate to Rotanta.

Centrifuge the digestion plate by Rotanta.

Take the digestion plate and the balance plate out of Rotanta. Load them to the regrip location.

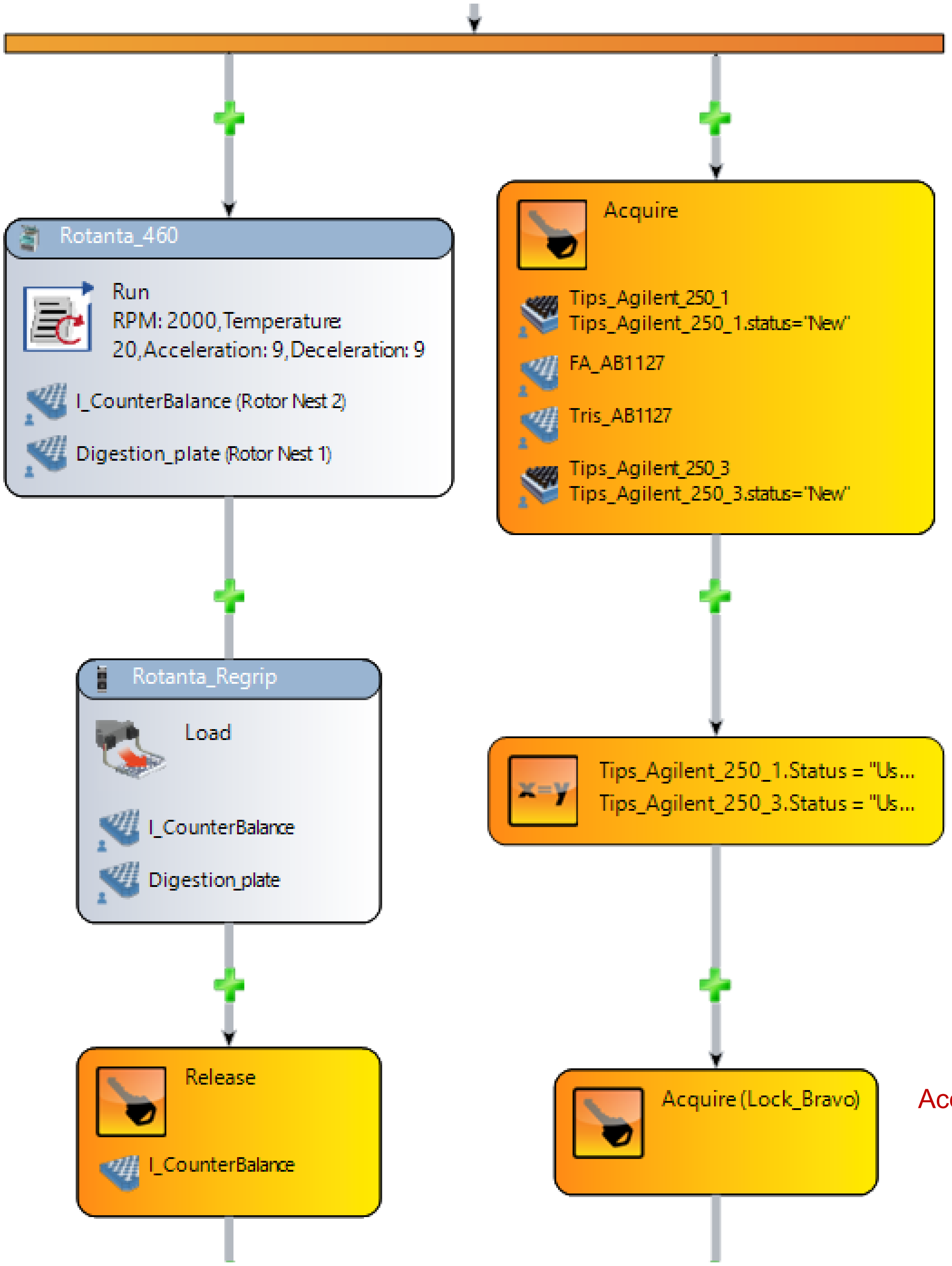

Acquire the reagents and labware.

Set the status of tips.

Acquire Bravo.

Release Rotanta.

Load tips to Bravo.

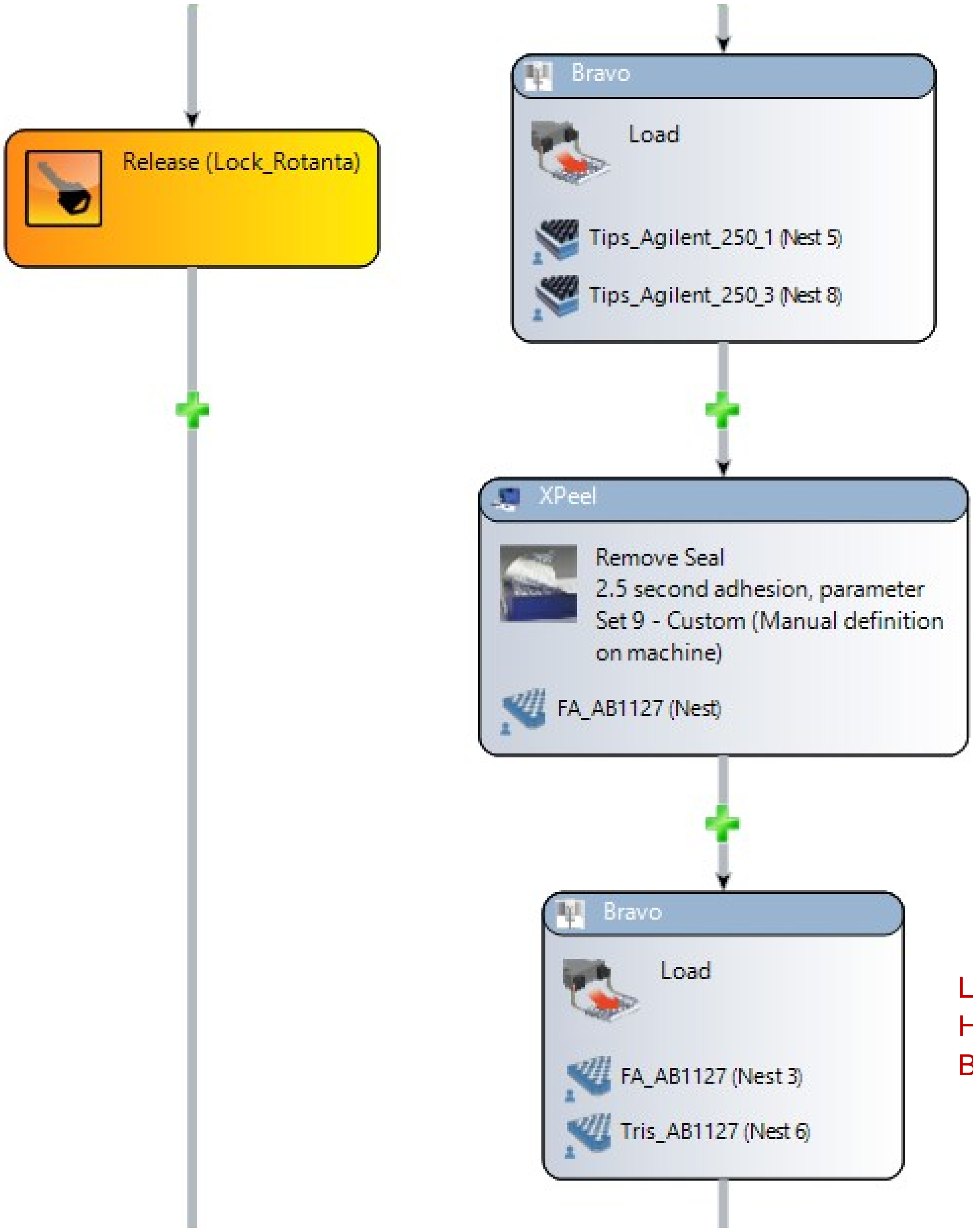

Peel off the film of the Formic acid (FA) reservoir.

Load FA and tris-HCl reservoirs to Bravo.

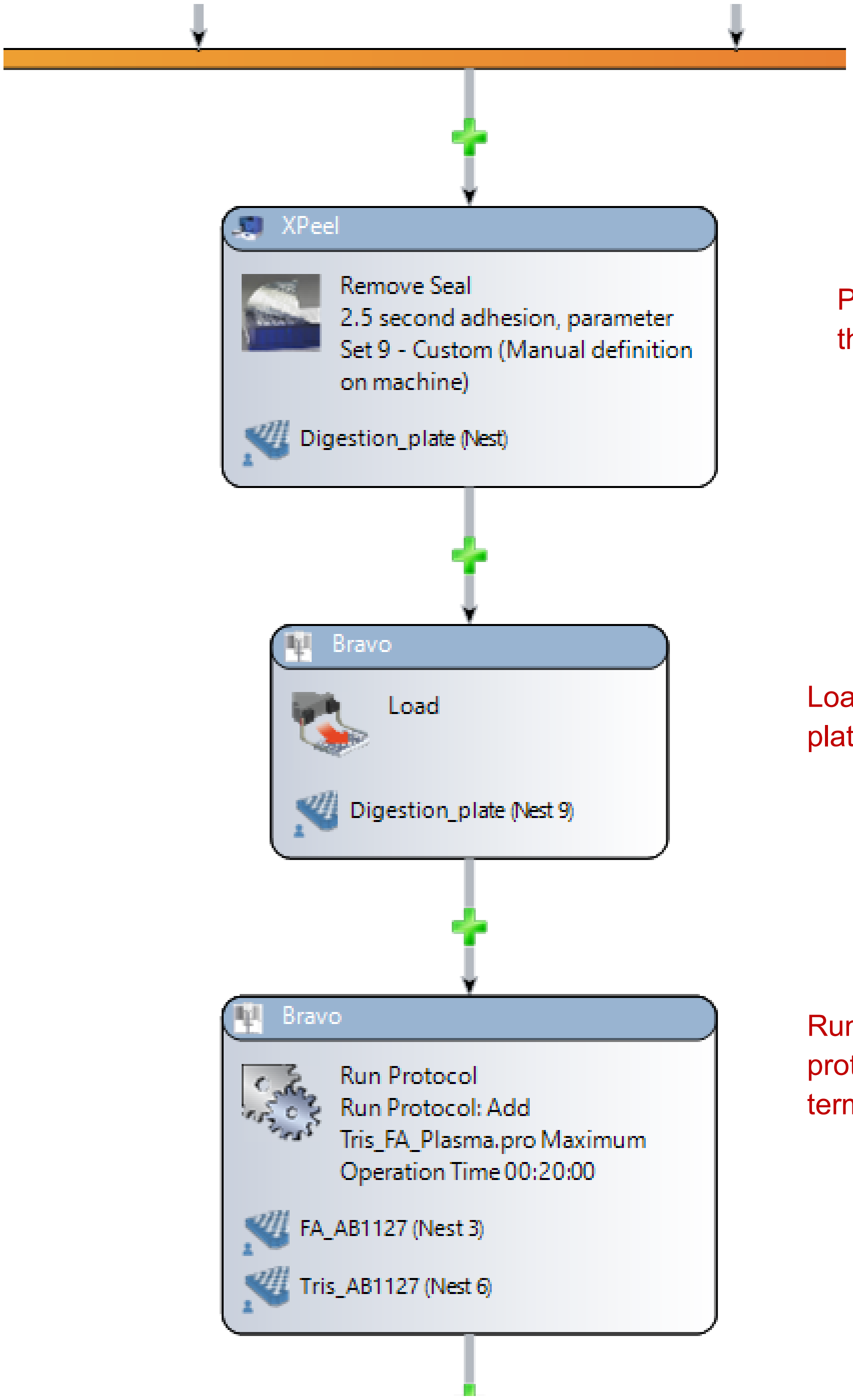

Peel off the film of the digestion plate.

Load the digestion plate to Bravo.

Run the Bravo protocol for terminating digestion.

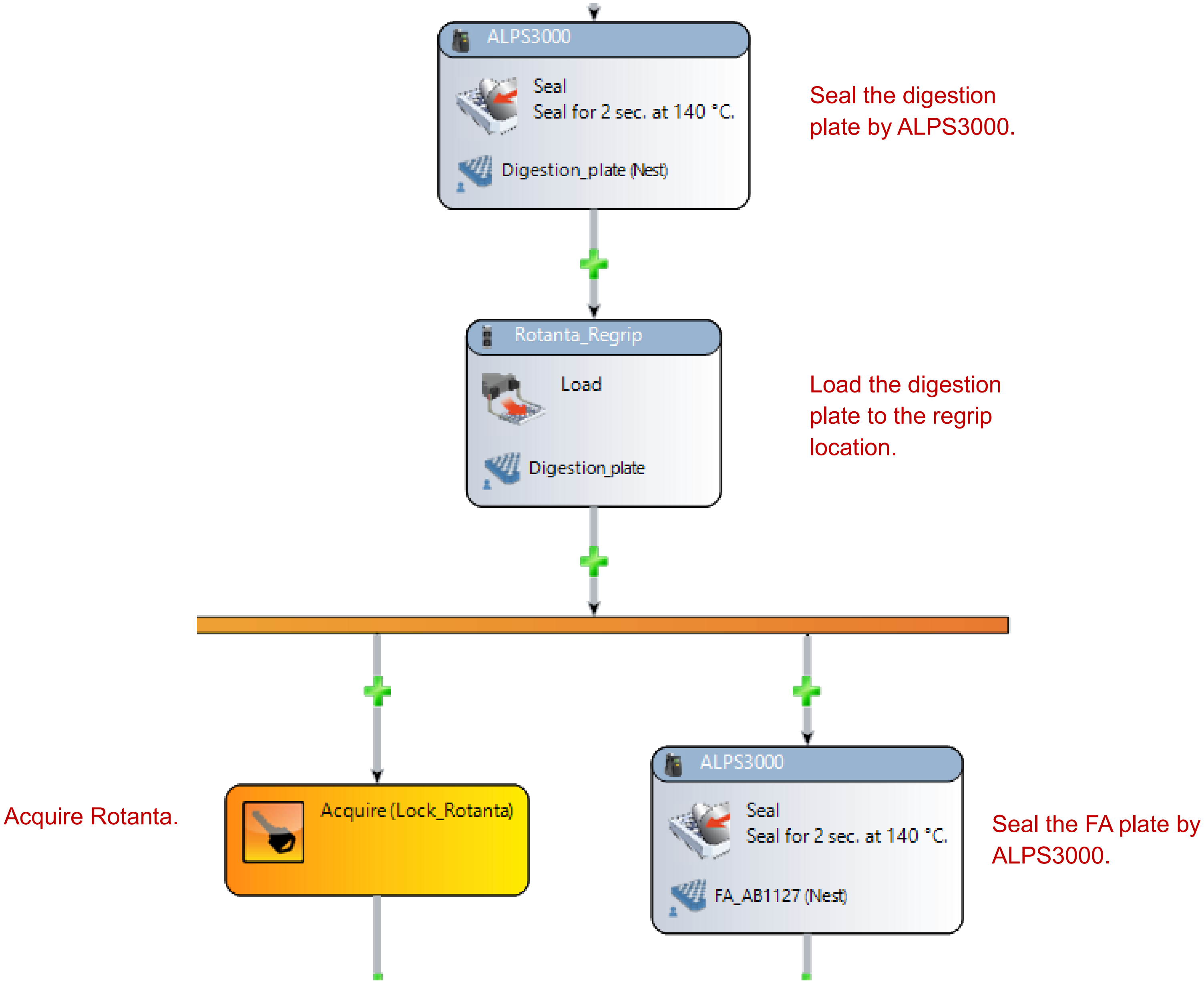
ALPS3000
Seal
Seal for 2 sec. at 140 °C.
Digestion_plate (Nest)
Seal the digestion plate by ALPS3000.
Rotanta_Regrip
Load
Digestion_plate
Load the digestion plate to the regrip location.
Acquire Rotanta.
Acquire (Lock_Rotanta)
ALPS3000
Seal
Seal for 2 sec. at 140 °C.
FA_AB1127 (Nest)
Seal the FA plate by ALPS3000.

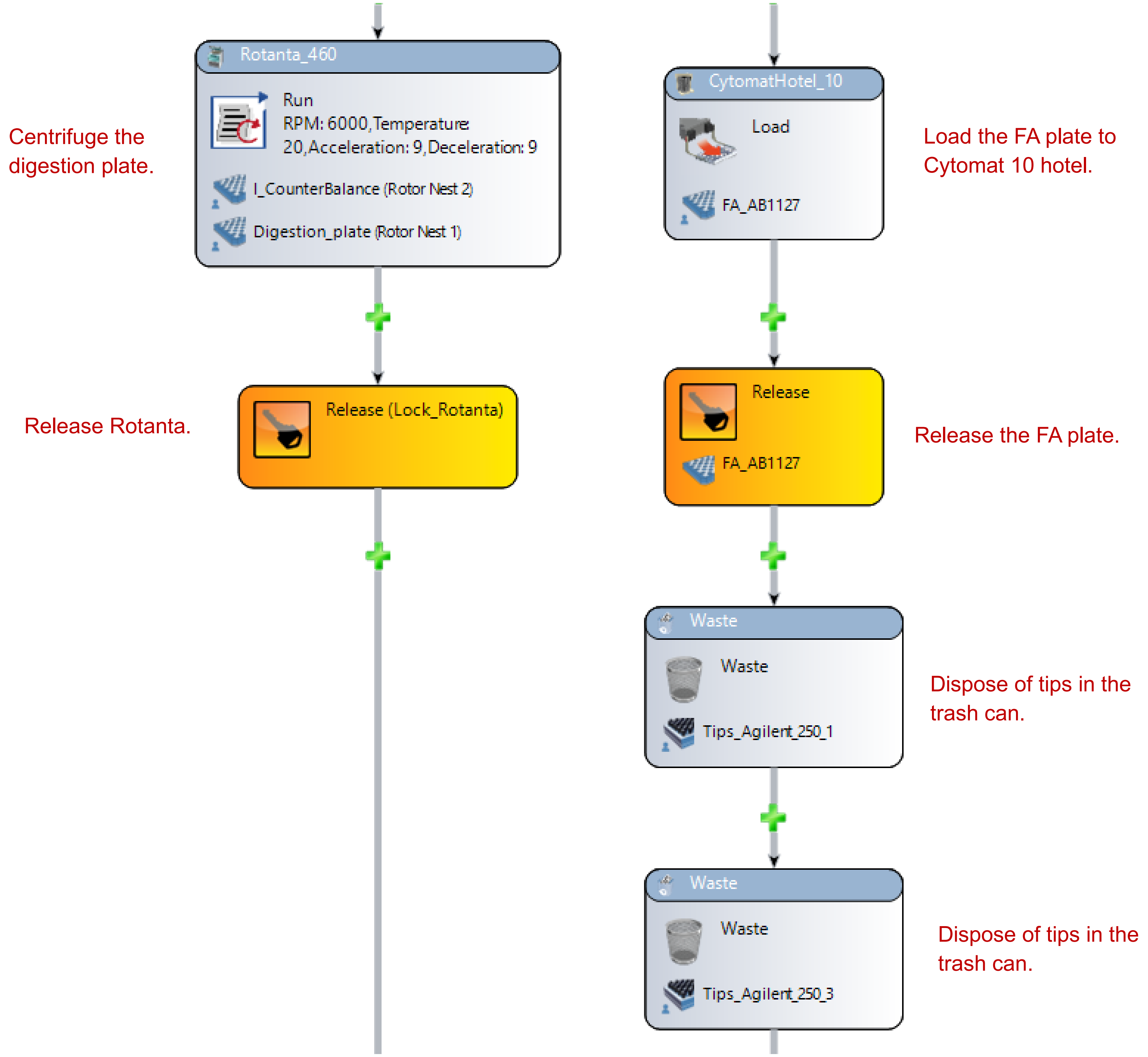
Centrifuge the digestion plate.
Rotanta_460
Run
RPM: 6000, Temperature: 20, Acceleration: 9, Deceleration: 9
I_CounterBalance (Rotor Nest 2)
Digestion_plate (Rotor Nest 1)
Release Rotanta.
Release (Lock_Rotanta)
CytomatHotel_10
Load
FA_AB1127
Load the FA plate to Cytomat 10 hotel.
Release
FA_AB1127
Release the FA plate.
Waste
Waste
Tips_Agilent_250_1
Dispose of tips in the trash can.
Waste
Waste
Tips_Agilent_250_3
Dispose of tips in the trash can.

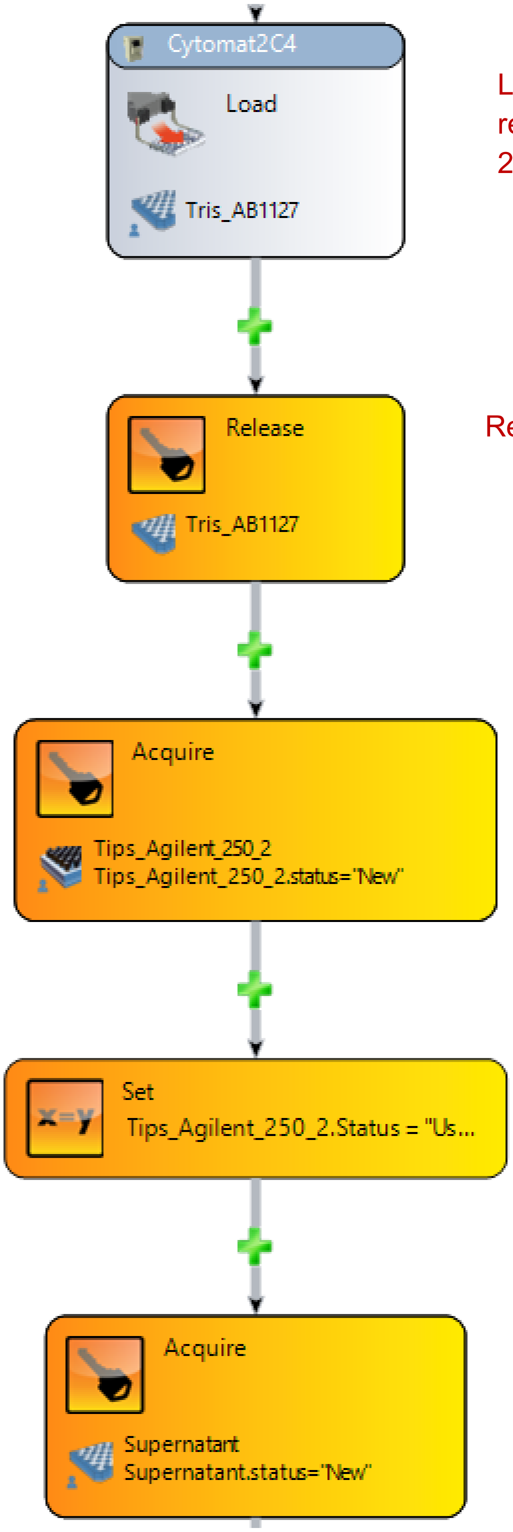

Load tris-HCl reservoir to Cytomat 2C4.

Release tris-HCl.

Acquire tips.

Set the status of tips.

Acquire a plate for supernatant.

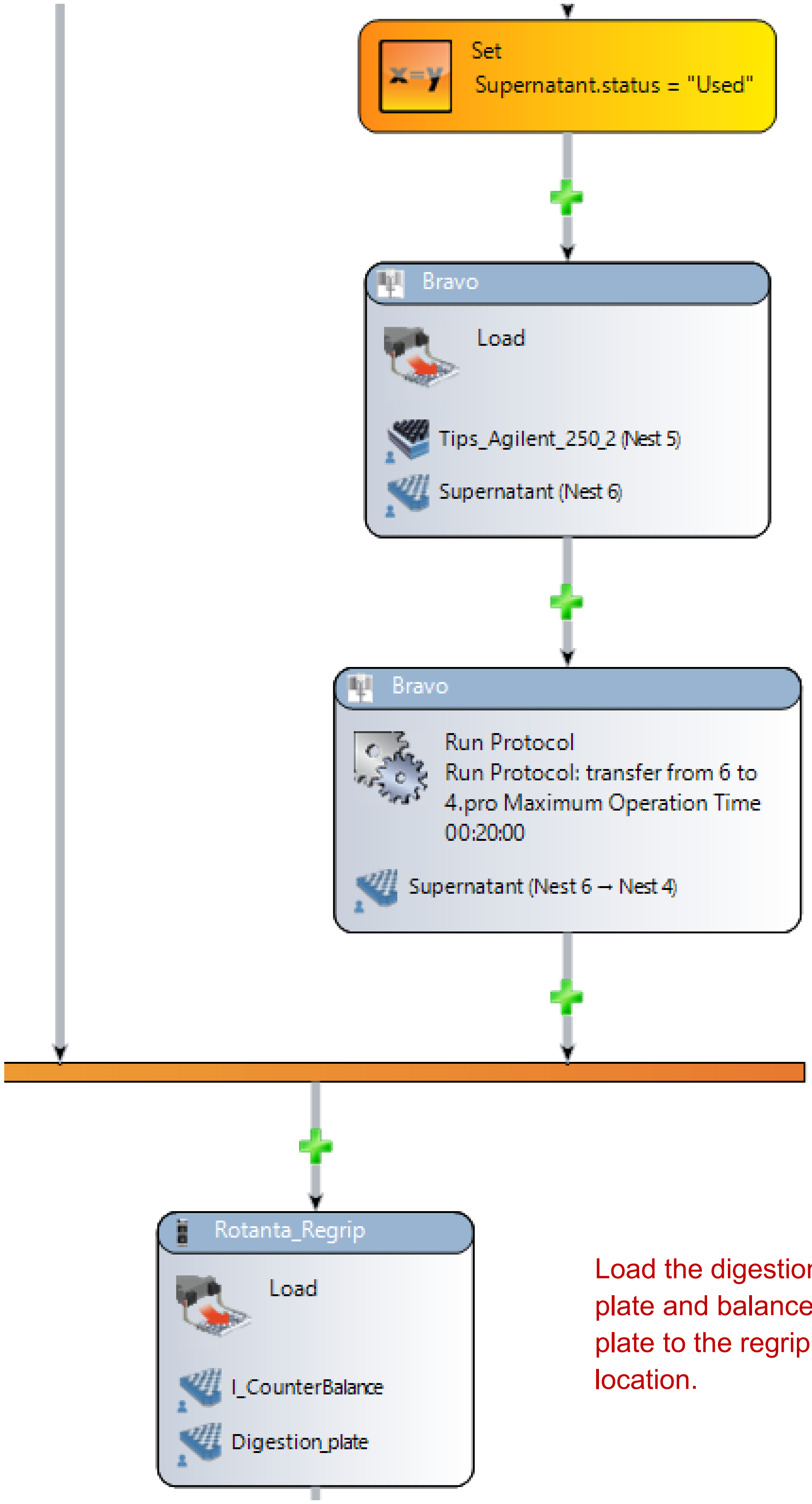

Set the status of the supernatant plate.

Load tips and the supernatant plate to Bravo.

Run the Bravo protocol to transfer the supernatant plate from 6 to 4.

Load the digestion plate and balance plate to the regrip location.

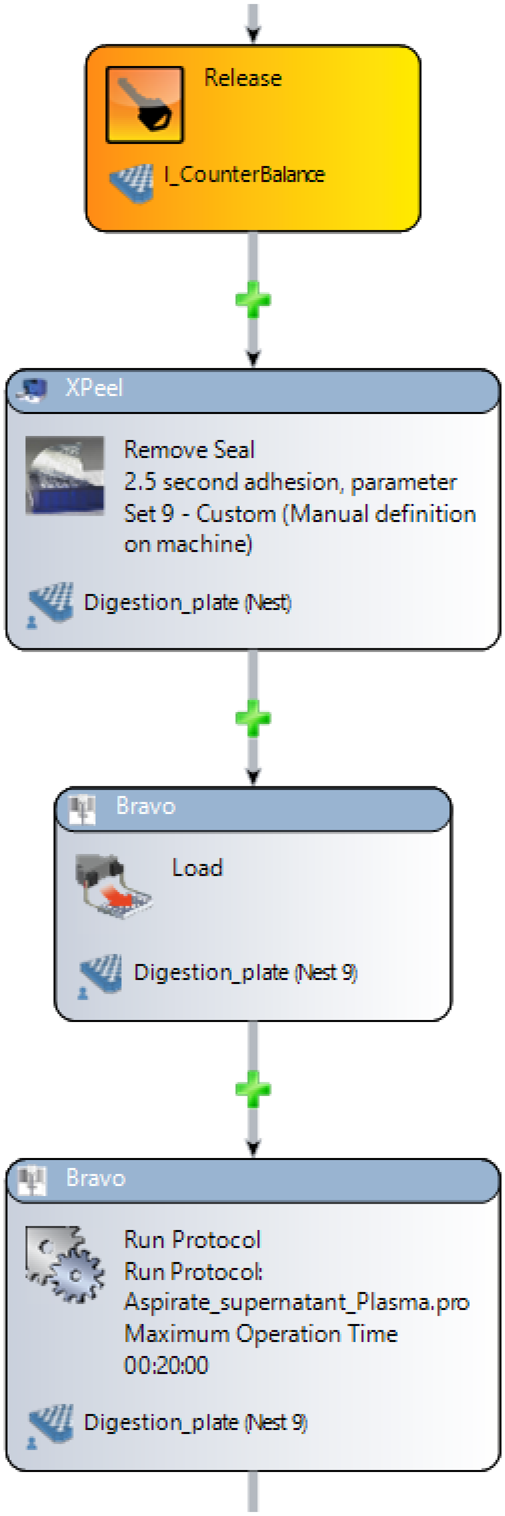

Release the balance plate.

Peel off the film of the digestion plate.

Load the digestion plate to Bravo.

Run the Bravo protocol to transfer the supernatant from the digestion plate to the supernatant plate.

Load the digestion plate to Cytomat 2C4 hotel.

Dispose of tips in the trash can.

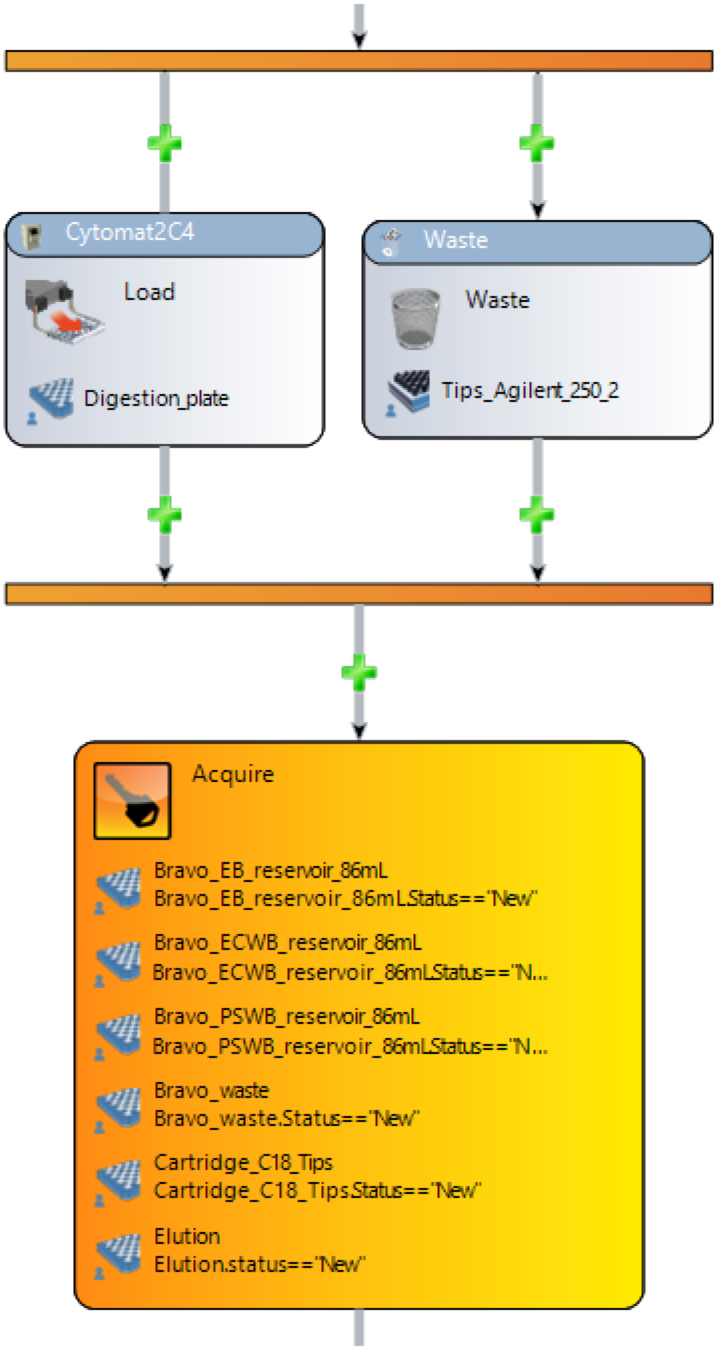

Acquire reagents and labware (e.g., tips and cartridges) for desalting.

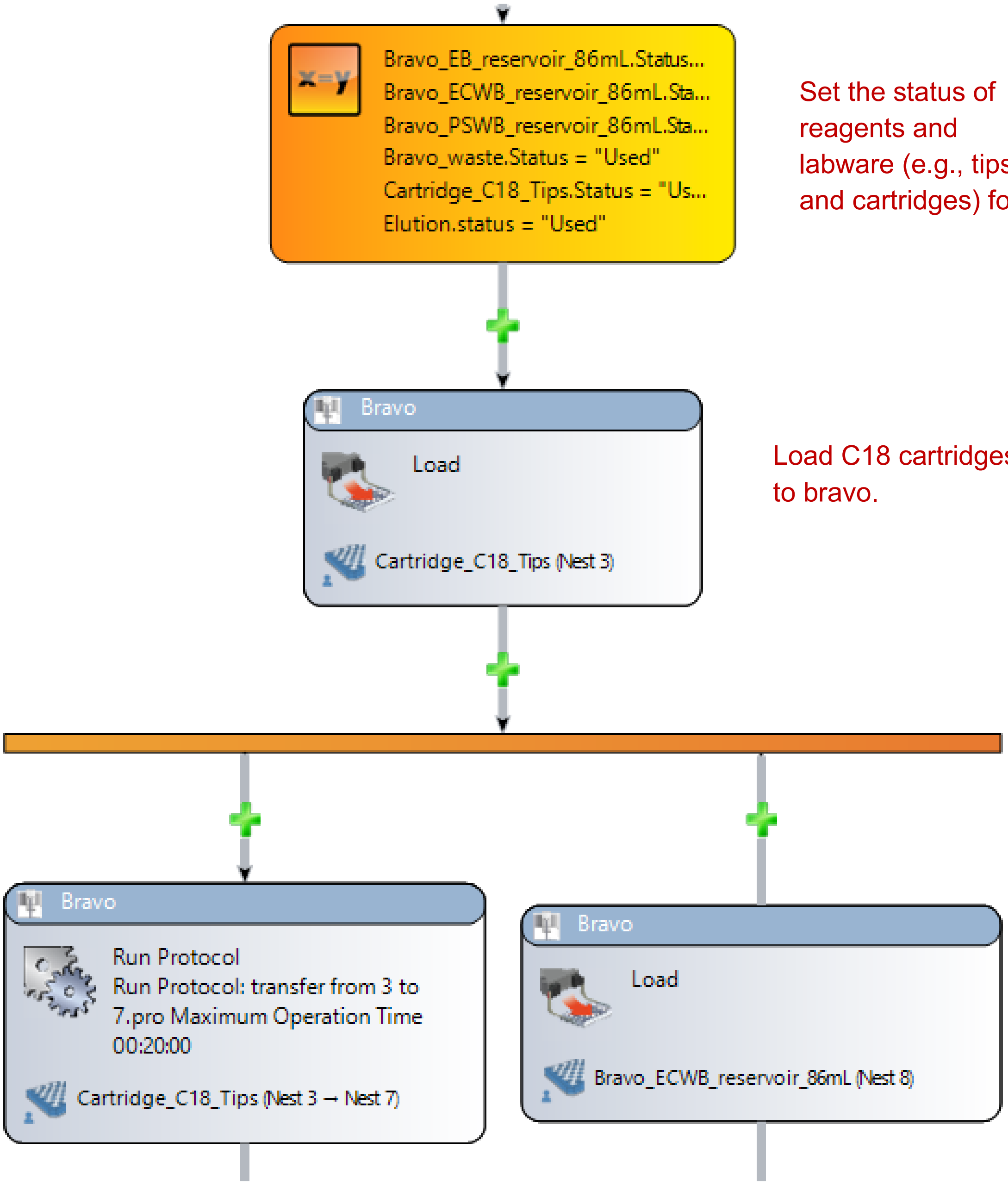

Set the status of reagents and labware (e.g., tips and cartridges) for

Load C18 cartridges to bravo.

Run Bravo protocol to transfer C18 cartridges from nest 3 to nest 7 on Bravo bench.

Load the cartridge washing buffer reservoir to Bravo.

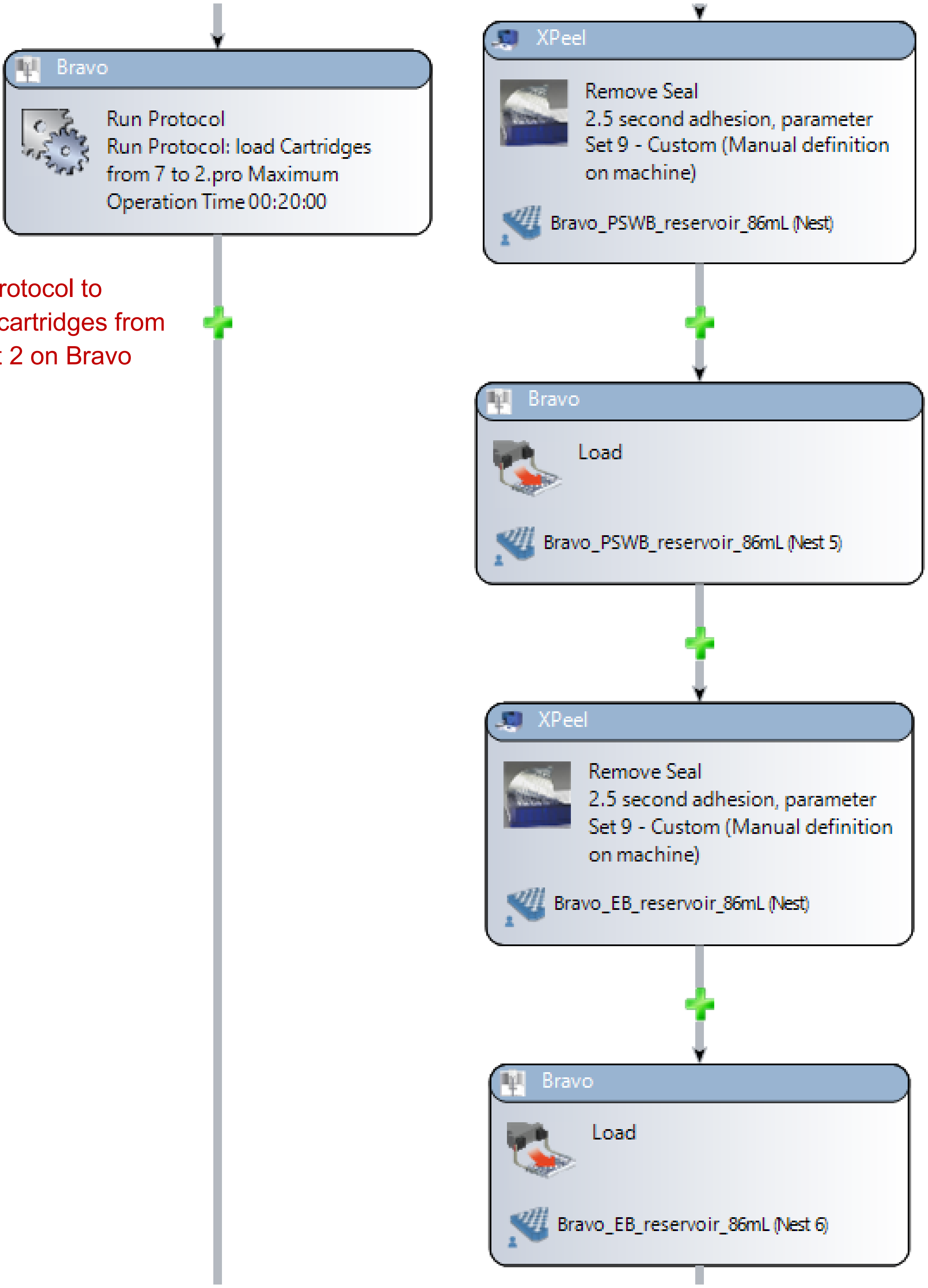

Run Bravo protocol to transfer C18 cartridges from nest 7 to nest 2 on Bravo bench.

Peel off the film of the washing buffer reservoir.

Load the washing buffer reservoir to Bravo.

Load the washing buffer reservoir to Bravo.

Load the eluting buffer reservoir to Bravo.

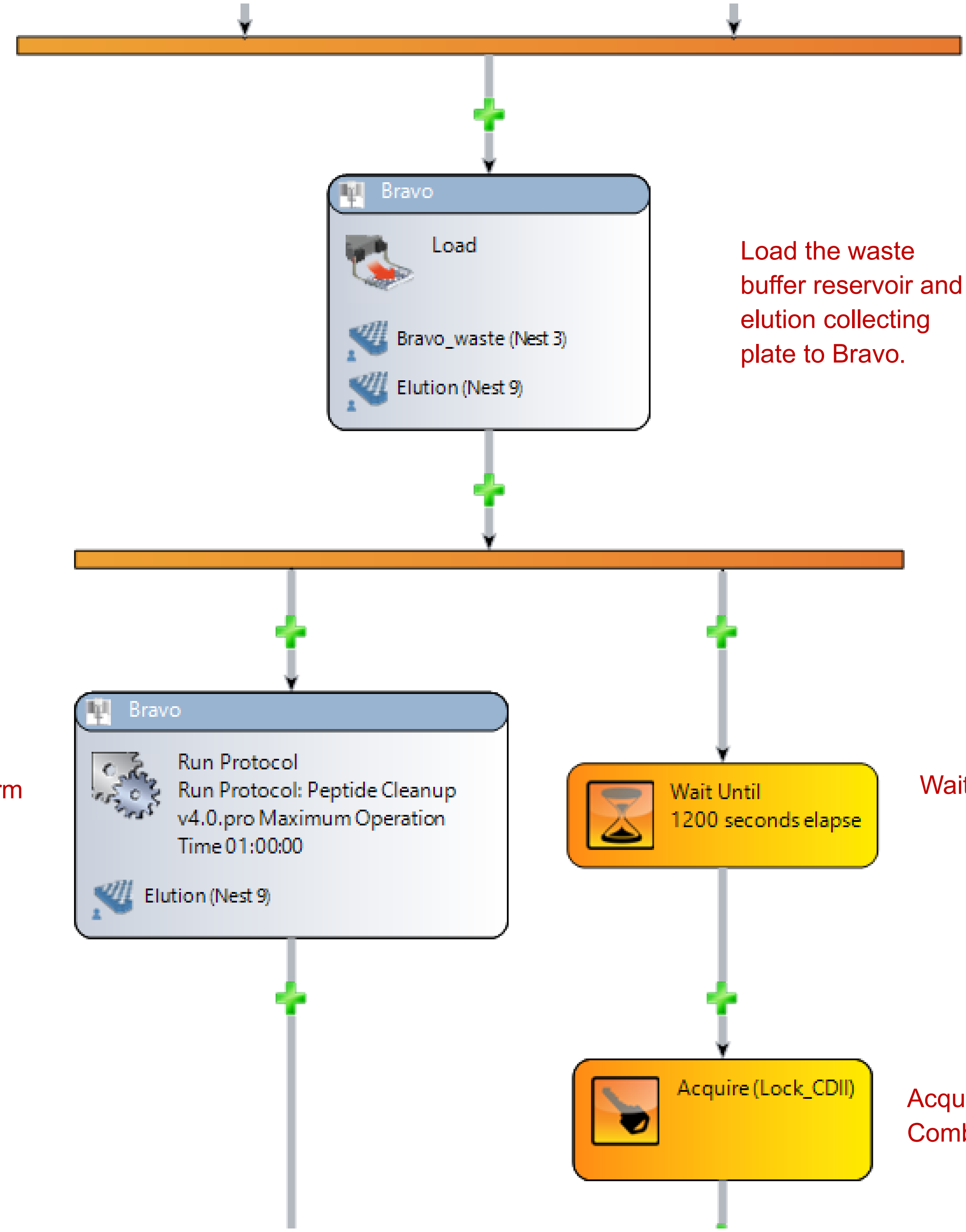

Load the waste buffer reservoir and elution collecting plate to Bravo.

Run the Bravo protocol to perform peptide cleanup.

Wait for 20min.

Acquire CombiDancer.

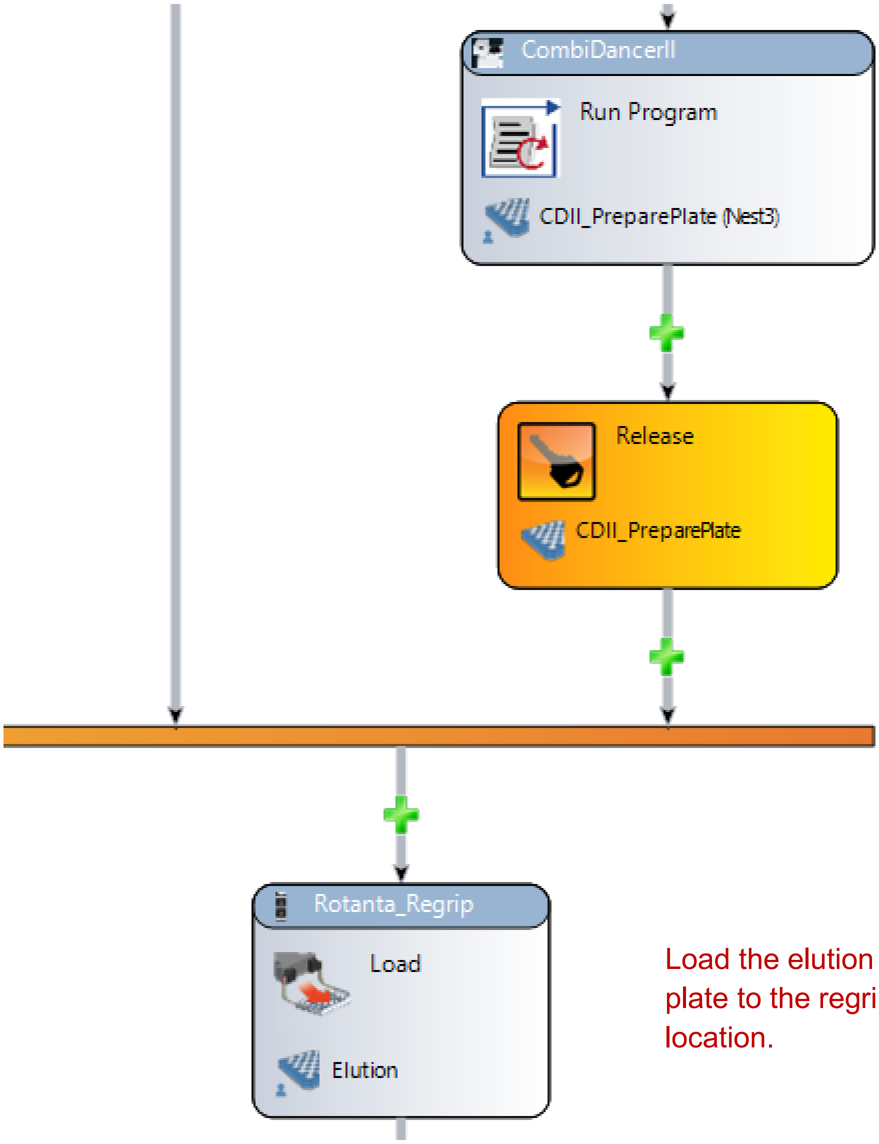

Pre-heat CombiDancer.

Release CombiDancer.

Load the elution plate to the regrip location.

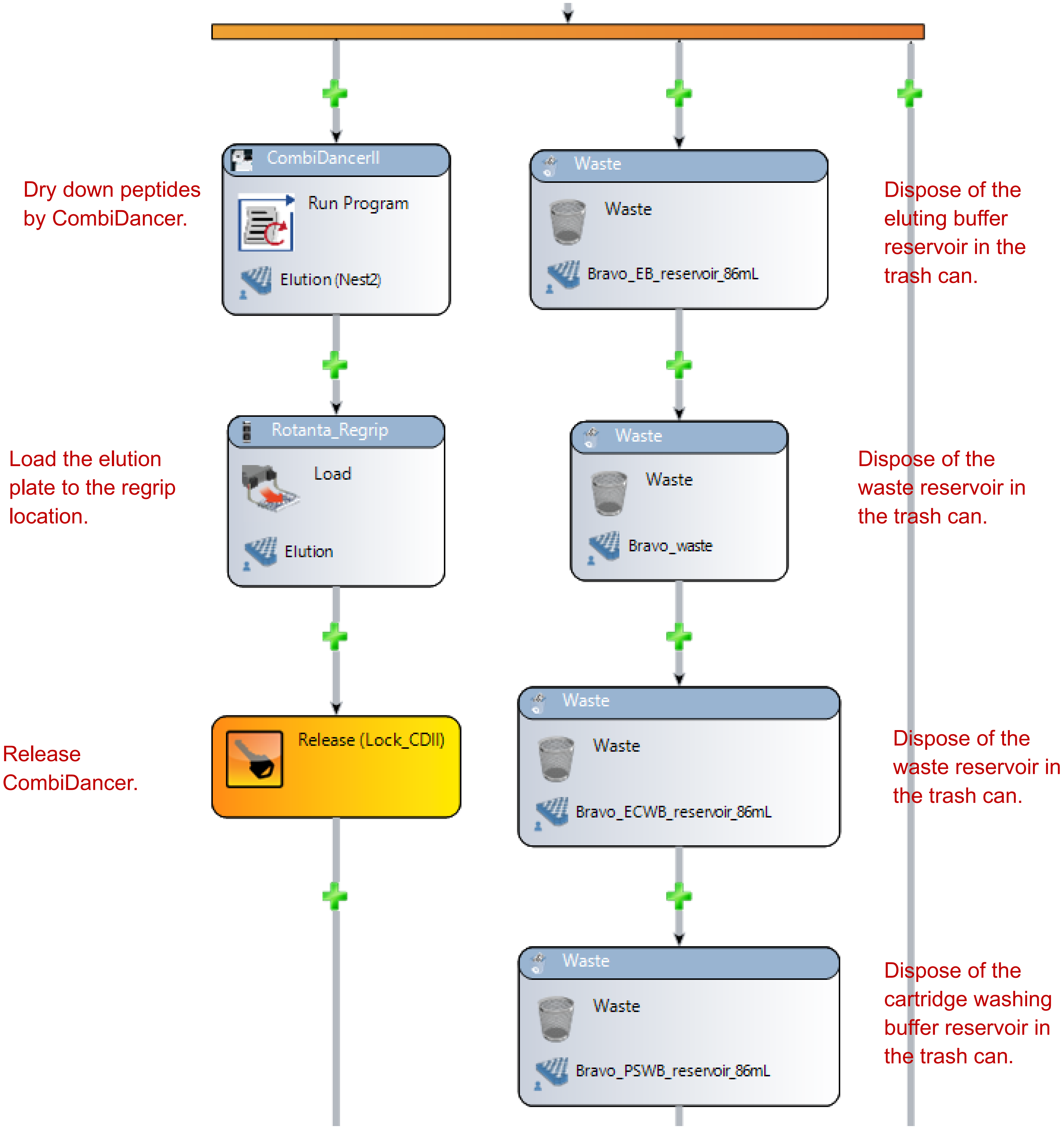
Dry down peptides by CombiDancer.
CombiDancerII
Run Program
Elution (Nest2)
Waste
Waste
Bravo_EB_reservoir_86mL
Dispose of the eluting buffer reservoir in the trash can.
Load the elution plate to the regrip location.
Rotanta_Regrip
Load
Elution
Waste
Waste
Bravo_waste
Dispose of the waste reservoir in the trash can.
Release CombiDancer.
Release (Lock_CDII)
Waste
Waste
Bravo_ECWB_reservoir_86mL
Dispose of the waste reservoir in the trash can.
Waste
Waste
Bravo_PSWB_reservoir_86mL
Dispose of the cartridge washing buffer reservoir in the trash can.

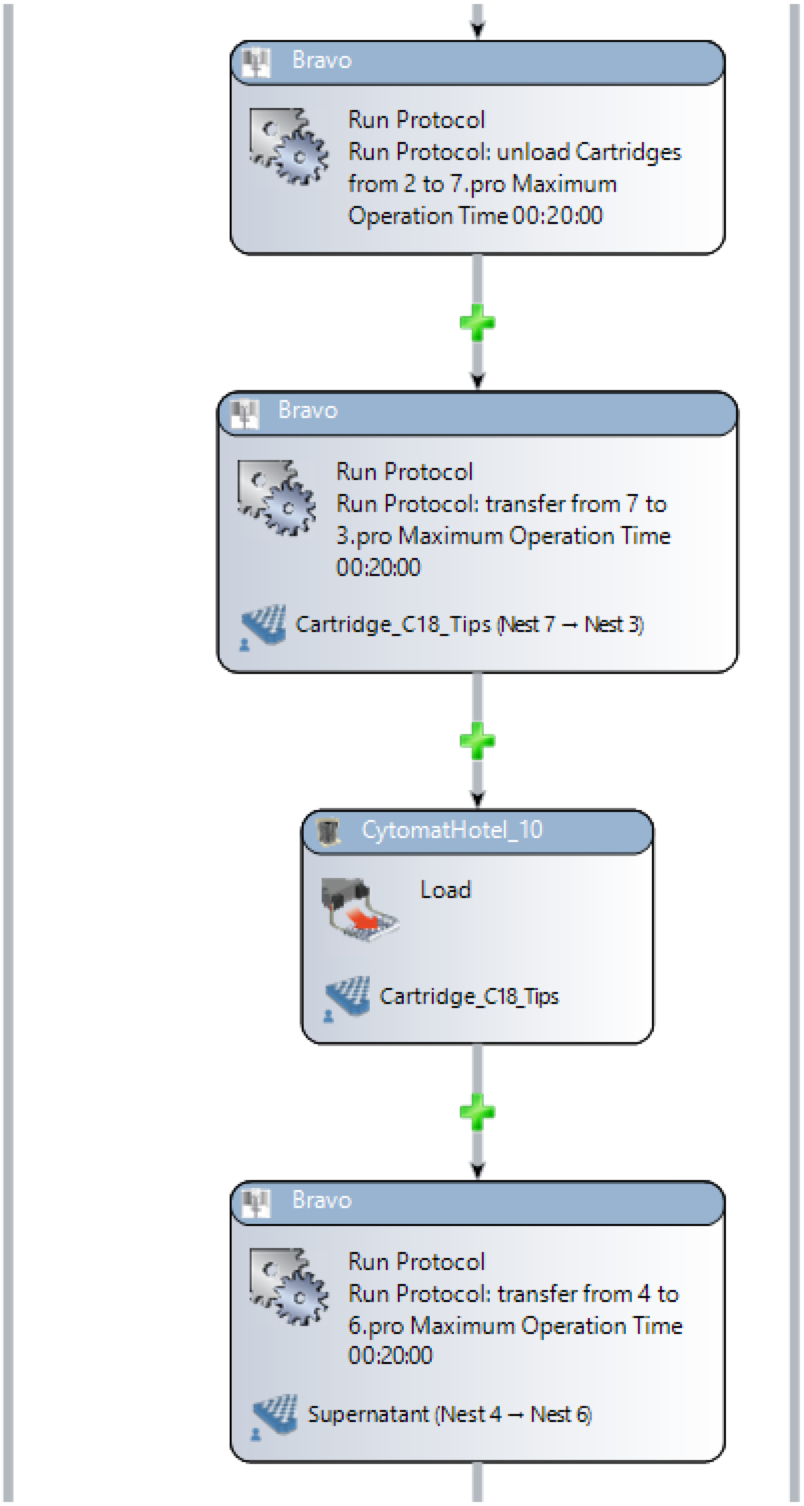

Run the Bravo protocol to unload the cartridges to nest 7.

Run the Bravo protocol to transfer the cartridges from nest 7 to nest 3.

Load the C18 cartridges to Cytomat 10 hotel.

Run the Bravo protocol to transfer the flow-through plate from nest 4 to nest 6.

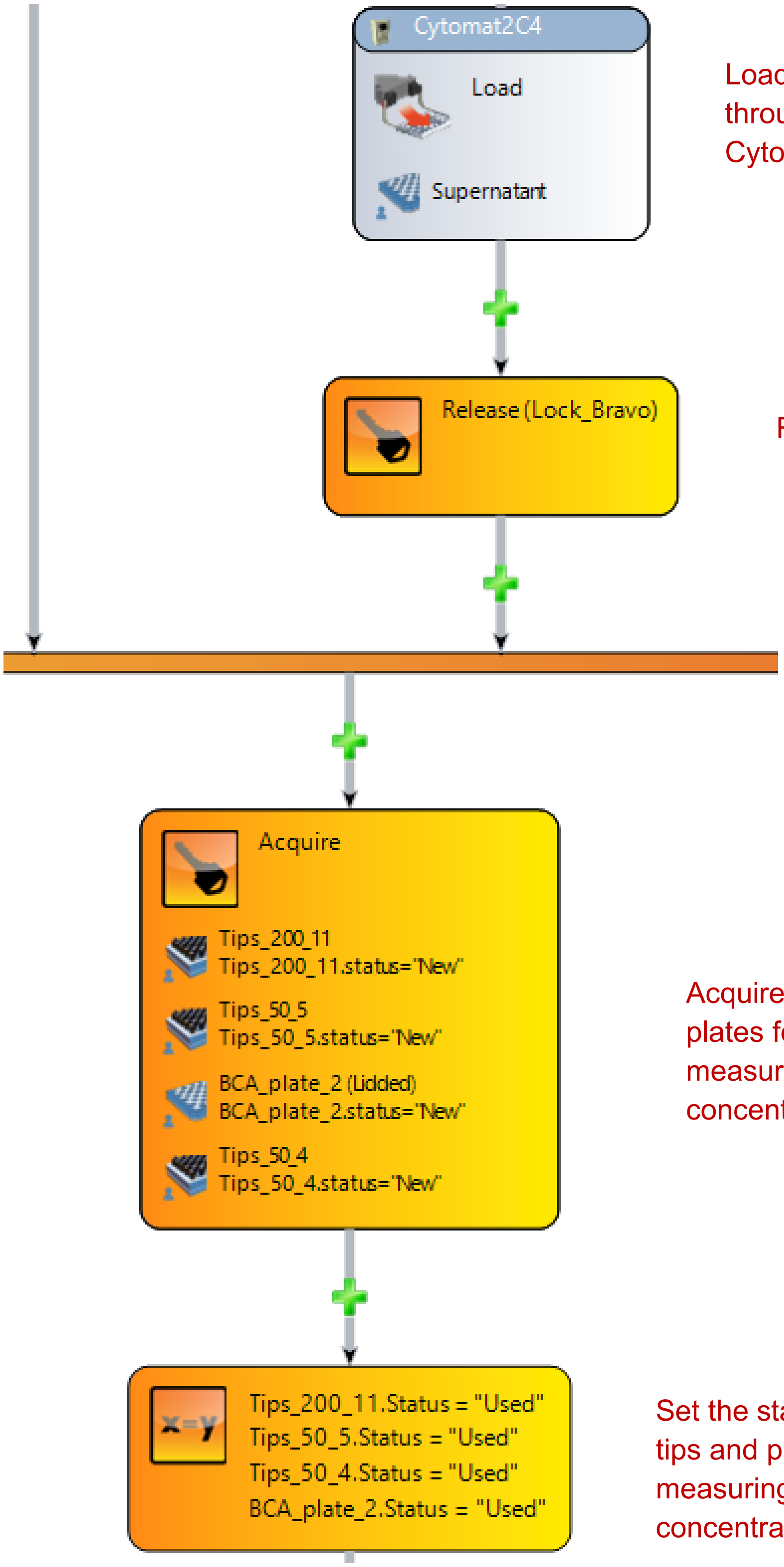

Load the flow-through plate to Cytomat 2 hotel.

Release Bravo.

Acquire tips and plates for measuring peptide concentration.

Set the status of tips and plates for measuring peptide concentration.

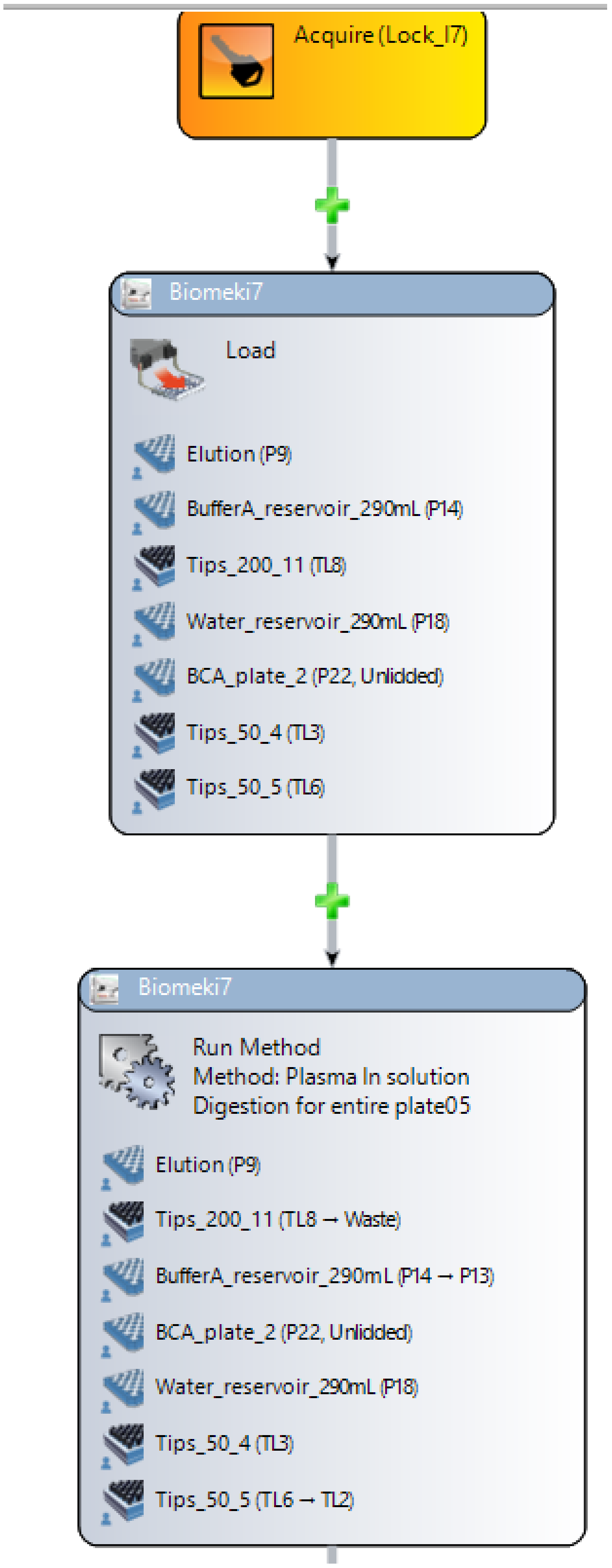

Acquire Biomek i7.

Load tips and buffer to Biomek i7.

Dissolve the peptides by Biomek i7.

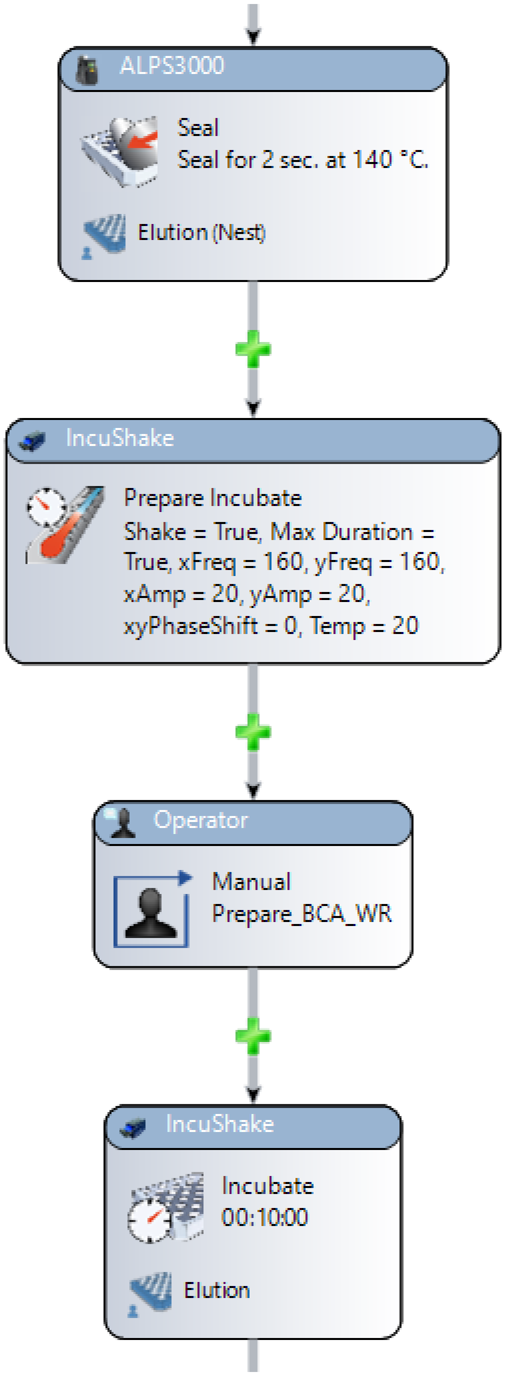

Seal the elution plate by ALPS3000.

Prepare Inheco.

Reminder the operator to prepare BCA assay reagents.

Incubate the elution plate in Inheco to dissolve peptides.

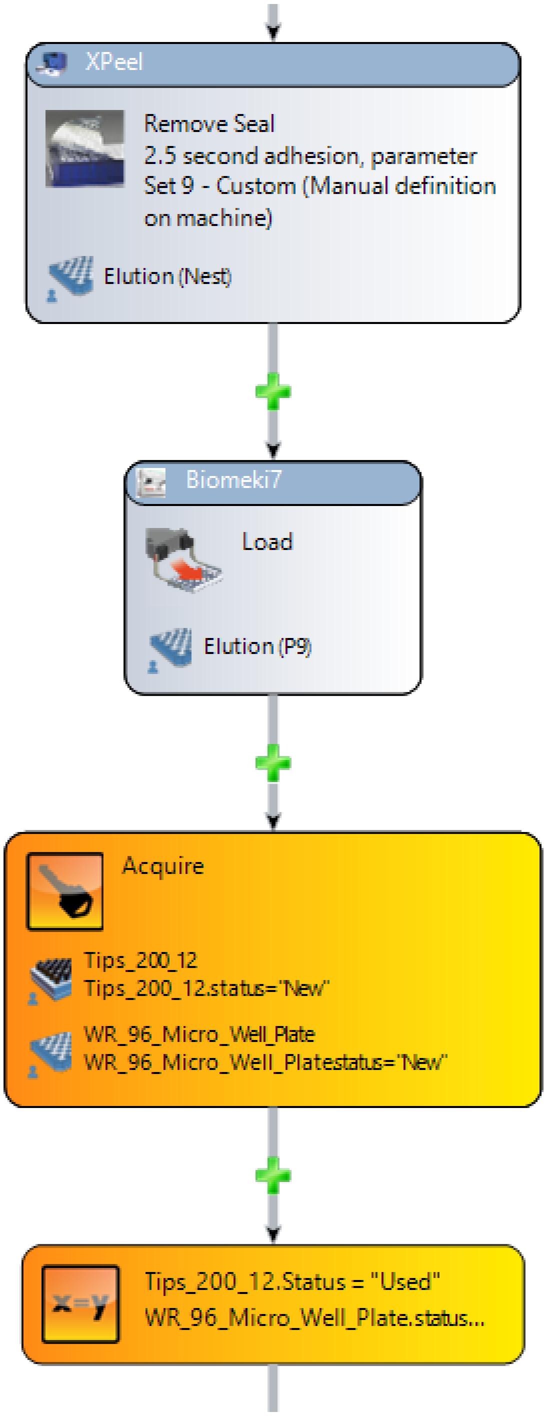

Peel off the film of the elution plate.

Load the elution plate to Biomek i7.

Acquire tips and plates.

Set the status of tips and plate.

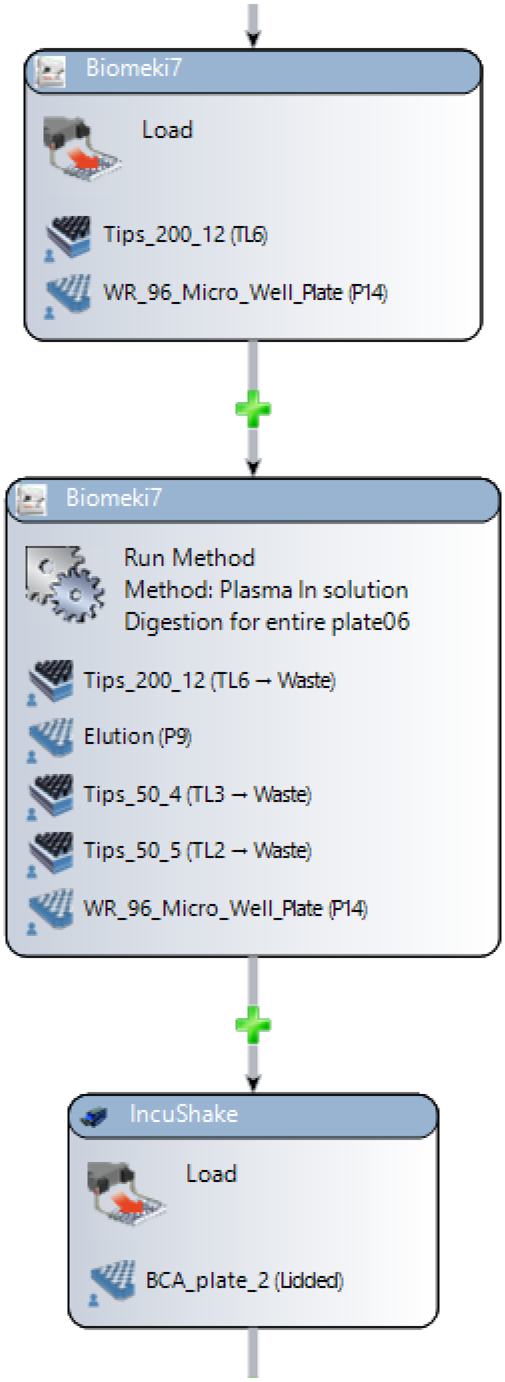

Load tips and plate to Biomek i7.

Make the mixture of peptides and BCA reagent by Biomek i7.

Load the BCA plate with its lid to Inheco.

Incubate the BCA plate at 37 ℃ for 15min.

Load the water reservoir to Cytomat 10 hotel.

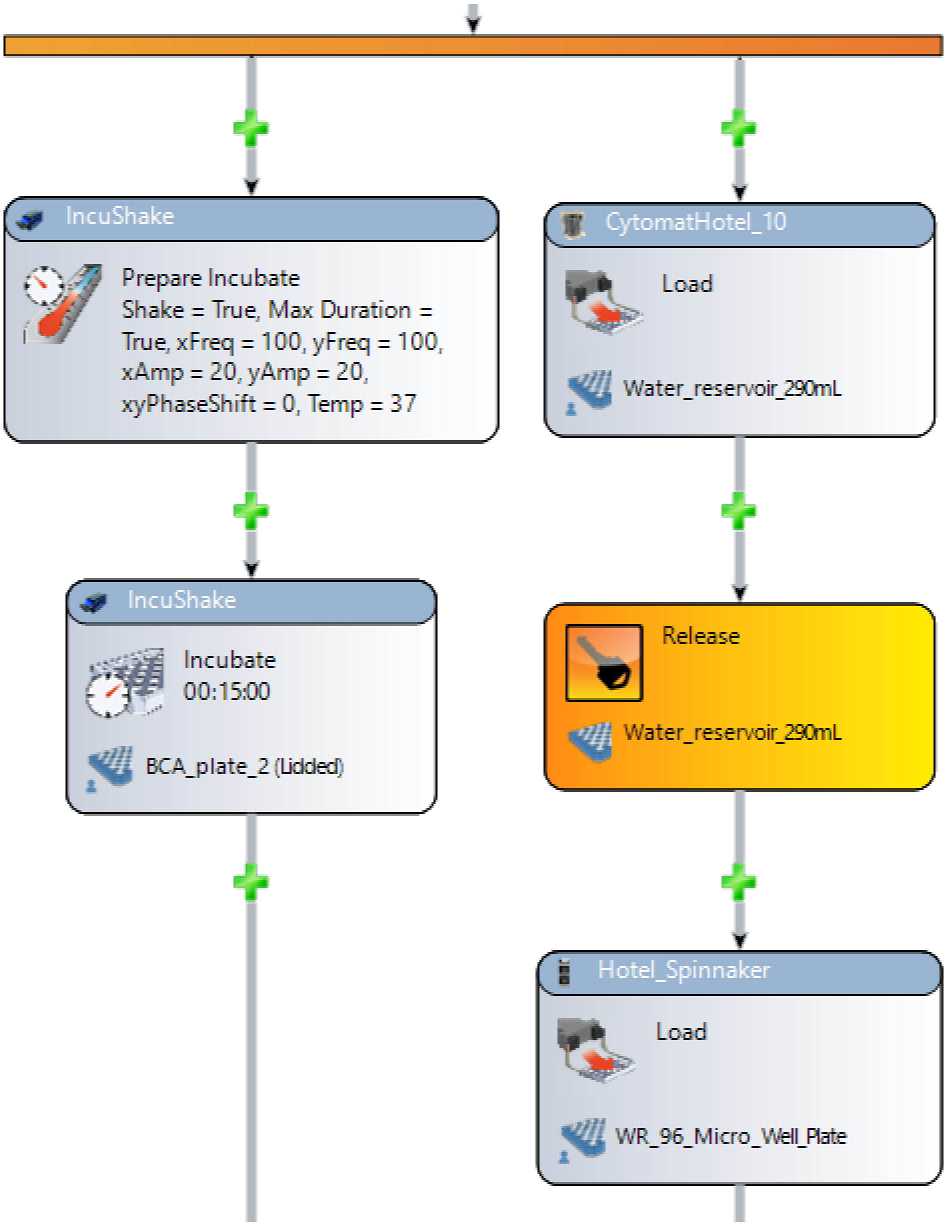

Incubate the BCA plate at 37 ℃ for 15min.

Release the water reservoir.

Load the BCA reagent reservoir to Cytomat 10 hotel.

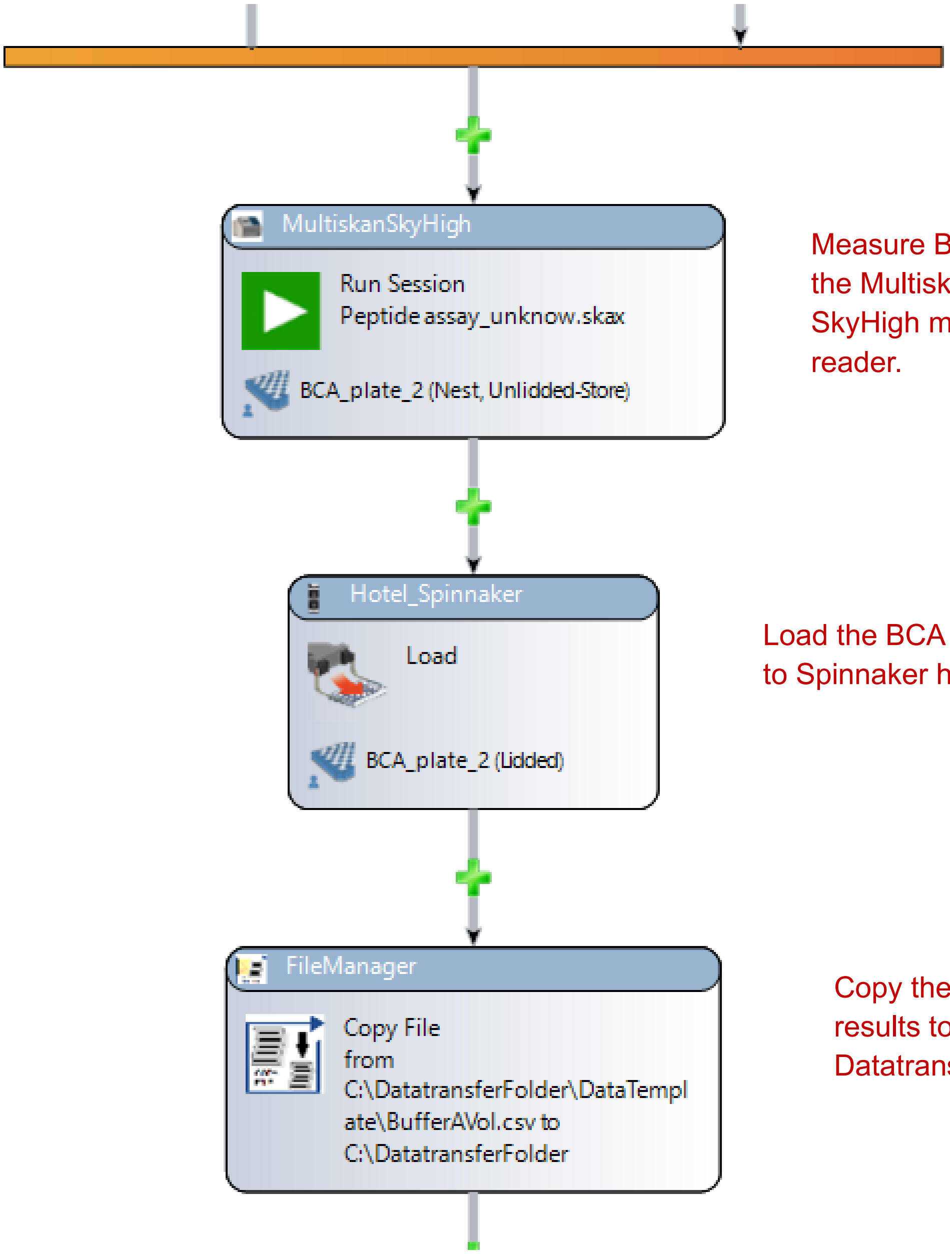

Measure BCA with the Multiskan SkyHigh microplate reader.

Load the BCA plate to Spinnaker hotel.

Copy the BCA results to Datatransfer folder.

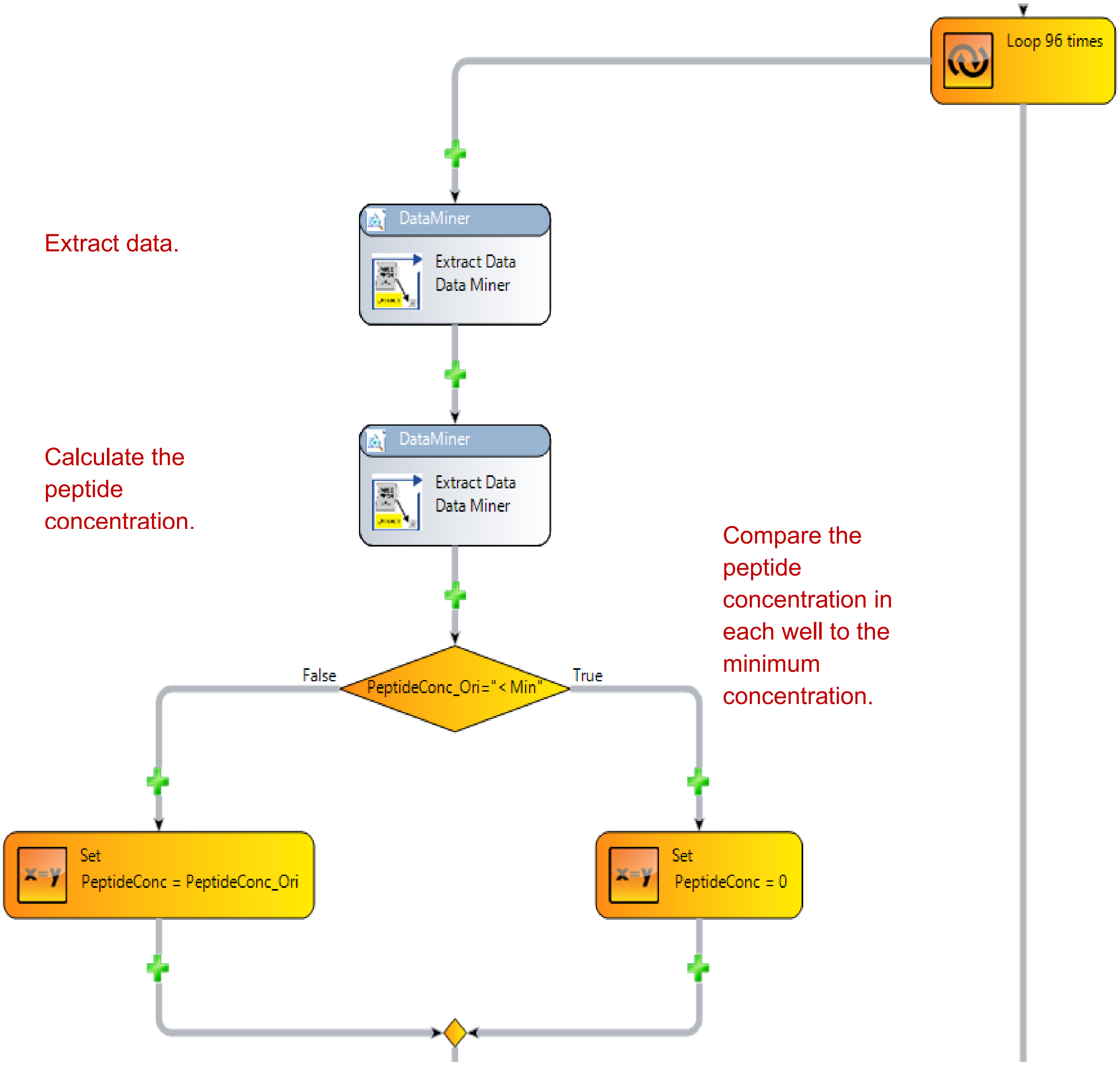

If the peptide concentration exceeds the minimum value, retain the original value; otherwise, set it to zero.

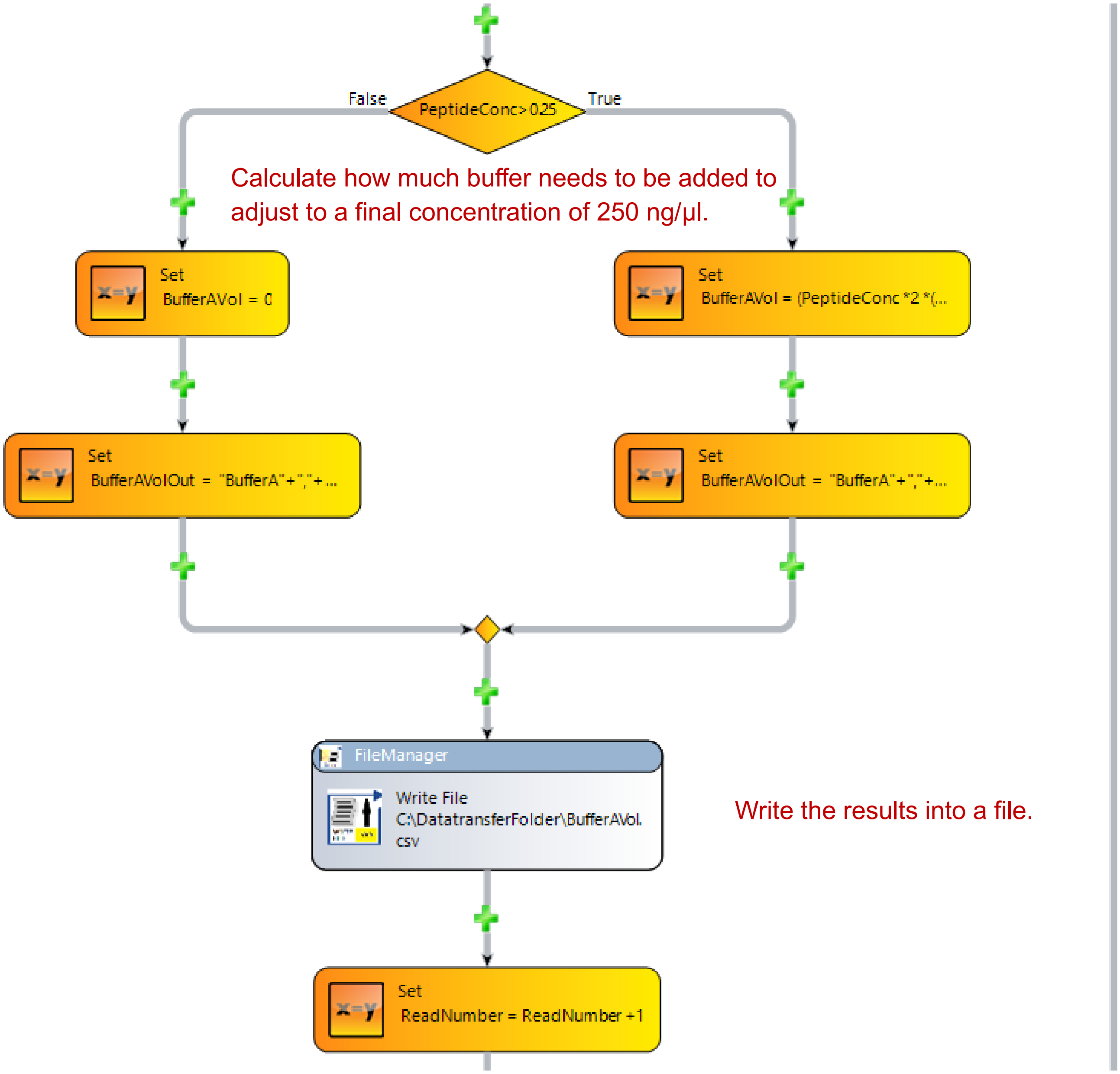

Calculate how much buffer needs to be added to adjust to a final concentration of 250 ng/µl.

Write the results into a file.

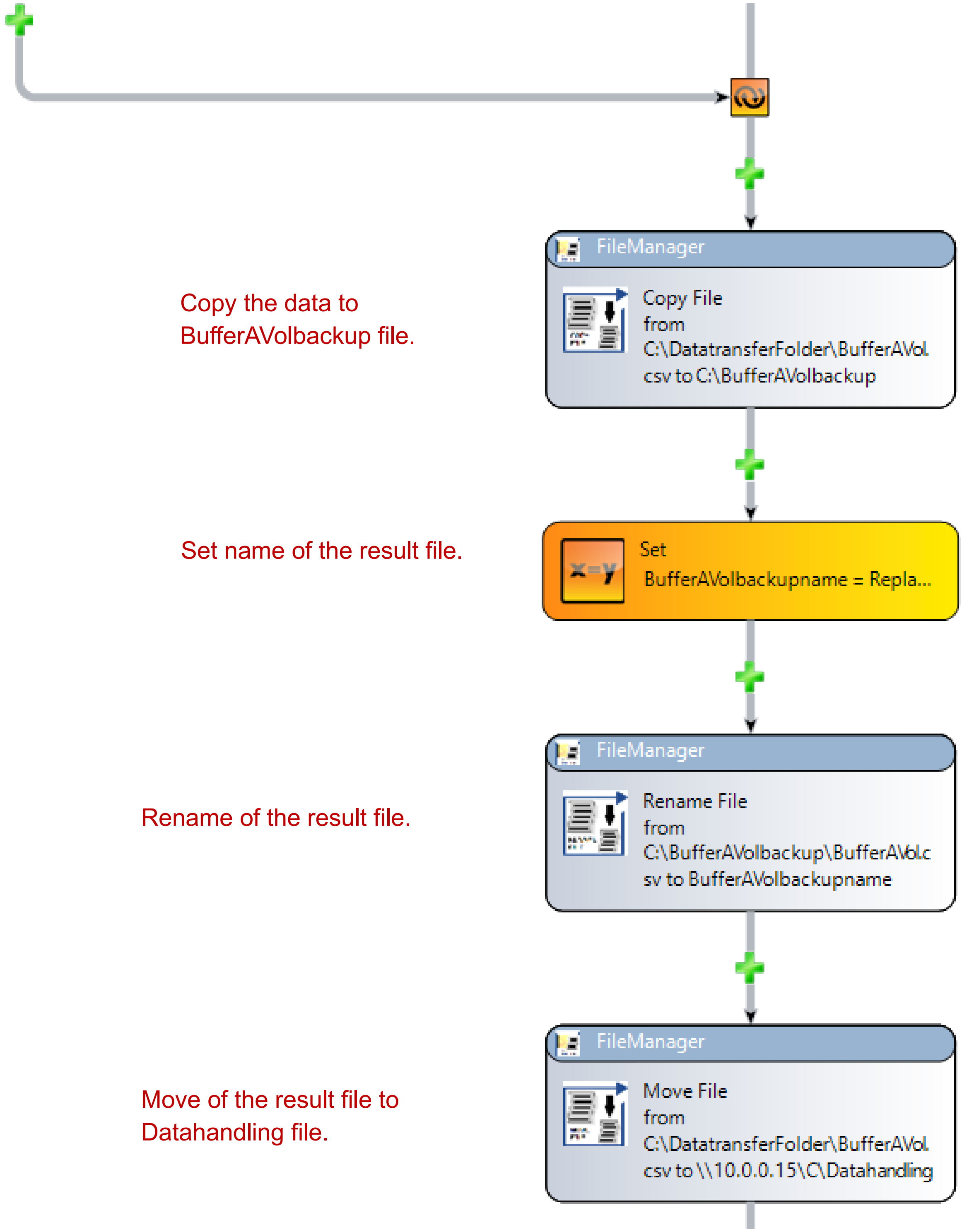
FileManager
Copy File
from
C:\DatatransferFolder\BufferAVol.
csv to C:\BufferAVolbackup
Copy the data to
BufferAVolbackup file.
Set
BufferAVolbackupname = Repla...
Set name of the result file.
FileManager
Rename File
from
C:\BufferAVolbackup\BufferAVol.c
sv to BufferAVolbackupname
Rename of the result file.
FileManager
Move File
from
C:\DatatransferFolder\BufferAVol.
csv to \\10.0.0.15\C\Datahandling
Move of the result file to
Datahandling file.

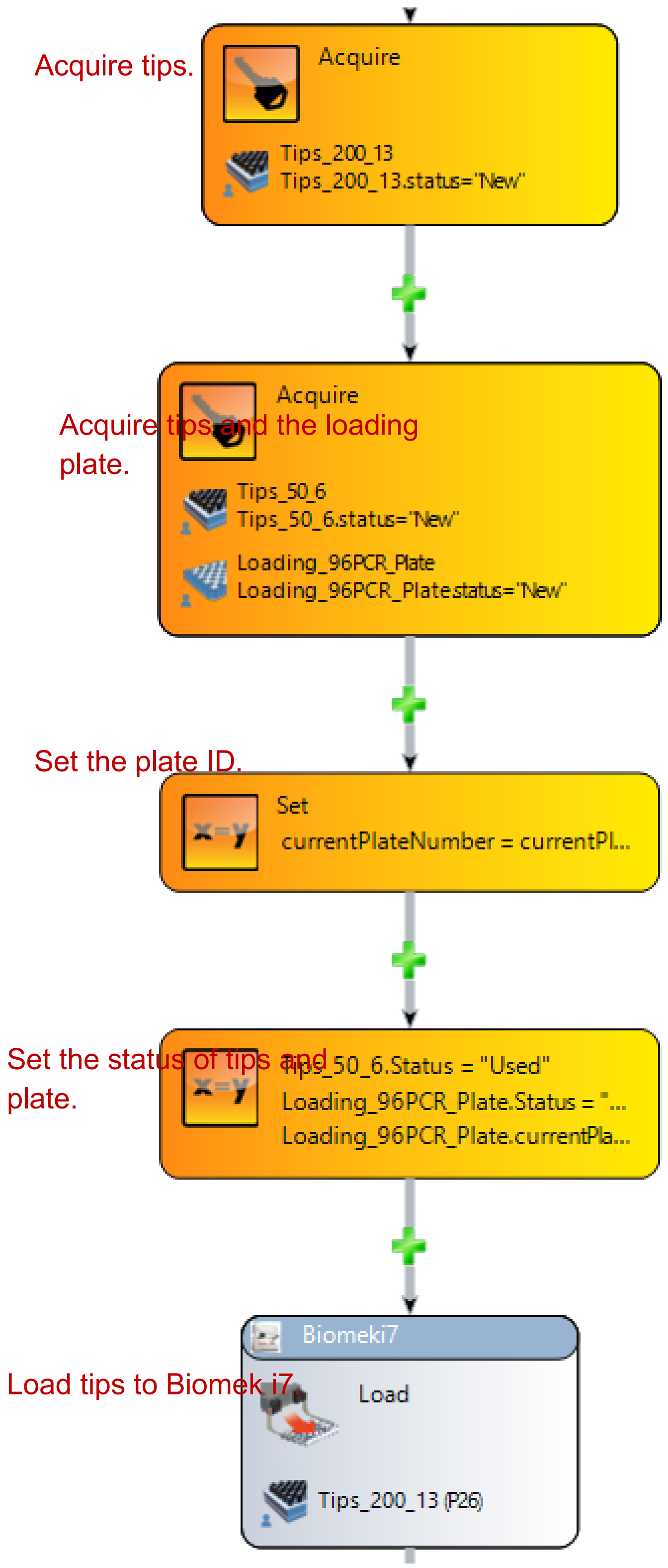
Acquire tips.
Acquire
Tips_200_13
Tips_200_13.status='New'
Acquire tips and the loading plate.
Acquire
Tips_50_6
Tips_50_6.status='New'
Loading_96PCR_Plate
Loading_96PCR_Plate.status='New'
Set the plate ID.
Set
currentPlateNumber = currentPl...
Set the status of tips and plate.
Tips_50_6.Status = "Used"
Loading_96PCR_Plate.Status = "...
Loading_96PCR_Plate.currentPla...
Biomeki7
Load tips to Biomek i7.
Load
Tips_200_13 (P26)

图 36

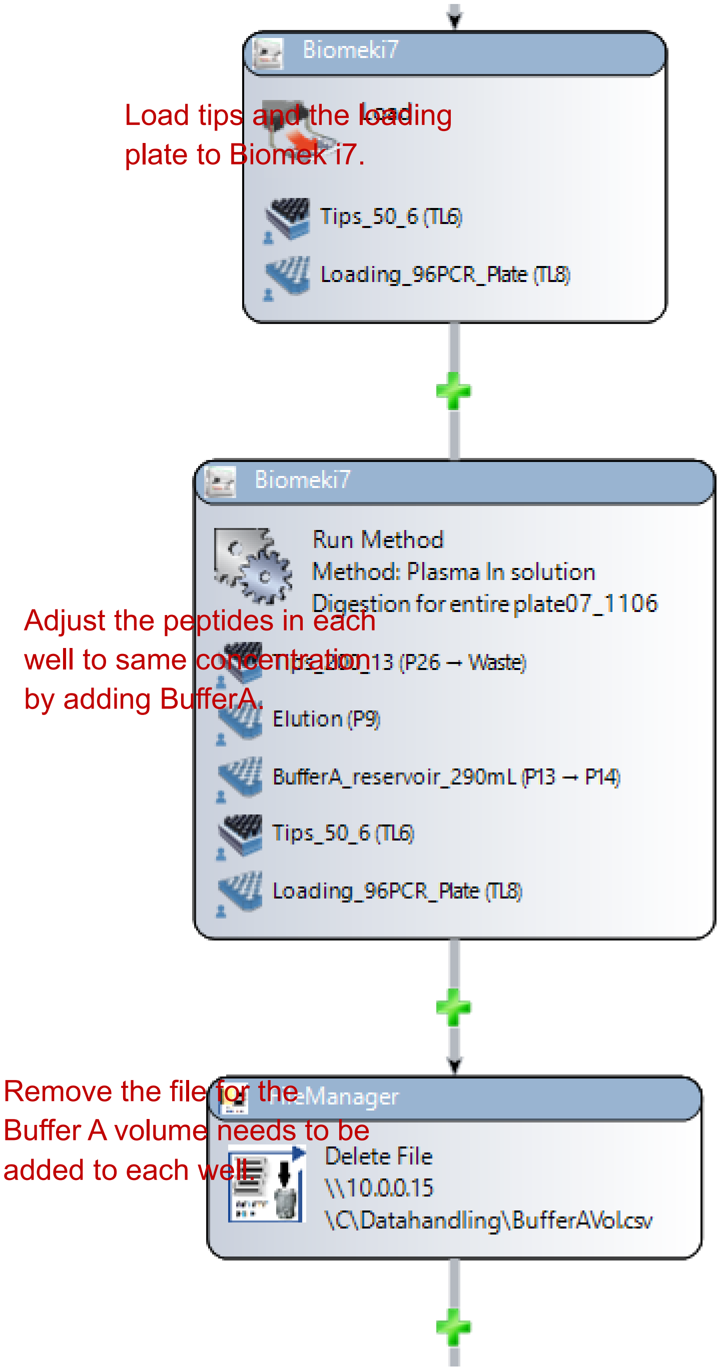
Biomeki7
Load tips and the loading plate to Biomek i7.
Load
Tips_50_6 (TL6)
Loading_96PCR_Plate (TL8)
Biomeki7
Run Method
Method: Plasma In solution
Digestion for entire plate07_1106
Adjust the peptides in each well to same concentration by adding BufferA.
Elution (P9)
BufferA_reservoir_290mL (P13 → P14)
Tips_50_6 (TL6)
Loading_96PCR_Plate (TL8)
Remove the file for the Buffer A volume needs to be added to each well.
Delete File
\\10.0.0.15
\C\Datahandling\BufferAVol.csv

Seal the elution plate by ALPS3000.

Load the elution plate to Inheco.

Prepare Inheco.

Incubate the elution plate in Inheco for 5min.

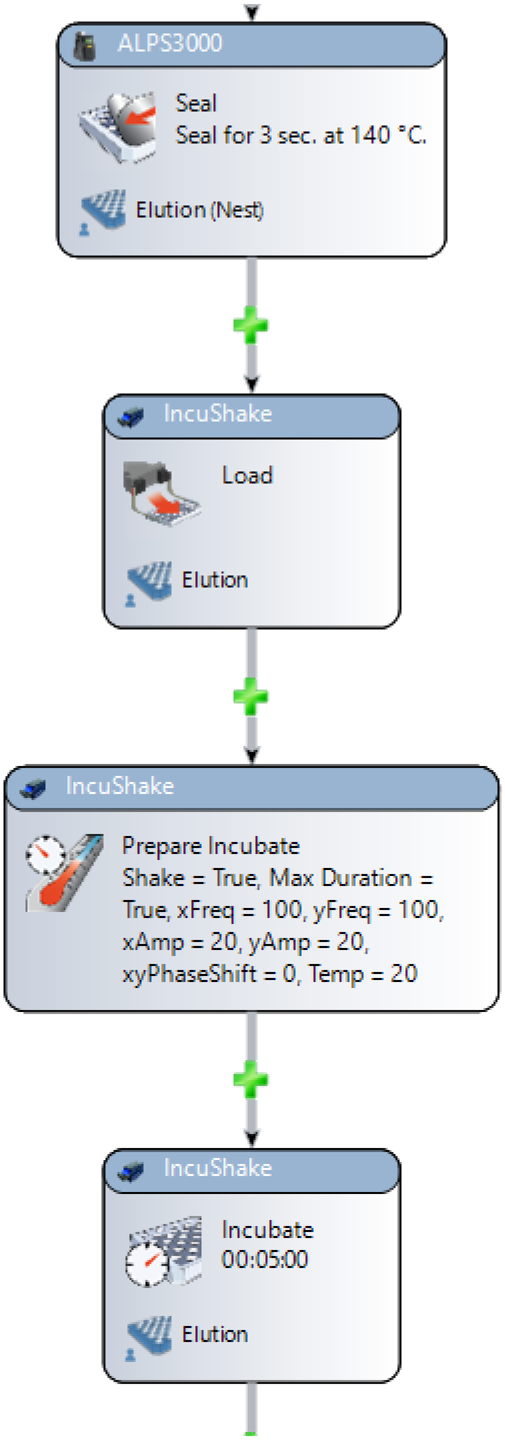

Load the elution plate to the regrip location.

Load the elution plate and the balance plate to Rotanta.

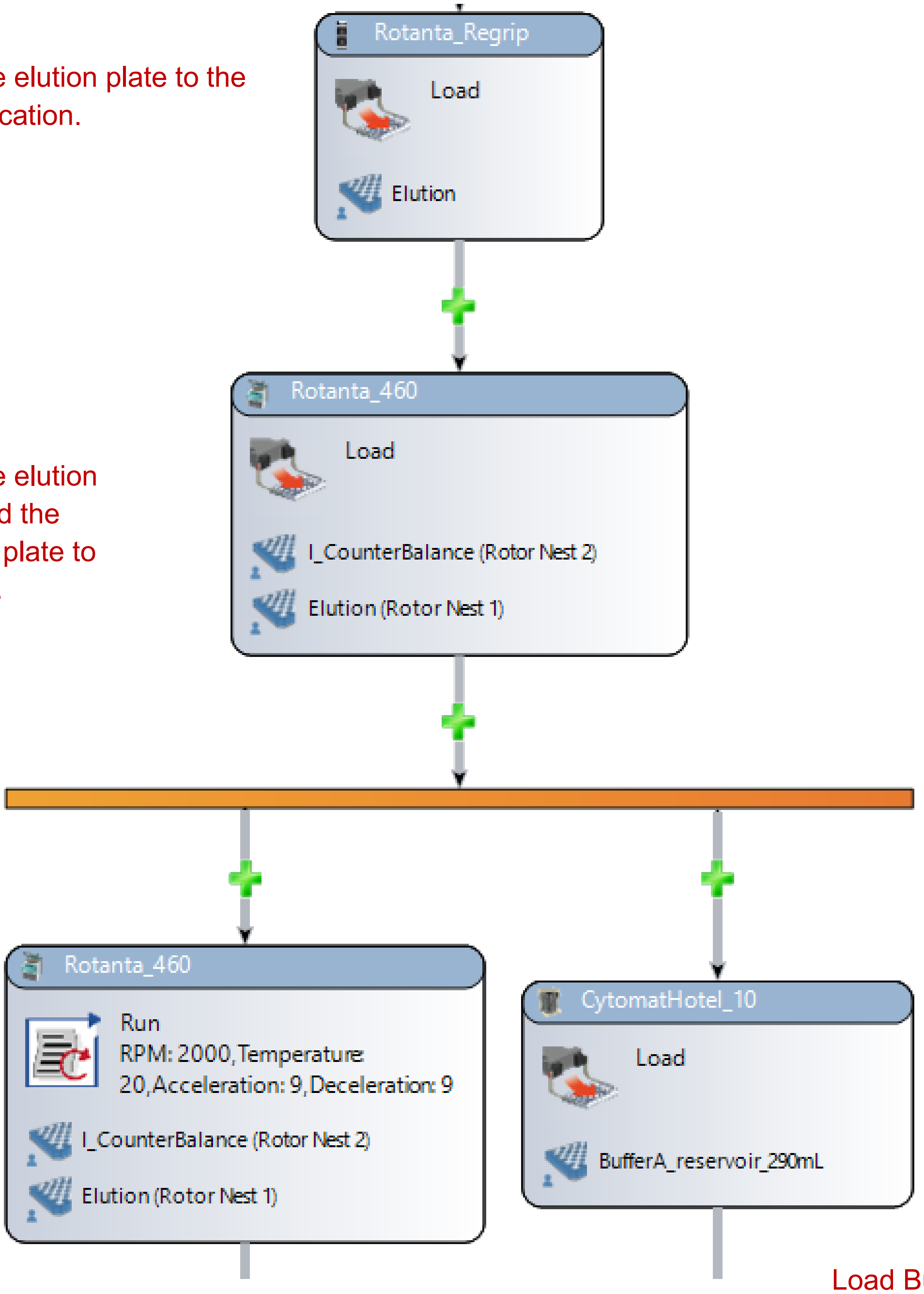

Load Buffer A to Cytomat 10 hotel.

Centrifuge the elution plate and the balance plate.

Load the elution plate and the balance plate to the regrip location.

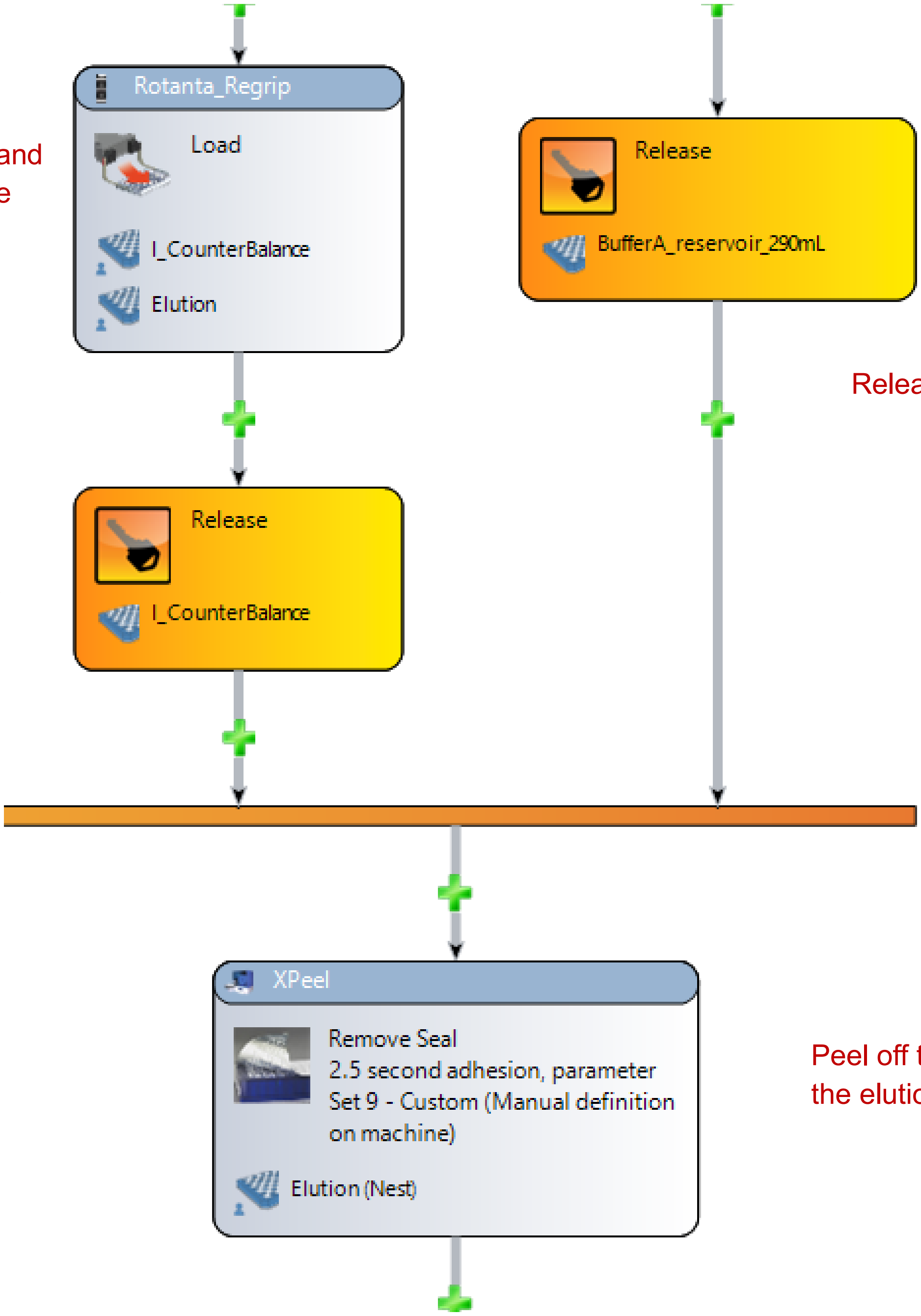

Release Buffer A.

Release the balance plate.

Peel off the film of the elution plate.

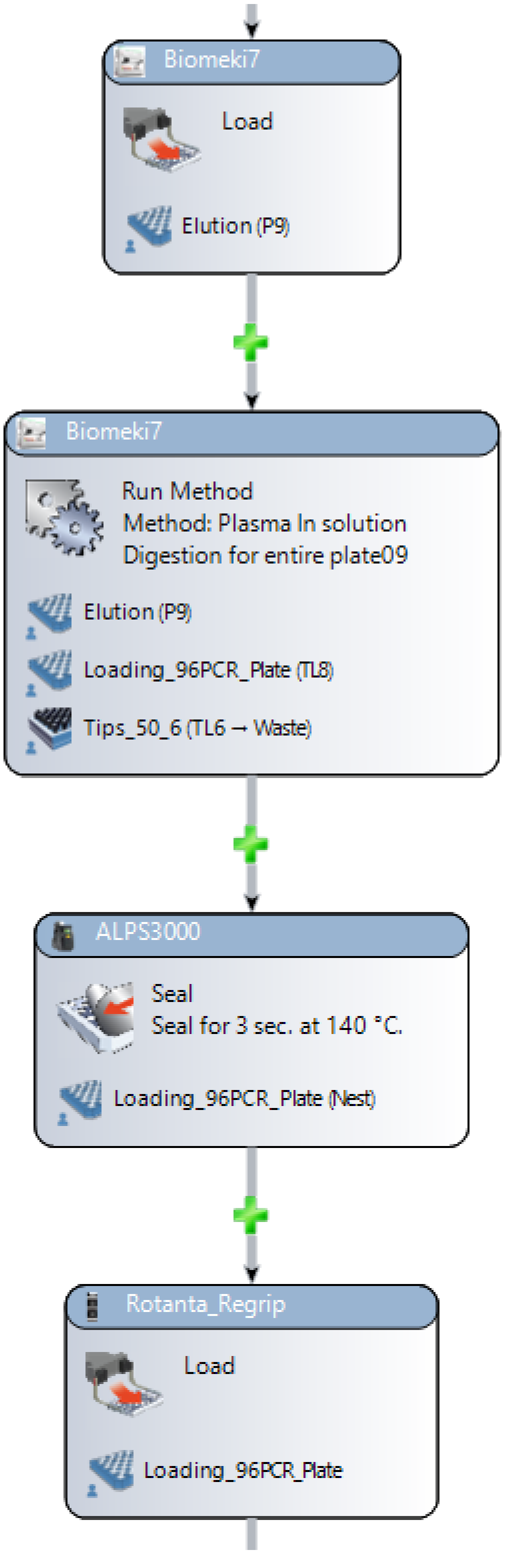

Load the elution plate to Biomek i7.

Transfer peptides from the elution plate to the loading plate by Biomek i7.

Seal the loading plate by ALPS3000.

Load the loading plate to the regrip location.

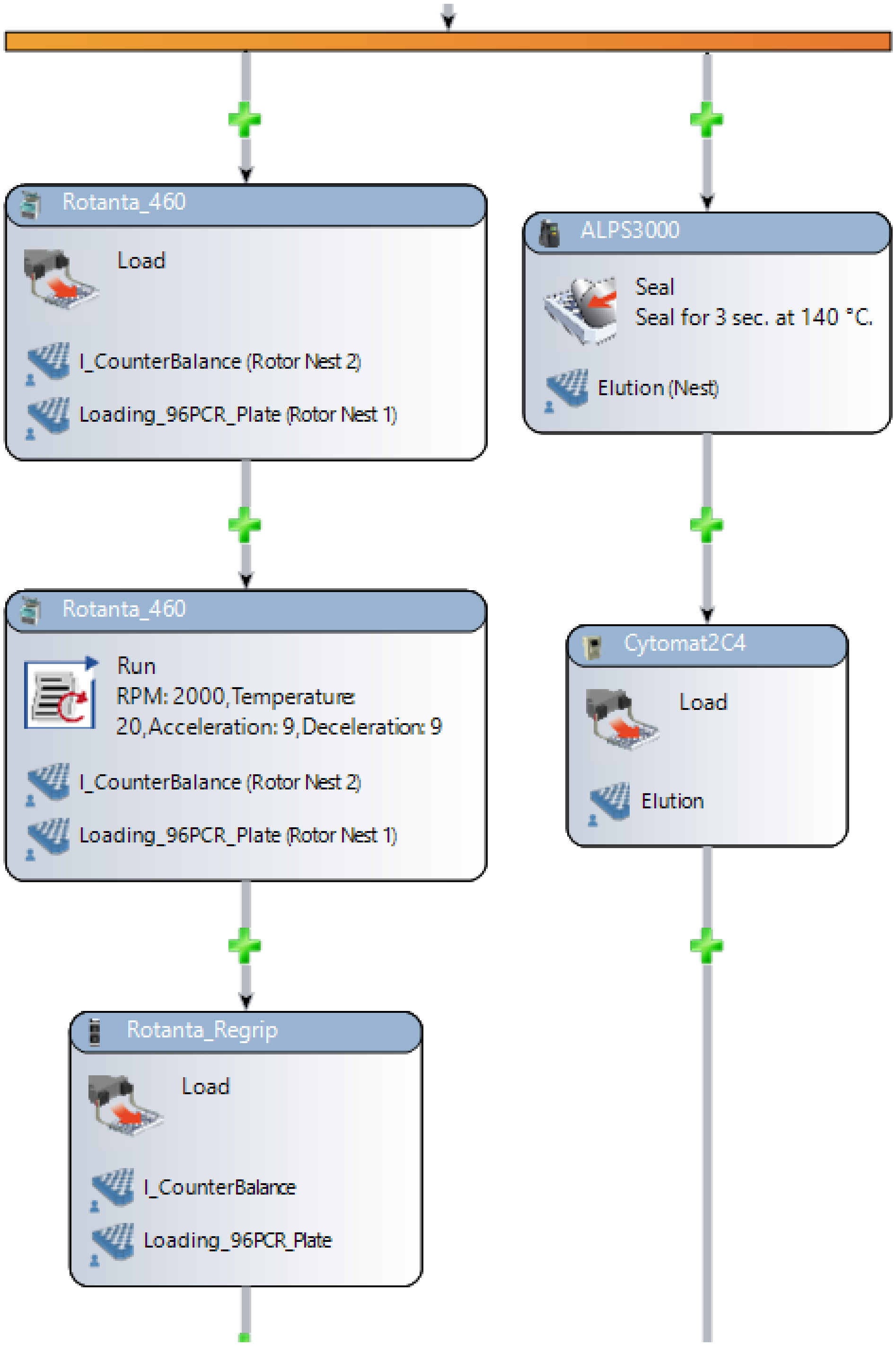

Load the loading plate and the balance plate to Rotanta.

Seal the elution plate by ALPS3000.

Centrifuge the loading plate and the balance plate.

Load the elution plate to Cytomat 2C4 hotel.

Load the loading plate and the balance plate to the regrip location.

Release the balance plate.

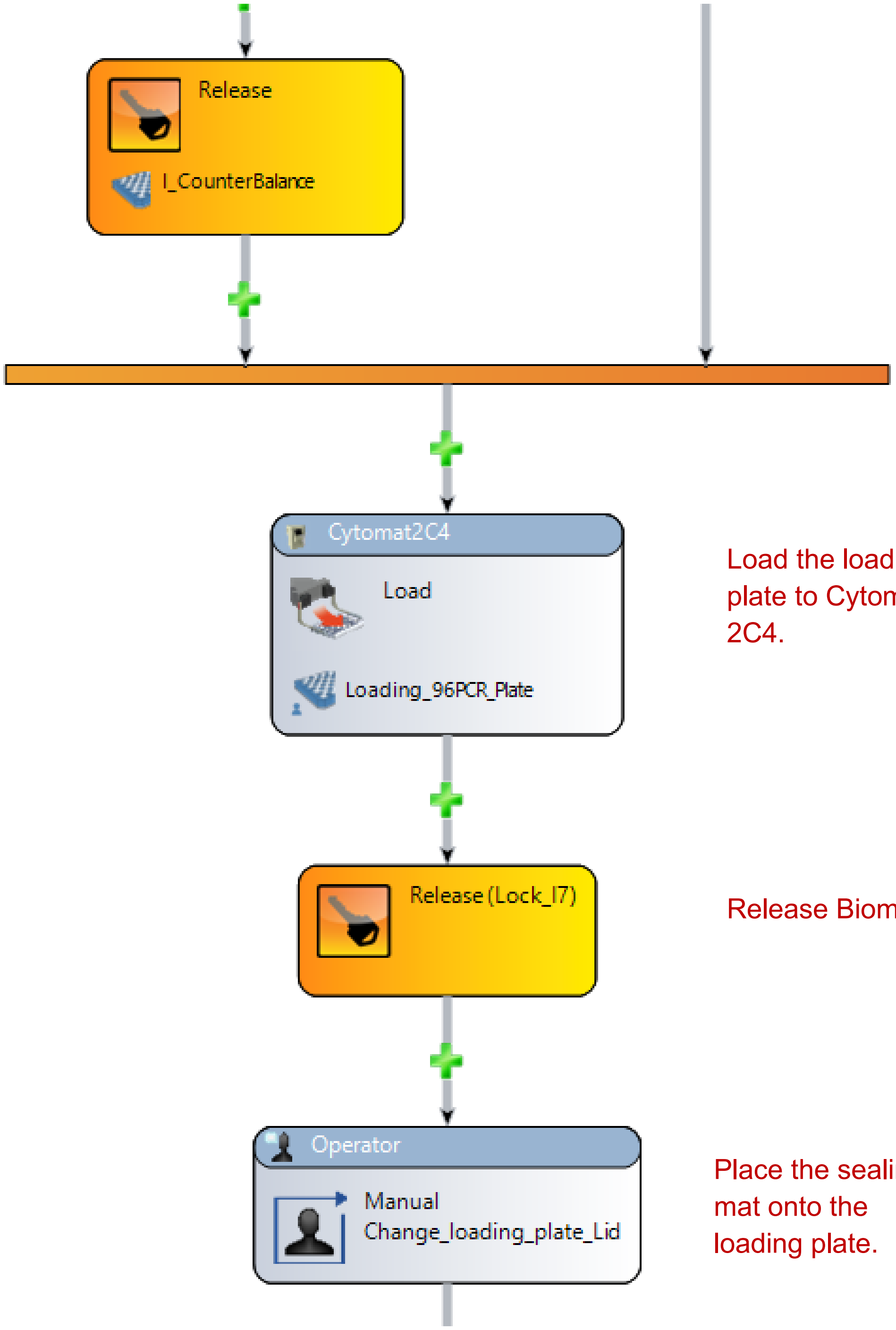

Load the loading plate to Cytomat 2C4.

Release Biomek i7.

Place the sealing mat onto the loading plate.

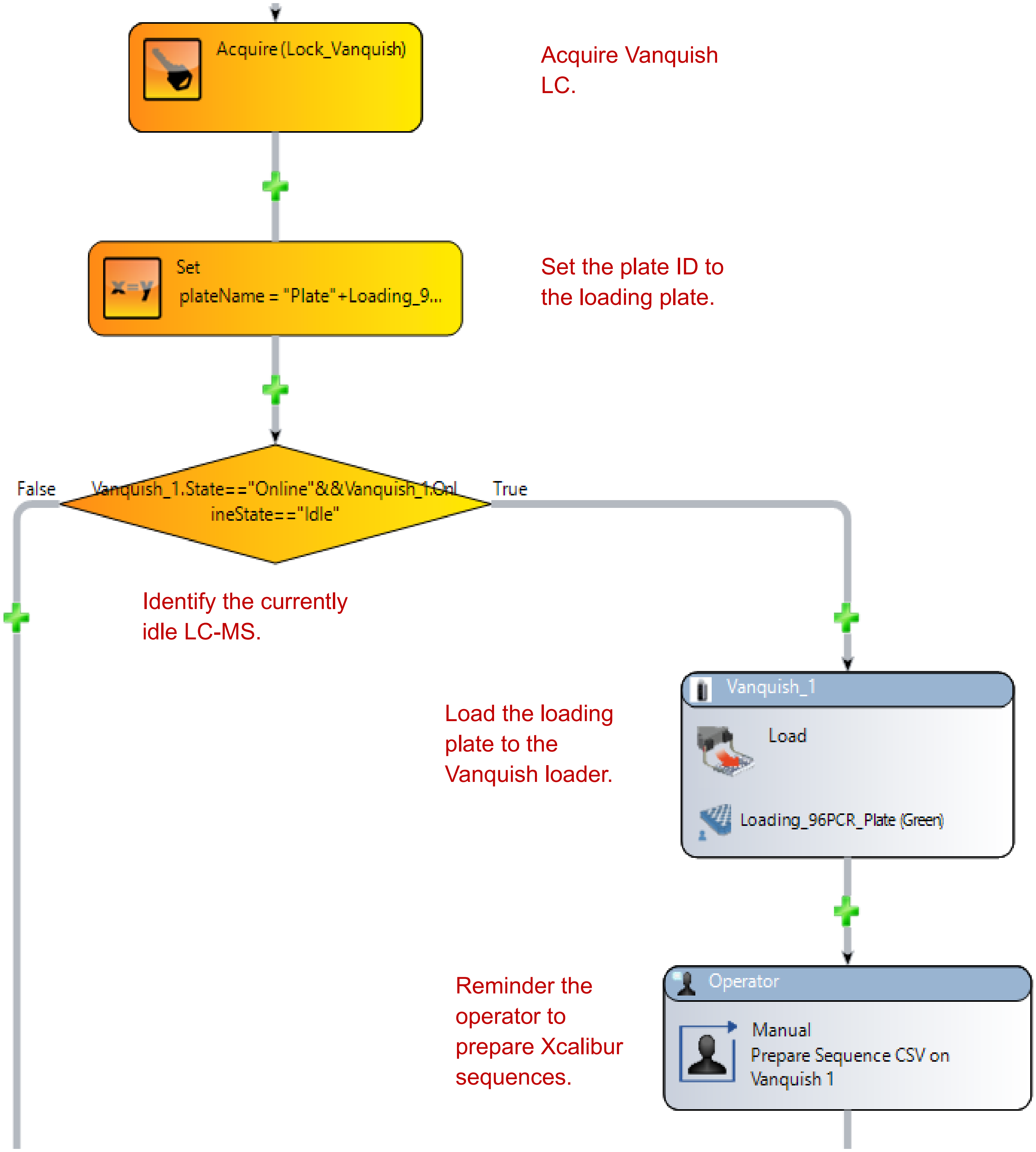
Acquire (Lock_Vanquish)
Acquire Vanquish LC.
Set
plateName = "Plate"+Loading_9...
Set the plate ID to the loading plate.
False
Vanquish_1.State=="Online"&&Vanquish_1.OnlineState=="Idle"
True
Identify the currently idle LC-MS.
Vanquish_1
Load
Loading_96PCR_Plate (Green)
Load the loading plate to the Vanquish loader.
Operator
Manual
Prepare Sequence CSV on Vanquish 1
Reminder the operator to prepare Xcalibur sequences.

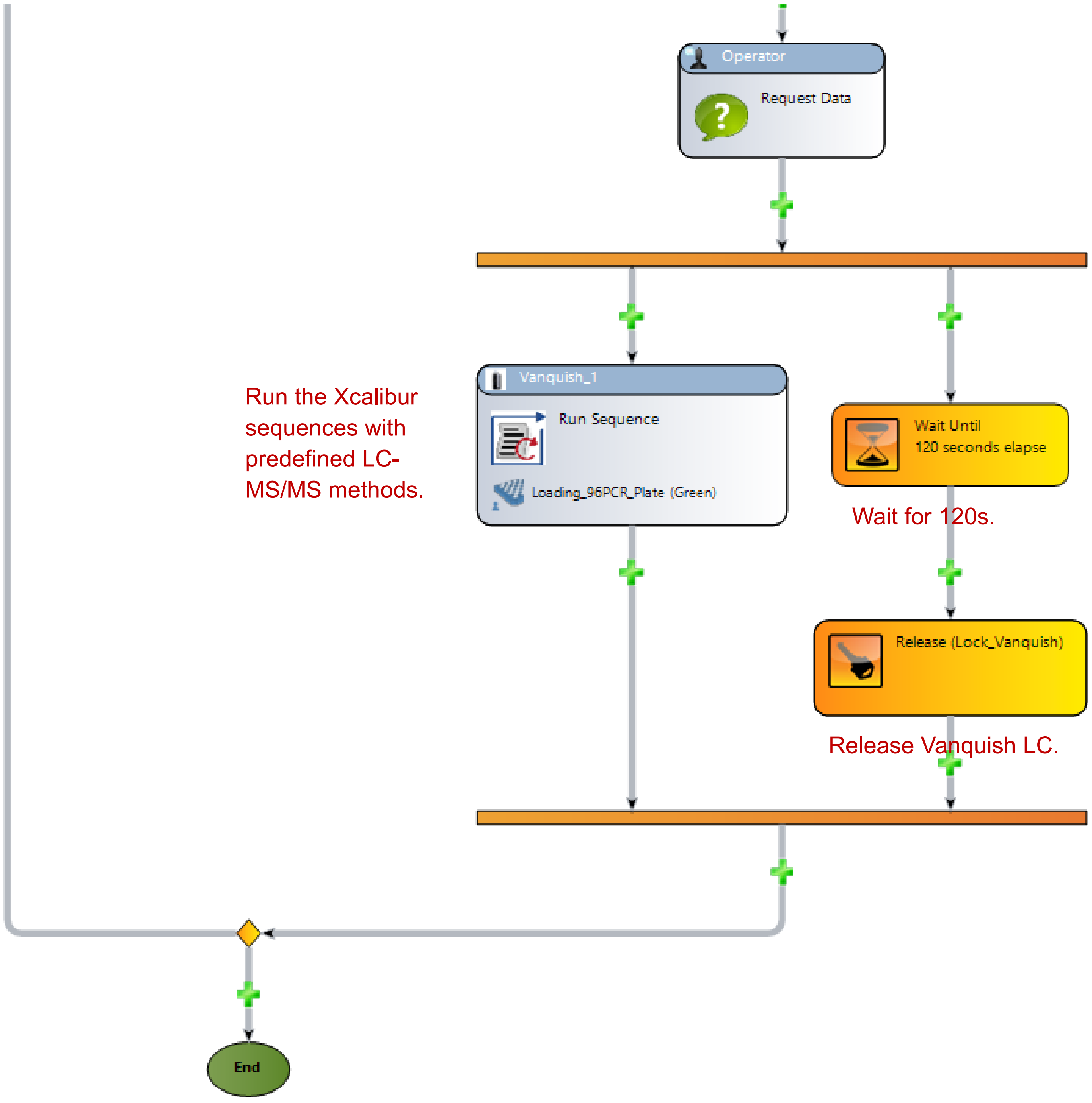
Operator
Request Data
Run the Xcalibur sequences with predefined LC-MS/MS methods.
Vanquish_1
Run Sequence
Loading_96PCR_Plate (Green)
Wait Until
120 seconds elapse
Wait for 120s.
Release (Lock_Vanquish)
Release Vanquish LC.
End
